# Estimating geological CO_2_ storage security to deliver on climate mitigation

**DOI:** 10.1038/s41467-018-04423-1

**Published:** 2018-06-12

**Authors:** Juan Alcalde, Stephanie Flude, Mark Wilkinson, Gareth Johnson, Katriona Edlmann, Clare E. Bond, Vivian Scott, Stuart M. V. Gilfillan, Xènia Ogaya, R. Stuart Haszeldine

**Affiliations:** 1 Geology and Petroleum Geology, School of Geosciences, Kings College, University of Aberdeen, Aberdeen, AB24 3UE UK; 2 School of GeoSciences, University of Edinburgh, James Hutton Road, Edinburgh, EH9 3FE UK; 3 Departament de Dinàmica de la Terra i de l’Oceà, Institut GEOMODELS, Universitat de Barcelona, C/Martí i Franquès s/n, 08028 Barcelona, Spain

## Abstract

Carbon capture and storage (CCS) can help nations meet their Paris CO_2_ reduction commitments cost-effectively. However, lack of confidence in geologic CO_2_ storage security remains a barrier to CCS implementation. Here we present a numerical program that calculates CO_2_ storage security and leakage to the atmosphere over 10,000 years. This combines quantitative estimates of geological subsurface CO_2_ retention, and of surface CO_2_ leakage. We calculate that realistically well-regulated storage in regions with moderate well densities has a 50% probability that leakage remains below 0.0008% per year, with over 98% of the injected CO_2_ retained in the subsurface over 10,000 years. An unrealistic scenario, where CO_2_ storage is inadequately regulated, estimates that more than 78% will be retained over 10,000 years. Our modelling results suggest that geological storage of CO_2_ can be a secure climate change mitigation option, but we note that long-term behaviour of CO_2_ in the subsurface remains a key uncertainty.

## Abstract (web summary)

Carbon capture and storage can help reduce CO_2_ emissions but the confidence in geologic CO_2_ storage security is uncertain. Here the authors present a numerical programme to estimate leakage from wells and find that under appropriate regulation 98% of injected CO_2_ will be retained over 10,000 years.

## Introduction

Limiting global average temperature rise to well below 2 °C above pre-industrial levels, in order to comply with the Paris 2015 Agreement, requires that fossil carbon use is curtailed, and/or large tonnages of CO_2_ must be captured and securely stored underground^1–3^. Despite worldwide interest and the successful implementation of several tens of CO_2_ storage research, pilot and commercial projects^4,5^, some scientists, publics and stakeholders remain concerned that leakage of CO_2_ poses a threat to the viability of long duration CO_2_ storage as an effective climate mitigation tool^6–12^. Leak rates of 0.01% per year, equivalent to 99% retention of the stored CO_2_ after 100 years, are referred to by many stakeholders as adequate to ensure the effectiveness of CO_2_ storage^1,13,14^. We assert that secure storage must allow global average temperature rise, driven by excess CO_2_, to remain well below 2 °C, and for geological timescales, typically modelled to be at least 10,000 years^15^.

However, there is a lack of quantitative predictions on long-term CO_2_ storage security and the likelihood of this target being achieved, beyond the individual site scale, and across a global CO_2_ storage industry. Many studies that assess the global industry-wide risk of subsurface gas leakage do not specifically assess subsurface CO_2_ retention mechanisms^16,17^, despite experimental measurements showing that residual trapping may effectively immobilise a significant proportion of the CO_2_ almost immediately on injection into the subsurface^18^. The published studies that incorporate subsurface CO_2_ retention into their risk assessments are for site-specific, real or hypothetical, hydrogeological models^19,20^, rather than industry-wide, regional, or global scenarios. A recent tool developed by the National Risk Assessment Partnership (NRAP) applies a system-modelling approach and Monte Carlo analysis to detailed subsurface storage reservoir models^21^. This tool can provide long-term storage security predictions and uncertainties for individual sites, but to date, no comprehensive case studies have been published that facilitate an industry-wide assessment of CO_2_ storage security.

In order to address this gap in knowledge and prediction assessment, we present a new numerical program–the Storage Security Calculator (SSC). The SSC has been designed to determine if global adoption of geological CO_2_ storage will be secure enough, and secure for long enough, to effectively mitigate climate change. This program uses two routines: one using established and measured geological processes to assess retention in the geological reservoir; the second calculating surface leakage flux rates, which vary through time. The input parameters for these routines are derived from a data compilation, based on extensive literature review of empirically measured data and simulated data, encompassing CO_2_ immobilisation, and surface leakage of CO_2_ or appropriate analogues. A summary of this review is provided in Supplementary Notes 2–8.

Practical CO_2_ storage is already undertaken in both onshore and offshore environments, with each exhibiting differing implementation and operational challenges. We thus apply the SSC to three different scenarios, that differ in their input parameters. For regional implementation of CO_2_ storage in a realistically well-regulated industry, with a moderate density of legacy wells, our program calculates a 50% probability that more than 98% of the injected CO_2_ will remain trapped in the subsurface over 10,000 years. Applying the SSC to a worst-case, unrealistic scenario of CO_2_ storage being inadequately regulated and implemented in a region with a high risk of leakage along abandoned wells, calculates that at least 78% of the CO_2_ will be trapped in the subsurface over 10,000 years. These results assume that our data compilation and subsequent input parameters are representative of the scenarios we modelled, and the SSC has thus been designed to accept new, improved, or case-specific input parameters, as appropriate. Key uncertainties remaining in the program include a lack of empirical data, and thus understanding, of CO_2_ behaviour in the subsurface over the thousands of years timescale, long-term behaviour of abandoned wells as fluid migration pathways, and long-term evolution of leakage rates.

## Results

### Storage security calculator overview

**Fig. 1:**
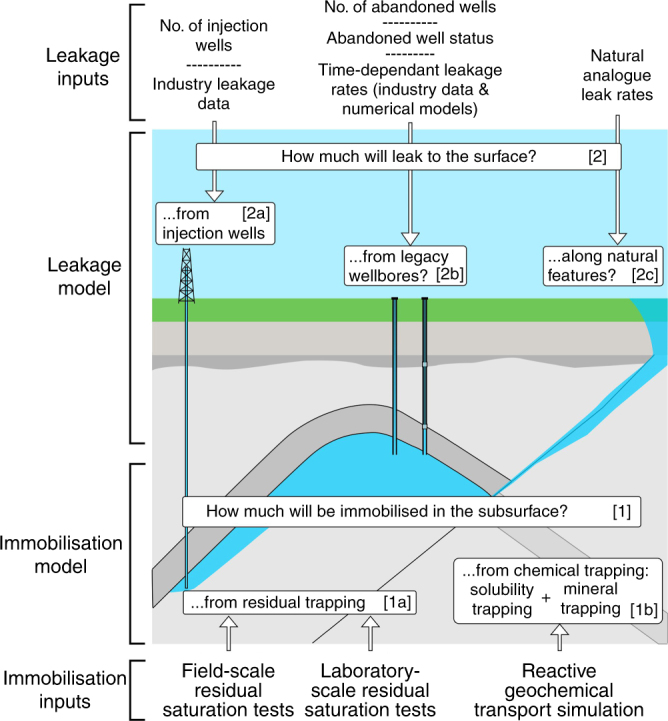
The Storage Security Calculator concept. The CO_2_ immobilisation model [1] combines two sub-models: [1a] residual trapping, and [1b] chemical trapping (defined as a combination of solubility and mineral trapping). The CO_2_ leakage model [2] combines three sub-models: [2a] leakage through active (injection) wells, [2b] abandoned wells, and [2c] leakage via natural pathways. The key input parameters for each sub-model are shown

**Fig. 2:**
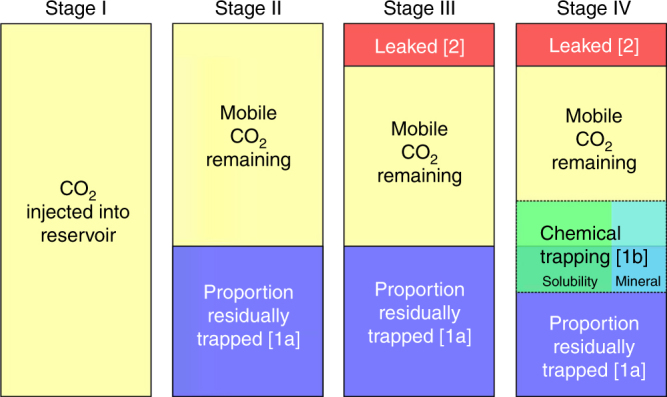
Modelling stages computed by the Storage Security Calculator. The immobilisation and leakage models described in Fig. 1 are integrated in the Storage Security Calculator to compute the proportion of remaining mobile CO_2_ in the subsurface in four stages. I) The total amount of CO_2_ injected into reservoir is computed (based on the storage target). II) The amount of CO_2_ immobilised by residual trapping is calculated (residual trapping immobilisation model – [1a]). III). The amount of CO_2_ leaked from the reservoir (and, for simplicity, assumed to reach the atmosphere) is calculated via the leakage model. IV) The amount of CO_2_ immobilised by chemical trapping (chemical trapping immobilisation model) is calculated as a function of free-phase (i.e., both residual and mobile) CO_2_ remaining in the reservoir. The calculations are carried out annually for each time-step of the model until there is no mobile CO_2_ remaining, or until 10,000 years, whichever happens first

The SSC is designed to quantify the immobilisation of CO_2_ injected into the subsurface for geological storage and the total CO_2_ leakage to the atmosphere. Once injected, the CO_2_ will be subject both to immobilisation processes ([1] on Fig. 1) and to potential leakage (migration out of the reservoir and subsequent leakage to the atmosphere-[2] on Fig. 1). As this study focuses on assessing the effectiveness of CO_2_ storage for climate mitigation, the SSC solely quantifies the leakage of CO_2_ to the atmosphere, rather than migration of CO_2_ into secondary subsurface environments. This numerical program, implemented in the programming language R, combines immobilisation and leakage models (Figs 1 and 2). The amount of immobilised and leaked CO_2_ are calculated over time and subtracted from the total injected CO_2_ to yield the mobile (i.e., potentially leakable) CO_2_ remaining in the reservoir. The integrated program runs until 10,000 years, with leakage ceasing once no mobile (leakable) CO_2_ remains in the reservoir (see Methods).

The SSC is comprised of two core elements, a CO_2_ immobilisation model (1), and a CO_2_ leakage model (2). The CO_2_ immobilisation model (see [1] on Fig. 1) computes quantities and rates of residual saturation, solubility in subsurface brine, and reactions that precipitate solid minerals. All these processes result in permanent trapping of CO_2_ in the subsurface. Two sub-models consider the impact of residual trapping (see [1a] on Fig. 1), and of chemical trapping (here defined as a combination of solubility and mineral trapping–[1b] on Fig. 1). Combining these two sub-models computes the proportion of CO_2_ immobilised in the subsurface, and thus unavailable to leak. The leakage model (see [2] on Fig. 1) calculates fluxes of CO_2_ leaking to the surface from depth, and is independent of subsurface conditions. It combines three leakage sub-models that quantify leakage from active (injection) wells (see [2a] on Fig. 1), abandoned wells (see [2b] on Fig. 1), and natural pathways (see [2c] on Fig. 1). Parameters for each of these models are based on measured surface risk and fluxes of subsurface gases from analogues, which are used to calculate a conservative, initial surface leakage rate. Conservative values are adopted for each leakage input parameter to ensure that the SSC does not under-estimate leakage risk (see Supplementary Notes 3–5). Our adopted leakage fluxes are based on observed fluxes of CO_2_, oil, and natural gas, with most of the data representing natural gas leakage (see Supplementary Notes 3–5). A recent study investigated how the Aliso Canyon blowout would have proceeded if it had been leaking CO_2_ rather than natural gas, and concluded that leakage would have been lower due to a higher density of CO_2_ compared to methane, and resulting differences in thermodynamic and flow properties^22^. Our estimates for leakage parameters do not yet account for this, due to uncertainties in quantifying the magnitude of the difference between CO_2_ and methane blowouts. It is thus likely that real world leakage will be lower than calculated by the SSC.

The calculated leakage rate is an initial, maximum rate that reduces over time. The rate reduction is based on modelled and observed rates of leaking CO_2_ and methane, as well as measured natural gas production rates (see Methods and Supplementary Note 6 for datasets used); it represents a real-world response to changes in subsurface conditions, such as reservoir pressure dissipation, and reduction in the buoyancy of the brine and CO_2_ fluid as the column height and proportion of mobile CO_2_ is reduced^1^.

Residual trapping occurs on geologically instantaneous timescales, and can be measured in laboratory and field tests over experimental timescales^18,23,24^. Hence, when free-phase CO_2_ contacts the pore-space, a proportion of that CO_2_ will become residually trapped, and thus unable to leak (immobilised). Leakage can also occur instantaneously, but it cannot remove CO_2_ from the reservoir that has already been residually trapped. The chemical trapping model includes both solubility and mineral trapping and is based on a published trapping model^25^ that incorporates both solubility and mineralisation in a time scale appropriate for CO_2_ storage. Figure 2 shows a schematic representation of the steps used to calculate leakage and immobilisation by the SSC.

The SSC uses three different calculation techniques. The Base Case Scenario applies a single value for each input parameter determined as the most likely conservative value by the authors, based on currently available data (see Methods). A Monte Carlo analysis applies ranges of values for most parameters (normal, lognormal, uniform, and triangular distributions as appropriate for each dataset) and facilitates an assessment of the overall uncertainty of the model results. Finally, a sensitivity analysis is carried out where only one parameter is varied across multiple simulations, allowing an assessment of the influence and uncertainties of each parameter to the model. Further details on the program and parameters are provided in the Methods section and the R-code is available in Supplementary Data 1.

### Overview of the immobilisation model

There are four trapping mechanisms that retain injected CO_2_ in the reservoir: structural and stratigraphic, residual, solubility, and mineral trapping^1^. The process of structural and stratigraphic trapping prevents injected CO_2_ migrating to the surface due to impermeable layers of rock being present above the CO_2_ reservoir. CO_2_ remains in the permeable reservoir as a free-phase (gas or supercritical, depending on reservoir conditions) and could be contained indefinitely in this state in a secure trap^26^. However, this mechanism does not immobilise the CO_2_ in the subsurface permanently, and a failure in storage integrity, via caprock or well failure, could allow the CO_2_ to migrate out of the reservoir. Structural and stratigraphic trapping are implicitly invoked in the SSC, so that all mobile CO_2_ remaining in the reservoir is assumed to be structurally/stratigraphically trapped.

The three other trapping mechanisms–residual, solubility and mineral trapping–are more secure and would require deliberate engineering to reverse. For example, displacing residually trapped CO_2_ would require injection of a fluid to re-mobilise the CO_2_ in much the same way that tertiary oil production (i.e., enhanced oil recovery (EOR)) is conducted. Solubility trapping may only be reversed by significant reservoir depressurisation, and even then, a subset of this would be irreversible in the form of ionic trapping, where aqueous CO_2_ has dissociated and is now in bicarbonate or carbonate ion form^27^. Reversing mineral trapping, where CO_2_ precipitates as carbonate minerals, would require dissolution of these minerals and hence is the most secure form of trapping^27^.

The SSC incorporates separate sub-models for residual trapping (1a), and for chemical trapping (1b–combined solubility and mineral trapping). The residual trapping sub-model (1a) draws on 44 residual trapping values compiled by Burnside and Naylor^18^, who calculated the proportion of residually trapped CO_2_ from experimental residual CO_2_ saturation values. These data indicate that residual trapping immobilises between 12.8% and 91.6% of the injected CO_2_. Residual trapping values are also now available for a number of reservoir-scale experiments. The Otway 2B experiment (Paaratte Formation, Australia) used a range of techniques (pulsed neutron logging, noble gas tracers, and oxygen isotope fractionation) to calculate residual CO_2_ pore space saturations of between ~7% and 42% over two separate experiments in 2011 and 2014^23,28,29^. These values are comparable to the residual CO_2_ saturation of 33%, determined by core flooding experiments on a sample of Paaratte Formation sandstone^24^ (corresponding proportion of CO_2_ that was residually trapped = 55.9%^18^), and indicate that laboratory-scale residual trapping experiments are representative of reservoir-scale conditions.

Solubility trapping via CO_2_ dissolution occurs on timescales of hundreds of years. Whilst this is essentially instantaneous on geological timescales, it is a slower process than residual trapping and leakage. Mineral trapping is a much slower process that occurs over the 1000-year timescale. Hence, chemical trapping (1b) is computed in the SSC following residual trapping (1a) and leakage (2) calculations. Chemical trapping consumes both mobile and residually trapped CO_2_, so the amount of residually trapped CO_2_ decreases over time. The chemical trapping sub-model (1b) is based on the reactive transport equilibrium simulation carried out by Xu et al.^25^, which simulates the transfer of injected CO_2_ between free-phase (gas), dissolved (aqueous) and mineralised (solid) phases, over 10,000 years. To the best of our knowledge, this^26^ is the only model in the literature that quantifies both dissolution and mineral trapping rates over geological storage timescales specifically for CO_2_ storage (see Methods and Supplementary Note 8). This means we cannot apply a sensitivity analysis to the chemical trapping model section of the SSC (1b) as uncertainty cannot be quantified for this part of the model.

### Overview of the leakage model

Our leakage model is based on assessments of volumes of subsurface fluids leaked from: active hydrocarbon industry wells (analogous to CO_2_ injection–2a); abandoned wells (i.e., legacy hydrocarbon industry wells–2b); and natural examples of gases leaking from geological features (e.g., faults or poor caprock integrity–2c). For a given storage reservoir, the degree of leakage along a fluid migration pathway will depend on a number of factors, including: the areal density and depth of the migration pathways, proximity of the migration pathway to the injection well, plume geometry, reservoir pressure, free-phase CO_2_ column height, the relative permeability of all geological formations and migration pathways, capillary entry pressure, fluid pore pressure, hydrodynamic flow regime, and temperature^30–35^. Precise modelling of potential leakage along migration pathways at a given storage site requires detailed constraints on all of these parameters, injection volume and pressure, and appropriate model-grid spacing and equations of state^30–32^. Generalising these factors to estimate global or regional storage security is unrealistic, and so we base our estimates on a combination of directly measured surface fluxes of subsurface fluids leaking from depth (via wells and fault systems) and published numerical leakage models. This approach allows resolution of a globally averaged surface flux that is independent from the multiple complex factors of specific subsurface conditions (see Supplementary Notes 2–6 for further details).

To date, industrial experience related to CCS is limited to six commercial-scale dedicated CO_2_ storage sites (Sleipner and Snøhvit, Norway; Aquistore and Quest, Canada, In Salah, Algeria, and the Illinois Industrial Carbon Capture and Storage (IICCS) project, USA). This is supplemented with experience from a number of large tests such as Lacq (France)^36^ and Ketzin (Germany)^37^ and multiple CO_2_ EOR projects which have operated since the early 1970s^5,38^. No leakage has yet been detected from dedicated CO_2_ storage projects so direct leakage data does not exist. We therefore base our active (2a) and abandoned (2b) well leakage estimates on data from the wider hydrocarbon industry, including underground gas storage (UGS) and EOR. Geological storage of CO_2_ employs expertise, techniques and technology from the hydrocarbon industry, making this industry a suitable analogue of the CO_2_ geological storage technology^39,40^. Input parameters for active and abandoned well leakage include the frequency of leakage events, (continuous leakage and discrete events/blowouts), and mass lost per leaking well during a leakage event. For each scenario, assumptions are made about the quality of the engineered well barriers (casing and plugging–see Supplementary Note 3 for a description of barrier features), and these are used to determine appropriate event frequencies. (Supplementary Notes 3 and 4). To estimate CO_2_ leakage along natural pathways (2c) we utilise measured areal fluxes of CO_2_ and natural gas from areas containing natural gas seeps at regional to global scale, to provide a mass per km^2^ per year for natural leakage (Supplementary Note 5).

In any highly explored subsurface area with numerous wells, it can be difficult to identify all abandoned wells and to determine their integrity and associated leakage risk^20,41,42^. Hence, abandoned wells are expected to be a significant hazard to CO_2_ storage security^43^ and thus receive considerable attention in our SSC tool, varying between the modelled scenarios. In regions with a long-lived hydrocarbon industry, there may be instances of wells not being recorded and being improperly abandoned. For example, a recent study of Pennsylvanian hydrocarbon industry wells revised estimates of legacy well numbers from 350,000 wells to up to 750,000^44^. In this Pennsylvania example, an under-estimation factor of ~2.1 describes the difference between recorded and existing wells. In a well-regulated industry, CO_2_ storage site operators will be required to identify all local wells. In well-regulated regions, we expect all abandoned wells to have been documented, so that the under-estimation factor will be 1. In regions with a poorly regulated hydrocarbon industry, undocumented abandoned wells may not be identified by site surveys. Therefore, we have built an under-estimation factor into the SSC that allows quantification of the impact of unidentified abandoned wells. For our Offshore, and Onshore Well-Regulated scenarios, the under-estimation factor is 1. For our Poorly-Regulated Onshore scenario, we adopt a base-case under-estimation factor of 1.55, with a minimum and maximum of 1.1 and 2.0, respectively (see Methods).

Collated data from well leakage simulations and from measurements of natural gas production rates and a long-lived blowout show that leakage rates decrease through time in an approximately exponential manner. This is due to the leakage itself depleting the available volume of free CO_2_, combined with CO_2_ immobilisation and pressure dissipation within the subsurface. To incorporate leakage decay into our model, we created two exponential decay curves (see Methods, Eq. (3)) that form an envelope to the data. The longest data set is that of a Zahasky and Benson model (500 years)^45^, while other models and measurements are for much shorter, decadal timescales. To avoid inaccuracies in extrapolating the leakage reduction curves forward in time beyond the range of data, our curves assume that leakage rate decreases to a point, and then remains constant over time. The exponential decay curves (Eq. (3)) are a function of model parameters A and B, which were iteratively determined to produce curves that envelop the data. Parameter A represents the minimum long-term leakage rate as a percentage of the maximum. For the Monte Carlo analysis, a triangular distribution is assumed for Parameter A; this means that the value we judged most likely, and used in the base case simulation, is different from the mean and median of the distribution. Parameter B is the exponential function of the equation that defines the leakage reduction (see Methods and Supplementary Note 6).

### Overview of modelled scenarios

The geological and engineering factors relating to leakage of subsurface gases are generally well understood. Hence, regional differences of leakage risk are well identified in historic subsurface industry activity and regulation. We therefore apply the SSC to three hypothetical scenarios that investigate implementation of CO_2_ storage in a large global region: a Well-Regulated Offshore Scenario, a Well-Regulated Onshore Scenario, and a Poorly Regulated Onshore Scenario.

The Offshore Scenario can be considered to be analogous to CO_2_ storage beneath the North Sea, which is the most likely store for CO_2_ emissions captured in the EU. The Well-Regulated Onshore Scenario can be considered analogous to Texas, USA, which has a mature and well-regulated hydrocarbon industry in place. The Poorly-Regulated Onshore Scenario represents the highly unlikely event of CO_2_ storage being implemented in regions or countries with long-duration, poorly regulated hydrocarbon industries leading to difficulties in identifying legacy abandoned wells, or enforcing applicable regulation. For each scenario we investigate the injection and storage of a large cumulative tonnage of CO_2_ (12 Gt), comparable to the 2050 storage target of the European Union^46,47^. Injection commences in 2020, finishes in 2050, and the SSC is run for 10,000 years into the future. CCS will very likely be required to continue well beyond the year 2050^47,48^, but for simplicity we focus on modelling CO_2_ storage security during the initial decades of CCS implementation. The three scenarios vary in their model parameter inputs: Onshore and Offshore Scenarios differ in the frequency of leaking injection wells, abandoned well areal density, and abandoned well integrity; the Onshore Well-Regulated and Poorly-Regulated scenarios differ in the proportion of unidentified abandoned wells, and the integrity status of abandoned wells.

The Offshore Scenario uses the North Sea as an exemplar of a CO_2_ storage environment. The assigned abandoned well density (0.44 wells km^−2^) is based on recent well densities of the North Sea (4400 wells per 10,000 km^2^)^1^. Abandoned well integrity and frequency of leaking wells are based on data from offshore hydrocarbon fields. The Onshore Well-Regulated Scenario uses Texas as a CO_2_ storage environment exemplar, with an abandoned well density of 2.5 wells km^−2^, based on estimates of the number of hydrocarbon wells in Texas^41^, and well integrity and leakage risk based on data from onshore hydrocarbon fields. The Poorly-Regulated Onshore Scenario investigates CO_2_ storage security if implemented in a region with inadequate regulations (either past or current) regarding drilling and abandonment of wells. For this scenario, we use Pennsylvania (USA) as an exemplar due to the high number of undocumented legacy wells (to give a well under-estimation factor) and the proportion of abandoned wells that are unplugged (see Supplementary Note 4 for further details).

### Base case results

**Fig. 3:**
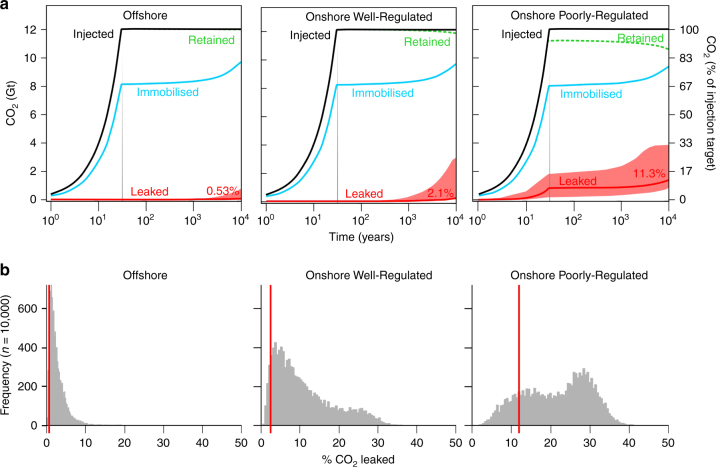
CO_2_ storage modelling results for the three target scenarios. **a** Evolution of CO_2_ immobilisation and leakage over time, for the three base case scenarios. The black line shows the total CO_2_ injected, between 2020 and 2050. The grey line shows when injection ceases. The blue line shows CO_2_ retained by immobilisation due to rapid residual trapping and due to longer-timescale solubility and mineralisation trapping. The green dashed line is the cumulative limit of CO_2_ retention due to leakage of CO_2_ out of the subsurface, for example through faults or leaking wells, and is the inverse of leakage (red line). Between the blue and green lines is CO_2_ retained in the reservoir by structural/stratigraphic trapping. Most CO_2_ loss occurs through leaking wells. Red line at base of graph shows the base case result of cumulative leakage through time, with shaded P5 to P95 distribution envelopes above and below as derived from Monte Carlo analysis. The red number is the base case percentage cumulative leakage at 10,000 years, cf Table 1. **b** Histograms showing the distribution of results from Monte Carlo analysis (10,000 realisations) for each scenario; results are cumulative leakage as a percentage of the total CO_2_ injected at model year 10,000 (*x*-axis). Red vertical lines show the base case scenario cumulative leakage result at 10,000 years (red numbers in graphs above, and Table 1)

SSC modelled results are presented as the proportion of CO_2_ leaked, relative to the amount injected, over time for the three scenarios in Table 1. These are cumulative results that sum the amount of CO_2_ leaked per year. Base case results for immobilisation and retention of the CO_2_ are shown graphically in Fig. 3 along with base case and Monte Carlo results (90% confidence envelope) for leakage.

**Table 1:** Storage security calculator outputs

Scenario^a^	Time (year)	CO_2_ leaked (%)^b^			
		Base case^c^	P95^c^	P50^c^	P05^c^
Offshore Well-Regulated	1	0.000755	0.000506	0.000779	0.00144
	100	0.0286	0.0249	0.0447	0.0888
	1000	0.0744	0.0709	0.213	0.646
	10,000	0.532	0.483	1.89	6.29
Onshore Well-Regulated	1	0.00211	0.00133	0.00217	0.00451
	100	0.0861	0.0737	0.156	0.358
	1000	0.269	0.246	0.888	2.96
	10,000	2.10	1.81	8.18	25.71
Onshore Poorly Regulated	1	0.215	0.0517	0.202	0.521
	100	6.71	1.70	6.41	16.5
	1000	7.12	2.39	8.05	20.0
	10,000	11.3	6.91	22.0	32.6

The leakage values are expressed as a percentage of originally injected CO_2_. Example times are presented at *t* = 1, 100, 1000 and 10,000 years

^a^Three scenarios are represented, to illustrate regional storage security decreasing from well-regulated to poorly-regulated

^b^The total leakage percentages are calculated by adding together all the yearly increments of leaked CO_2_ calculated for each model run. Values reported to three significant figures

^c^Four probabilities of CO_2_ leakage are chosen to be represented: a Base Case, where the model parameters are selected by expert judgement, and Monte Carlo results of sampling the whole probability range of each parameter in the Immobilisation and Leakage model datasets. P95 means that 95% of the calculated leakage values are greater than the percentage calculated (not a 90% probability of occurrence), P50 represents that 50% of values will be greater (the median), and P05 means that 5% of the calculated leakage values from the original total injected (not a 10% probability) are greater than the calculated percentage. Conventional reporting of statistics of subsurface hydrocarbon reserves and resources, or of the greatest possibility of an outcome, use P50 (the median) as the most probable outcome

Base case cumulative leakage, as a percentage of the total injected, at 10,000 years are 0.532% for the Offshore Scenario, 2.10% for the Onshore Well-Regulated Scenario, and 11.3% for the Onshore Poorly-Regulated Scenario. These base case results equate to simplified time-averaged linear leak rates of 0.00005, 0.0002 and 0.001% per year, respectively. Well Regulated Offshore sites perform best, due to leakage being limited by a low density of abandoned wells. Onshore sites perform equally well with geological immobilisation in the reservoir, but have higher leakage rates due to a greater density of abandoned wells; to ensure we do not under-estimate leakage, we have assumed that intact abandoned wells experience a very low long-term leak rate (0.004 t per year; see Supplementary Note 4 for discussion). Cumulative leakage in the Well-Regulated Onshore Scenario is dominated by long-term leakage via abandoned wells. Conversely, cumulative leakage in the Poorly-Regulated Scenario is governed by blowouts in improperly abandoned wells during the injection period.

### Monte Carlo analysis results

If the SSC model is now used in Monte Carlo mode to calculate outcomes, then retention and leakage calculations can be made by sampling the distributions of the full range of possibilities over 10,000 realisations of the model. The P50, P95 and P05 percentiles of the calculated leakage from the 10,000 model runs are reported in Table 1, which are the equivalent of the median (P50) and 90% confidence envelope (P95 to P05) of the computed results. These can also be portrayed as leakage exceedance values through time as shown in Fig. 3. For each scenario, Base Case leakage results are lower than the P50 percentile of the Monte Carlo analysis; this is an inevitable statistical outcome due to applying a triangular distribution to the minimum long-term leakage rate (Parameter A) of the leakage reduction function.

**Fig. 4:**
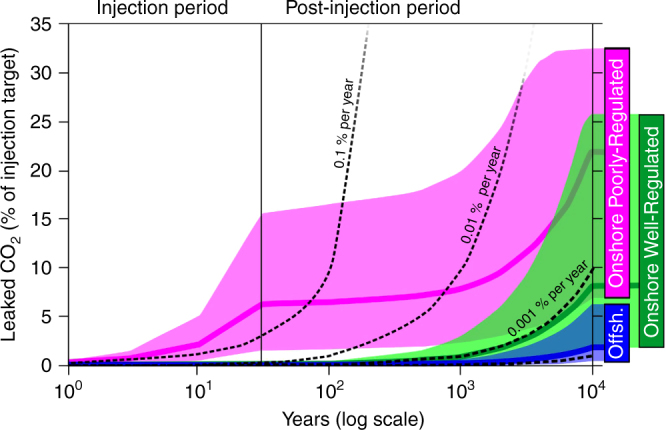
Monte Carlo runs of the three scenarios. The graphs show cumulative leakage of CO_2_ as a percentage of the total injected. Each scenario shows the P50 output as a blue, green, or magenta line, with shading above showing the P05 limit, and below for the P95 limit, defining occurrence envelopes for the three scenarios. These are calculated on 12 Gt CO_2_ injected between 2020–2050, with subsequent storage and leakage over 10,000 years. Black dotted lines show comparisons based on time averaged yearly constant leak rates for 0.1, 0.01, 0.001 and 0.0001% per year (the latter represented by the unlabelled dotted line at the base of the diagram). Offshore Well-Regulated storage (blue) is found to be the most reliable Scenario. Onshore Well-Regulated storage (green) exhibits higher leakage due to a higher density of abandoned wells acting as potential leakage pathways. Poorly-Regulated Onshore storage (magenta) exhibits the highest leakage rates in the short term due to the prevalence of unidentified abandoned wells that are unplugged or degraded

Amounts of CO_2_ leakage over the three Scenarios are shown in Fig. 4, which also highlights the 5th, 50th and 95th percentiles for each Scenario. The Offshore Well-Regulated Scenario shows the least leakage, where 95% of values (P05) are less than 6.29% leakage of total CO_2_ injected at 10,000 years, and 50% of simulations (P50) are less than 1.89% leakage.

The Onshore Well-Regulated Scenario shows that total cumulative leakage remains low at the 1000 years scale (P50 at 1000 years is 0.888% of the total injected CO_2_), but leakage continues over the subsequent 9000 years resulting in higher total cumulative leakage (P50 at 1000 years is 8.18%). This results in 95% of leakage simulations leaking less than 25.71% over 10,000 years. This is despite a lower modelled frequency of leakage from injection wells, and a lower proportion of degraded abandoned wells in the Onshore Well-Regulated Scenario compared to the Offshore Well-Regulated Scenario. However, the Onshore scenarios have a much higher areal density of abandoned wells (2.5 wells km^−2^ as opposed to 0.44 wells km^−2^ for the Offshore Scenario); while leakage rates from individual abandoned wells remain low for the Well-Regulated Scenario, the cumulative leakage for such a high well density becomes significant.

For the Onshore Poorly-Regulated Scenario, 95% of leakage calculations do not exceed 32.6% of the total injected. Somewhat surprisingly, this is only ~7% more than for the Well-Regulated Scenario, despite the Base Case simulation producing more than twice as much leakage. This is because the highest leakage rates simulated in the Monte Carlo analysis of the Poorly-Regulated Scenario result in all of the CO_2_ in the reservoir being either immobilised or leaked before the simulation ends at 10,000 year. For the 95^th^ percentile, leakage essentially stops after approximately 7000 years, as no mobile CO_2_ remains in the reservoir. This is illustrated by the plateauing of the 95th percentile curve of the Poorly-Regulated Scenario in Fig. 4. This demonstrates the failsafe nature of geological CO_2_ storage: even a worst-case leakage scenario results in a most likely (base case) outcome that 89% of injected CO_2_ is permanently stored (Fig. 3). This scale of retention is confirmed by full Monte-Carlo modelling, where the P50 results shows 22.0% leakage-i.e., over 78% of the injected CO_2_ is retained in the subsurface after 10,000 years (Fig. 4).

**Fig. 5:**
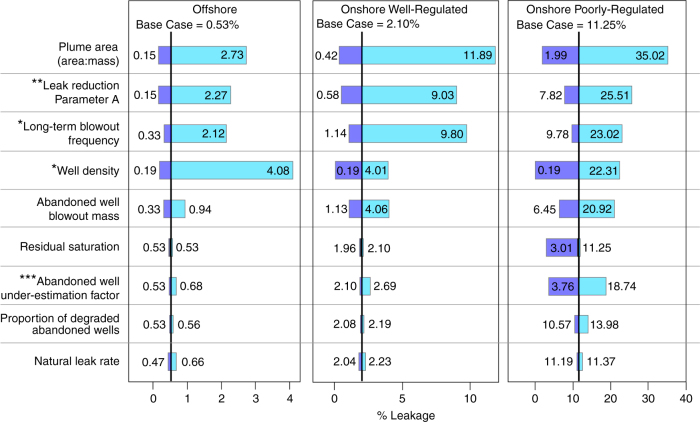
Sensitivity tests of the input parameters. The tornado diagrams for the three Scenarios show the impact on leakage of changing the parameters listed. The graphs show cumulative leakage of CO_2_ as a percentage of the total injected. Each parameter was assessed in turn by varying it between its maximum and minimum values, with all the other parameters held at their base case values. The parameters shown are those that have a significant impact on the computed results (a relative difference of at least 5% between the base case and sensitivity test results). Values listed are % leakage results at 10,000 years. Maximum and minimum values were defined as either two standard deviations from the mean, or: *used minimum and maximum values varying between 0 and 5 wells km^−2^; **minimum and maximum values as defined in the Methods Section ***Varied between 1 and 1.1 for the well-regulated scenarios and from 1.1 to 2.0 for the Poorly-Regulated Scenario

### Time averaged leak rates

The SSC results can also be expressed as time-averaged leakage rates, by dividing the cumulative leakage percentages by the model run time. This produces a single number as a percentage rate of leakage per year. This is an artificial and simplistic linear rate of leakage calculation, which obscures the complexity of varying leak rates through time (Fig. 4), but is nevertheless a useful tool for comparing our model results with leak rates deemed to be acceptable. These results, for the modelling years 1, 100, 1000 and 10,000 after the start of injection are shown in Table 2.

**Table 2:** Time averaged leakage rates of the three scenarios

Scenario	Time (year)	Time averaged leakage rate (% per year)^a^		
		P95	P50	P05
Offshore Well-Regulated	1	0.0005	0.0008	0.001
	100	0.0002	0.0004	0.0009
	1000	0.0001	0.0002	0.0006
	10,000	0.00005	0.00019	0.00063
Onshore Well-Regulated	1	0.001	0.002	0.005
	100	0.0007	0.002	0.004
	1000	0.0002	0.0009	0.003
	10,000	0.0002	0.0008	0.003
Onshore Poorly-Regulated	1	0.05^b^	0.2^b^	0.5^b^
	100	0.02^b^	0.06^b^	0.2^b^
	1000	0.002	0.008	0.02^b^
	10,000	0.0007	0.002	0.003

The leakage values are expressed as a percentage of originally injected CO_2_. Example times are presented at *t* = 1, 100, 1000 and 10,000 years

^a^Time averaged leak rates are calculated by dividing the total cumulative leakage computed for the selected model time by the same number of model years. This results in an artificial linear rate of leakage which is constant from the start of injection to the selected time, and obscures the true variation in leakage rates over time (cf. Fig. 4)

^b^Results that do not meet the 0.01% per year acceptability level^13,15^. Notably, even on this simple metric, all well-regulated regions pass the simple acceptability test at all timescales. For the worst-case Poorly-Regulated Onshore Scenario, time-averaged leak rates are unacceptable in the short term, but at least 95% of the realisations give acceptable time-averaged leak rates over long time scales (several 1000 years)

Our results highlight that annual leakage rates reduce over time and range from 0.00005 to 0.003% per year in the Offshore and Onshore Well-Regulated Scenarios, but reach up to 0.5% per year early in the worst-case Onshore Poorly-Regulated Scenario. These high CO_2_ leakage simulation results can be seen in the Onshore Poorly-Regulated histogram in Fig. 3b. Importantly, for both Well-Regulated Scenarios, time-averaged yearly leak rates are more than an order of magnitude less than 0.01% per year, the yearly leakage rate considered by many stakeholders to be acceptable for CO_2_ storage to remain effective as a climate mitigation tool^13,14^. For the worst-case, Onshore Poorly-Regulated Scenario, leakage is unacceptably high for the first 100 years, but will reduce to acceptable levels by 1000 years in at least half of cases. This provides confidence that even in a very pessimistic deployment scenario CO_2_ storage will provide a significant long-term climate benefit.

### Input parameter sensitivity analysis

We apply Monte Carlo analysis and sensitivity tests using the range of SSC input parameters to constrain their influence and identify the greatest sources of uncertainty in the results. The sensitivity analysis was undertaken on the input parameters for which reliable estimates of parameter variation were available (17/26 parameters - see Methods). Of these, only nine influenced the results to cause a deviation of greater than 5% (relative) from the base case value in at least one of the scenarios. These nine parameters are the focus of our sensitivity analysis discussion and are presented in Fig. 5; the parameters are detailed in the Methods section and rationale for the values chosen is provided in Supplementary Notes 2–7.

Residual trapping is a significant influence on total leakage results for scenarios that involve a high level of leakage. In the SSC, residual trapping (along with chemical trapping, inputs which were not varied in our sensitivity analysis due to using a single model – see Methods section) only directly limits leakage by immobilising CO_2_ and preventing it from leaving the reservoir. A lower level of residual trapping only causes a higher leakage result for scenarios where the simulation runs out of mobile CO_2_. This does not occur for any of the base case scenarios, and so the maximum leakage results that derive from minimising residual trapping are the same as the base cases.

A parameter that exerts a strong influence on all scenarios is the plume areal extent (Fig. 5). This is unsurprising as leakage via both abandoned wells and natural pathways are proportional to the plume area. However, our simplified model assumes that plume geometry will be controlled by well-defined traps, meaning migration and subsequent increased residual and solubility trapping will not take place. If storage was to be implemented in a horizontally-unconfined reservoir, as in the case of Sleipner^49^, then CO_2_ migration would be more pronounced, leading to higher levels of residual and solubility trapping^50,51^. This would result in the amount of CO_2_ available for leakage being lower and over time increased immobilisation due to plume spreading will outweigh the increased leakage risk posed by a larger plume area. These interactions between plume geometry, migration, and immobilisation are too complex to incorporate into our generalised model, but are likely to result in lower leakage than our model results.

The most influential parameters directly associated with CO_2_ leakage are the minimum long-term leakage rate (parameter A in the leakage reduction function), the long-term well-blowout frequency and mass lost per blowout, abandoned well density (frequency of wells per until of area), the abandoned well under-estimation factor, the proportion of degraded wells, and the natural leakage rate. Of these, the natural leakage rate is one of the least influential parameters, with leakage associated with abandoned wells having a stronger influence. Well density is solely responsible for the leakage increase between the Offshore and Onshore Well-Regulated Scenarios. For the Offshore Well-Regulated Scenario, which has a low abandoned well density, changing the well density produced the largest difference in leakage results, confirming that the SSC is highly sensitive to this parameter, likely due to the small amount of long-term leakage we assume for each well (see Supplementary Note 4). The minimum and maximum leakage results associated with varying the abandoned well density are similar for the Offshore, and Onshore Well-Regulated Scenarios, indicating that abandoned wells pose a significant leakage risk in both Well-Regulated scenarios, if the wells leak even a small amount due to cement corrosion over hundreds to thousands of years. In the Poorly-Regulated Scenario, the abandoned well under-estimation factor also imparts a noticeable influence on leakage results. Varying the under-estimation factor between 1.1 (where ~91% of abandoned wells are identified) and 2.0 (where only ~50% abandoned wells are identified) produces total leakage values of 3.76% and 18.74%, respectively.

The SSC considers leakage along abandoned wells in terms of continuous low-level leakage and discrete events (here called blowouts, irrespective of mass leaked). Varying the proportion of degraded wells, and thus the amount of continuous leakage imparts a minor influence on the SSC results and has the greatest impact in the Poorly-Regulated Scenario where degraded abandoned wells may not be identified and remediated. Variation of the low-level leak-rate experienced by intact wells was not modelled due to a lack of available data, but reducing this is expected to significantly enhance storage security. Leakage during a blowout exerts a moderate influence on the results, and is based on conservative estimates of leakage that do not account for improvements in well remediation techniques and technologies that are likely to occur with time and experience^52^. In most cases, the long-term blowout frequency imparts a greater influence on results than the amount leaked. The long-term blowout frequency is based on observations from steam injection fields in California where different inactive well blowout frequencies were determined for short-term, initial-defect controlled blowouts, and for longer-term, aging-related defect controlled blowouts^53^. However, no data yet exists for abandoned well blowouts from wells that are more than a few decades old. In the absence of more appropriate data we have extrapolated these long-term blowout frequencies for 10,000 years into the future, but acknowledge that these may not be wholly representative. While corrosion is considered to be a significant long-term risk to well integrity in CO_2_-rich reservoirs, other processes acting on the well may decrease permeability. Corrosion may be associated with carbonation and precipitation of minerals, effectively plugging defects^54^. Furthermore, stress regimes in many sedimentary basins promote closure of vertical pathways, with observations of reduction in annulus size and narrowing of steel casing in active wells over decades, suggesting that many abandoned well leakage pathways may become self-sealing over time^55,56^.

We attempted to incorporate the effects of these leak-inhibiting processes into the SSC via the leakage reduction function. This calculates a reduction in the leakage rate over time due to sealing of migration pathways, scavenging of leaked material into overlying reservoirs, and a loss of buoyancy as the plume stabilises and the amount of mobile CO_2_ is reduced by either immobilisation or leakage. This function was introduced to counter the lack of coupling between our surface flux leakage model and subsurface immobilisation model, and is based on observed and simulated leak rate decays over time. A parameter that defines this function is the minimum long-term leakage rate - the percentage of the initial yearly leakage rate that leakage will reduce to over time. This parameter exerts a strong influence on the results due to high uncertainty (minimum = 3%, maximum = 53%) and thus a large range in values. Applying an optimistic leakage reduction function to the SSC significantly reduces leakage, with the worst-case poorly-regulated scenario leaking just 7.8% of the injected CO_2_ (Fig. 5). The leakage reduction function is based on up to 500 years of leakage data, a timespan that is two orders of magnitude lower than we modelled, and thus carries considerable uncertainty. The minimum long-term leakage rate was introduced as a parameter to ensure that the SSC does not under-estimate long-term leakage by extrapolating rate decay trends far beyond their timeframes. However, given the above discussion on long-term blowout frequency, this approach may be overly conservative and CO_2_ leakage may be lower in reality than our model results suggest.

## Discussion

The input parameters we have applied to the SSC in this work were selected to be both as realistic as possible, based on the currently available data, but also conservative enough to ensure that we do not over-estimate storage security. Input parameters influencing post-injection abandoned well leak rates (both continuous leakage and blowouts) were especially selected to be conservative as large uncertainties remain in our knowledge of cement behaviour on the thousands-of-years timescale. In our simulations, natural leakage is also likely over-estimated. Our natural leakage input parameters are based on measured surface fluxes and represent mature leakage systems presumed to have reached a steady state. They do not account for the possibility of a time-lag between initiation of leakage from the reservoir, and the leaked material reaching the surface, as would be expected if the leakage pathway was connected to the overlying reservoirs^31^. The magnitude of this time-lag is highly uncertain; if it takes hundreds of years for the leaked material to reach the surface, then our simulations significantly over-estimate natural leakage. Furthermore, in reality, many cases of CO_2_ geological storage are expected to involve higher levels of immobilisation due to enhancement of residual trapping via migration^50,51^, and of solubility trapping via convective mixing as dense, CO_2_ saturated brine sinks^57^. These factors have not been incorporated into the SSC due to uncertainties in quantifying their contribution to CO_2_ immobilisation. Our model results are thus most-likely worst-case values for each scenario.

Even when applying these conservative input parameters, results from the SSC illustrate that CO_2_ storage in regions with moderate abandoned well densities and that are regulated using current best practice will retain 98% of the injected CO_2_ over 10,000 years in more than half of cases, and result in maximum leakage of 6.3% of the injected CO_2_ in fewer than 5% of cases. As expected, we find that unregulated storage is less secure. Here, however, over 10,000 years, only 22% of injected CO_2_ will leak in half of cases, with the possibility that up to 33% of the injected CO_2_ could leak in 5% of cases. This leakage is primarily through undetected and poorly abandoned legacy wells, and could be reduced through identification and remediation of leakage if a comprehensive site screening and monitoring program is deployed. Importantly, natural subsurface trapping mechanisms mean that this leakage will not continue indefinitely. Consequently, even with mitigation actions restricted solely to repair of abandoned wells that blow out, regions with a legacy of poorly regulated subsurface operations can reliably and robustly store and retain 78% of injected CO_2_. We find that regulators can most effectively improve CO_2_ storage security by identifying and monitoring abandoned wells, and perform reactive remediation should they leak.

Overall our findings indicate that geological storage of CO_2_ is a secure, resilient and feasible option for climate mitigation even when applying pessimistic values for input parameters and in poorly regulated storage scenarios. Hence, deployment of carbon capture and storage can be recommended to all governments as part of their actions to comply with the Paris 2015 target of keeping the global mean temperature rise well below 2 °C.

## Methods

### SSC program development and structure

**Fig. 6:**
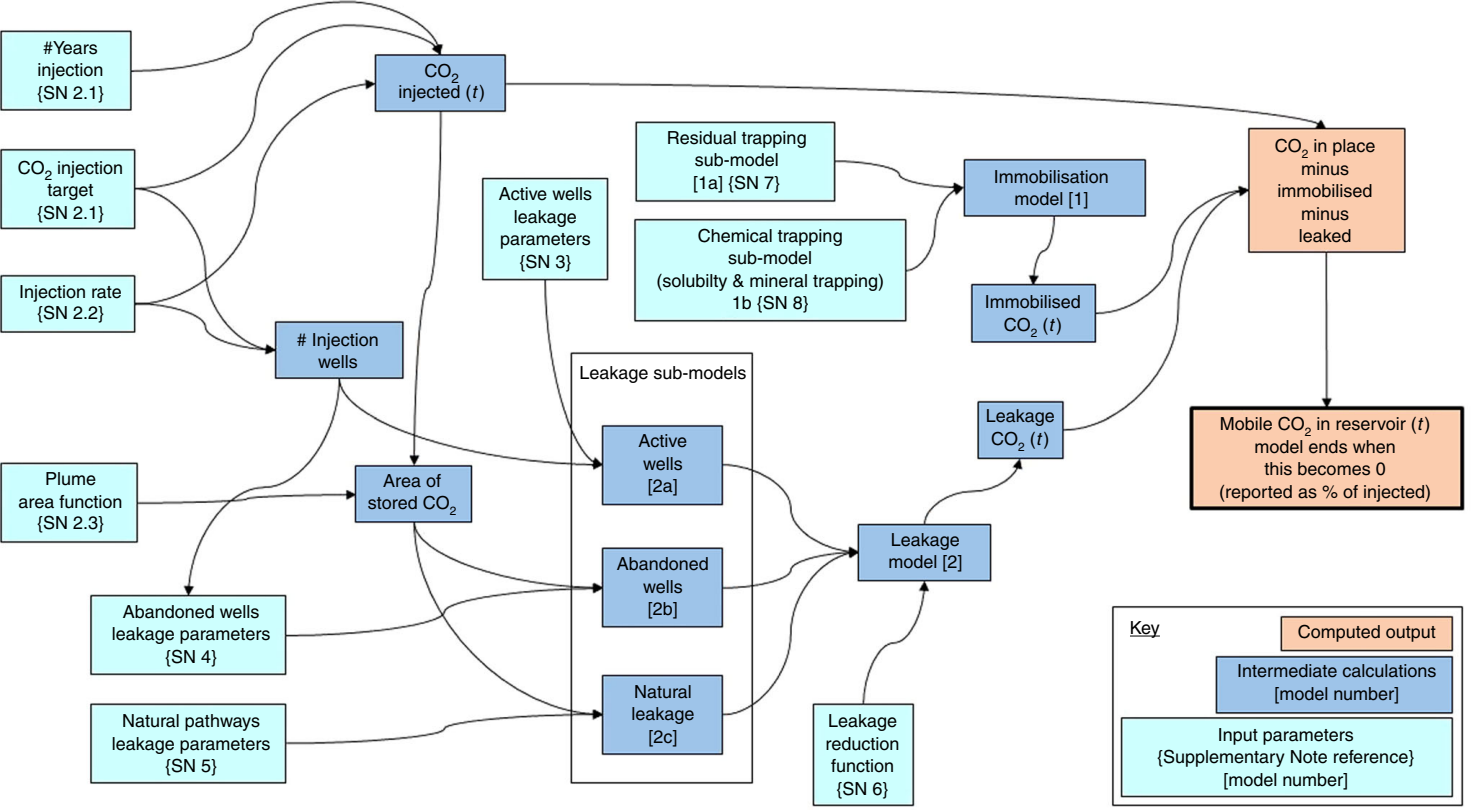
Schematic flow diagram of the Storage Security Calculator program. The program relies on 26 input parameters, each are called in at least one of the models. These parameters are listed in Table 3, along with the values applied. Discussion of how the values were derived is provided in the Supplementary Information Section 2

Factors influencing retention and leakage of CO_2_ in a subsurface reservoir were identified and a literature review carried out for each factor to determine the type of appropriate quantitative data available. The ‘Storage Security Calculator’ (SSC) structure was designed to make best use of the available data and resulted in the development of two separate models–a subsurface immobilisation and retention model, and a surface leakage model (Fig. 6). Each model assumes a common injection target and calculates the amount of CO_2_ leaked or immobilised for each year. The results of these models are summed for each year and subtracted from the total amount of CO_2_ injected to calculate the mobile CO_2_ remaining in the reservoir. If the amount of mobile CO_2_ in the reservoir reaches zero the model run ends, otherwise it continues until 10,000 years.

### CO_2_ injection

The model assumes a timeline with a 30-year injection period, during which the amount of CO_2_ injected into the reservoir increases from 0 to 100% (12 Gt). The increase in injected CO_2_ over the injection period is modelled as a linear increase.

### Immobilisation model

The immobilisation model (see [1] on Fig. 1) comprises two sub-models of residual [1a] and chemical trapping [1b], and are calculated based on the amount of CO_2_ injected into the reservoir. The residual trapping and chemical trapping are calculated using separate models, but these models interact as residually trapped CO_2_ is consumed by chemical trapping (solubility and mineral trapping). Computationally, this is achieved by calculating the amount of chemically trapped CO_2_, subtracting this from the total injected CO_2_ and calculating the amount of residually trapped CO_2_ from the remaining free-phase CO_2_.

The proportion of free-phase CO_2_ that becomes chemically trapped is calculated over time via:

1 $$\% \,{\mathrm{Solubility}}\,{\mathrm{trapping}}\,\left( t \right) = 0.204 \times t^{0.0342}$$

and

2 $${\% \,{\mathrm{Mineral}}\,{\mathrm{trapping}}\,\left ( t \right) = (1.67 \times 10^{ - 13} \times t^3) + (2.90 \times 10^{ - 9} \times t^2) + (1.40 \times 10^{ - 5} \times t)}$$

where *t* = time in years. These equations are derived from the Xu et al.^25^ model, as described in Supplementary Note 8.

The proportions are applied to the CO_2_ remaining in the reservoir (i.e., injected minus leaked) for a given year, to calculate the amount of CO_2_ chemically trapped in that year. The amount of residually trapped CO_2_ is derived by subtracting the amount of chemically trapped CO_2_ for a given year from the total injected CO_2_, and multiplying this by the fraction of CO_2_ residually trapped (Supplementary Note 7).

### Leakage model

The leakage model (see [2] on Fig. 1) combines three separate sub-models of active well leakage [2a], abandoned well leakage [2b], and natural pathways leakage [2c]. These models are combined to calculate maximum leakage rates for the injection and post-injection periods. The leakage model is mostly uncoupled from the amount of CO_2_ in the reservoir and is based on measured surface fluxes. During the injection period, we apply a linear increase from 0 to 100% of the calculated leakage rate, to mirror the increase in injected CO_2_.

Several elements in the leakage model require the number of injection wells, and the area of the injected CO_2_ plume. The number of injection wells are calculated by dividing the annual injection target (i.e., 12 Gt/30 years = 400 Mt per year) by the well injectivity (base case for all scenarios = 0.75 Mt CO_2_ per well per year). The areal extent of the CO_2_ plume is calculated by multiplying the injection target by the Plume Area parameter, which is an empirically derived ratio of area: mass (see Supplementary Note 2).

Two types of leakage are defined for active (injection) wells [2a]. The first is continuous leakage, which is calculated by multiplying the frequency of leaking wells (% of wells that leak) by the amount of CO_2_ leaked per year per leaking well (t CO_2_ per year). The second type of leakage is discrete events (blowouts) which are considered in terms of minor and major blowouts. Leakage from minor and major blowouts is calculated by multiplying the blowout frequency (events per well per year) by the mass leaked per blowout (t CO_2_ per event). The amount of CO_2_ leaked via continuous leakage, minor blowouts, and major blowouts are summed to give a maximum annual leakage from active wells. This leakage rate is only applied to the first 30 years–the injection period–of the model. More details are available in Supplementary Note 3.

Similar to active wells, leakage from abandoned wells [2b] is defined in terms of continuous leakage and blowouts. However, there are many more variables associated with abandoned wells, and thus calculating this leakage rate is more complex. A step-by step description of how abandoned well leakage is calculated is presented in Supplementary Note 9.

The number of abandoned wells are introduced into the model as areal densities (wells km^−2^), and leakage rates are initially calculated as leakage per km^2^ and finally multiplied by the plume area to calculate absolute leakage.

The true abandoned well density is calculated by multiplying the known well density by the under-estimation factor (=1 for well-regulated scenarios); from this the areal density of known and unknown wells are calculated. The areal densities of unplugged, plugged but degraded, and plugged and intact wells are then calculated by multiplying the well densities by the proportion (frequency) of each well status. We then assume that all known abandoned wells will be investigated and that known unplugged wells will be remediated and converted to plugged and intact wells prior to injection.

During the injection period, continuous leakage is calculated by multiplying the areal well density for each well status (i.e., plugged/unplugged, degraded/intact) by its associated leakage rate. Leakage via abandoned well blowouts is calculated by multiplying the short-term blowout frequency by the number of plugged wells, and the mass lost per blowout. Additionally, all unplugged wells (only present in Poorly-Regulated Scenarios where the well under-estimation factor >1) are assumed to blowout. Summing the masses lost per year gives the abandoned well leakage rate (t km^−2^ per year) AB1, applied for the injection period.

At the end of the injection period, all active wells are converted to plugged and intact wells. All unknown, unplugged wells experienced blowout and are assumed to have been identified and remediated to intact, plugged status. Other unknown, plugged wells that experienced blowout are assumed to have been degraded wells; these are thus identified, remediated, and converted to known and intact wells. We assume a comprehensive monitoring programme and that all known wells are monitored during the injection period, allowing identification and remediation of degraded wells and high continuous leak rates. Thus, at the end of the injection period, all known wells are converted to intact wells with a low leak rate.

Post injection, the continuous leak rate is again calculated by multiplying the areal well density for each well status by its corresponding leak rate. Leakage due to blowouts is calculated by multiplying the long-term blowout frequency (events per well per year) by the total well density and the mass leaked per blowout. Summing these masses lost per year gives an abandoned well leakage rate (t km^−2^ per year) AB2, which is applied to the post-injection period.

Abandoned well leakage rates are multiplied by the plume area to give mass lost per year. Definition of parameters are described in Supplementary Note 4.

The yearly amount of CO_2_ leakage along natural pathways [2c] is calculated by multiplying the CO_2_ plume area by the natural leakage rate parameter (in t km^−2^). The natural leakage rate parameter adopts gas flux data from regional and global scales. The minimum and maximum values are based on the estimate of global fluxes of geological CO_2_ (0.44 t km^−2^ per year), and the average areal flux from total petroleum systems (10 t km^−2^ per year), respectively. The most likely natural leakage rate assumed, 2 t km^−2^ per year, is based on the areal fluxes observed at the Ojai Valley natural seeps and data from the Rangely EOR field. More details are available in Supplementary Notes 2 and 5.

The Leakage model sums the leakage from natural pathways, active wells, and abandoned wells to give two leakage rates applied to the injection period and the post-injection period. These leakage rates are not calculated in a way that is coupled to the subsurface, but we expect changes in subsurface conditions, such as pressure and the amount of mobile CO_2_, to influence the amount of CO_2_ that can be leaked.

To address the increase in injected CO_2_ over the injection period, we invoke a linear increase in leakage rate from 0 to 100%, mirroring the simplified total injection rate. For leakage reduction once injection has ceased, we invoke a leakage decay curve that is based on empirical data (see Supplementary Note 6). This has the form:

3 $$\% \,{\mathrm{of}}\,{\mathrm{maximum}}\,{\mathrm{leak}}\,{\mathrm{rate}}\,{\mathrm{at}}\,{\mathrm{time}}\,t = A + \left( {100-A} \right) \times {\mathrm e}^{ - Bt}$$

where *A* and *B* are the iteratively derived input parameters for the leakage reduction function.

For a given year, the % of the maximum leak rate is calculated and multiplied by the post-injection total leak rate, to give the leak rate (t CO_2_ per year) for that year.

### Sensitivity analysis

In many cases, ranges of values are available for the model input parameters and we use these to carry out two types of sensitivity analysis: Monte Carlo analysis and input parameter tornado-diagrams.

To quantify the uncertainty on our model results derived from the uncertainty of the input parameters, we carry out a Monte Carlo analysis. For this analysis, we define ranges of values for each model parameter, using an appropriate probability distribution (normal, lognormal, triangular, or uniform–definitions of distributions are described in Table 3 and the Supplementary Notes 2–7). The SSC is run for 10,000 realisations, each selecting a random number for each model parameter from within the defined ranges. For selected years, we then compile the P05, P50 and P95 of the 10,000 realisations to obtain a range of leakage results that represent 90% confidence (i.e., range of results between P05 and P95).

**Table 3:** Input parameters for the model

Parameter	Offshore^a^	Onshore: WR^a^	Onshore: PR^a^
*General parameters*			
Injection target [CO2target]^b^	12 Gt by 2050		
Injection rate per well (t year^−1^) [injectperWell]	Normal distribution: mean = 0.75 × 10^6^ ± 0.083 × 10^6^; SE (400 wells) = 0.00415.		
Injection period [InjectionPeriod]	30 years		
Area:Mass ratio of CO_2_ plume [meanPlumeArea]	Lognormal distribution: mean of Ln = −0.7595 ± 0.8815; SE (25 data points) = 0.1763.		
*Active (Injection) well parameters*			
Fraction of injection wells that are leaking [ActiveWellFreq]	Distribution: lognormal; mean of Ln = −2.17 ± 0.6	Distribution: lognormal; mean of Ln = −2.89 ± 0.7	
Mass of CO_2_ leaked per leaking well year^−1^ [SlowLeakInjector]	Normal distribution: mean = 158.5 ± 18.83; SE (8 wells (1.3% of 400)) = 5.2		
Frequency of minor blowouts [MinorBlowFreq]	0.0693 events^−1^ well year^−1^		
Mass of CO_2_ lost per minor blowout (t) [MinorBlowout]	Distribution: log normal: mean of Ln(Ln) = 1.27 ± 0.21; SE (28 wells (400 × 0.0693)) = 0.0397.		
Frequency of major blowouts (events well^−1^ year^−1^) [MajorBlowFreq]	Distribution: normal; mean = 1.48 × 10^−4^ ± 3.33 × 10^−5^	Distribution: normal; mean= 1.35 × 10^−4^ ± 4.4 × 10^−5^	
Mass of CO_2_ lost per major blowout (t) [CO2MajorBlowout]	Distribution: lognormal: mean of Ln(Ln) = 2.57 ± 0.045; SE (17 data points) = 0.011		
*Abandoned well leakage parameters*			
Areal density of abandoned wells (wells km^−2^) [KnownWellDensity]	0.44	2.5	
Abandoned well under-estimation factor [wellUnderEst]	1	1	Uniform distribution 1.1 to 2.0 Base Case = 1.55
Fraction of abandoned wells that are unplugged [UnPlugWells]	0%	0%	0.3 (30%)
Fraction of plugged wells that are degraded [DegradWells]	Distribution: lognormal; mean of Ln = −2.17 ± 0.6	Distribution: lognormal; mean of Ln = −2.89 ± 0.7	
Fraction of intact plugged wells with the higher leak rate [IntactHighRate]	0.054 (5.4%)		
Plugged abandoned well blowout frequency for the first 30 years (events well^−1^ 30 years^−1^) [PlugBlowoutYear]	Distribution: lognormal; mean of Ln = −8.6125 ± 0.23 (1/2000 to 1/9000)		
Long-term blowout frequency (events well^−1^ year^−1^) [BlowoutWellYear]	Uniform distribution: 1 × 10^−5^ to 1 × 10^−4^; Base Case = 5 × 10^−4^ (1/50,000)		
Mass CO_2_ lost during an abandoned well blowout (t) [CO2largeBlowout]	Distribution: lognormal; mean of Ln = 13.4 ± 0.35		
Abandoned well continuous leak rates (t year^−1^) for:			
Degraded wells [CO2degraded]	300 t		
Intact wells with high leak rate [CO2intactHigh]	230 t		
Intact wells with low leak rate [CO2intactLow]	0.004 t		
*Other parameters*			
Leak rate via natural pathways (t year^−1^ km^−2^) [NatLeakRate]	Distribution: lognormal; mean of Ln = 0.693 ± 0.37		
% of CO_2_ residually trapped [res_sat]	Normal distribution: mean = 0.58 ± 0.1897; SE (44 data points) = 0.0286		
Long-term leakage reduction: % of maximum leakage rate at time t = A + 100 * e ^*(−Bt)*^			
Parameter A [A]	Triangle distribution: min = 3, max = 53, most likely = 12		
Parameter B [B]	Uniform distribution: 0.0143 to 0.5; Base Case = 0.257		

^a^Parameters are provided for three different scenarios (Offshore Well-Regulated, Onshore Well-Regulated, and Onshore Poorly-Regulated)

^b^Square brackets indicate the label assigned to the parameter in the R-code

To assess the sensitivity of our program to the different parameters, we define base-case, maximum, and minimum values, and run the program varying each parameter whilst holding the other parameters at their base case value (Fig. 5). In most cases, we only carried out sensitivity analysis on parameters for which ranges are defined by the literature review presented in Supplementary Notes 2–7.

For parameters that are defined by normal or lognormal distributions in the Methods Section, we defined the minimum and maximum values as ±2 standard deviations (note that this will produce a greater amount of variation than modelled in the Monte Carlo simulation, which varied some parameters using the standard error; as such, the sensitivity analysis is akin to applying the SSC to individual sites, rather than a global average). For other parameters we defined minimum and maximum values based on the range of data. Abandoned well areal density is defined as a single value for each Scenario, but we assessed the sensitivity of the models to this factor by varying between 0 and 5 wells km^−2^. This also allows us to consider the leakage risk for implementation of CO_2_ storage in regions without a legacy hydrocarbon industry. To investigate the impact of the well under-estimation factor in the sensitivity analysis we varied this between 1 (base case) and 1.1 for the well-regulated scenarios.

### R code

The R code is presented in a single file (SSC.R) which contains multiple sections and functions.

The base-case calculation is contained in the function SSCBase. This calls on a function called AbSetUp which calculates the AB1 and AB2 leakage rates (in t km^−2^ per year), based on the input parameters. The Monte Carlo analysis is carried out using the function SSCMC, which calls SSCBase and carries out n realisations (we choose 10,000) using randomly selected numbers from within the defined parameter ranges.

The code is organised as follows:

Section 1 includes functions that are not part of the base case or Monte Carlo calculations but are required to be loaded into the R environment to be called by SSCBase or SSCMC. Included here are the function AbSetUp (described above) and rtriangle^58^, which allows production of a random number from a triangle distribution defined by (minimum, maximum, most likely) values.

Section 2 contains the code for the function SSCBase. It creates a 2-dimensional matrix output, which consists of 10,000 rows (1 per year of the model) and 10 columns (1 = time (year); 2 = Injected CO_2_; 3 = CO_2_ leaked that year; 4 = Cumulatively leaked CO_2_; 5 = Leakage reduction parameter A; 6 = Leakage reduction parameter B; 7 = Mineral-trapped CO_2_; 8 = solubility trapped CO_2_; 9 = residually trapped CO_2_; 10 = remaining mobile CO_2_).

Section 3 contains the code for function SSCMC, which creates a list output, based on 10,000 iterations of SSCBase. To make the large amount of data generated more manageable, this function runs SSCBase using randomly selected numbers from the defined ranges, and then extracts the results for years 1, 3, 10, 30, 100, 500, 1000, 2000, 3000, 4000, 5000, 6000, 7000, 8000, 9000 and 10,000, storing the results for each year in a separate matrix. This process is repeated 10,000 times to create a list of 16 matrices, each containing 10,000 rows. The data is then interrogated using the FigLoss function described below.

Section 4 contains functions for interrogating the SSCBase and SSCMC outputs. The Basic function returns the total cumulative leakage, as a percentage of the total injected, at the end of the model run (maximum 10,000 years). MinMaxSA is code that allows minimum and maximum values to be substituted for the base case in SSCBase. The desired values are entered for the appropriate Min_/Max_ pair. For parameters where the minimum and maximum values are to be defined by standard deviations, the values can be entered as a mean plus or minus the standard deviation. Multiplying the standard deviation by SDN (standard deviation number) allows the minimum and maximum values to be quickly changed to check a different number of standard deviations. Code to plot the CO_2_ partitioning over time calls the results from SSCBase and plots parameters of interest against time. The function FigLoss converts the SSCMC cumulative CO_2_ leakage output to leakage as a percentage of the injection target and calculates the P5, P50 and P95 leakage percentiles for each year.

### Model availability

The R code for the Storage Security Calculator is available as a supplementary code file (Supplementary Data 1), using the Offshore Scenario as an example.

### Data availability

All data used to determine input parameters are summarised in Supplementary Notes 2–8, Supplementary Tables 1–10, and Supplementary Figs 1–13.

## Electronic supplementary material

Supplementary Information

Description of Additional Supplementary Files

Supplementary Data 1
