# Supplementary material for: Estimating geological CO2 storage security to deliver on climate mitigation
Source: Nat Commun. 2018 Jun 12;9:2201. doi: 10.1038/s41467-018-04423-1 (PMC5997736; doi:10.1038/s41467-018-04423-1)
Supplement: Supplementary file 1 — Supplementary Information [file 41467_2018_4423_MOESM1_ESM.pdf]

Supplementary Information for "Estimating geological CO_2 storage security to deliver on climate mitigation", by Alcalde, Flude et al. (2018)

Contents

Supplementary Note 1: Overview of the Storage Security Calculator (SSC) 3
Supplementary Note 2: General Parameter Definitions 5
2.1 Injection Targets 5
2.2 Injection rate and number of injection wells 5
2.3 Injection Plume Area 6
Supplementary Table 1: 7
Supplementary Figure 1: Area-to-mass ratios of natural gas fields 8
Supplementary Note 3: Leakage through Active (Injection) Wells 9
3.1 Background on well leakage 9
Supplementary Figure 2: Leakage pathways along wellbores 10
3.2 Continuous Leakage from active (injection) wells 11
Supplementary Figure 3: Leakage frequency for offshore injection wells. 12
Supplementary Figure 4: Leakage frequency for onshore injection wells 12
3.3 Leakage via discrete events from active wells (blowouts) 12
3.3.1 Sources of data 12
Supplementary Figure 5: Well blowout frequencies 14
Supplementary Figure 6: Blowout magnitude (mass lost) 14
3.3.2 Active well blowouts – Parameter definitions 15
Supplementary Figure 7: Minor blowout losses 16
Supplementary Figure 8: Major blowout losses 17
Supplementary Note 4: Estimating Leakage in Abandoned / Legacy Wells 18
4.1 Background and context 18
4.2 Sources of abandoned well data 18
Supplementary Figure 9: The relationship between permeability and leakage 20
4.3. General abandoned well parameters - well density and condition 20
4.4 Abandoned well continuous leakage parameters 21
4.5 Abandoned well discrete event parameters 21
Supplementary Table 2: General abandoned well input parameters 22
Supplementary Note 5: Natural Leakage 23
Supplementary Figure 10: Natural leakage fluxes 25
Supplementary Note 6: Reduction of leakage rate with time 26
Supplementary Figure 11: Leakage reduction 28
Supplementary Note 7: Residual Saturation Trapping 29
Supplementary Figure 12: Residual Trapping 29

Supplementary Note 8: Chemical Trapping 30
Supplementary Note 9. The Leakage Model (2) 31
9.1. Active Well Leakage (2a) 31
9.2 Abandoned Well Leakage (2b) 31
9.3 Natural Leakage (2c) 33
9.4 Combined Leakage Model (2) 33
Supplementary Note 10: The immobilisation models (1) 35
10.1 Residual Trapping (1a) 35
10.2 Chemical Trapping (1b) 35
Supplementary Note 11: The integrated model (1) 36
Supplementary Note 12: The R-code for the Storage Security Calculator 37
Supplementary Table 3 38
Supplementary Table 4 39
Supplementary Table 5 41
Supplementary Table 6 42
Supplementary Figure 13: Blowout frequencies 44
Supplementary Table 7 45
Supplementary Table 8 48
Supplementary Table 9 50
Supplementary Table 10 51
Supplementary References 52

Supplementary Note 1: Overview of the Storage Security Calculator (SSC)

This Supplementary Information document describes how we derived the model inputs (Supplementary notes 2 to 9) along with the rationale for the values selected, and the structure of the computational program. A summary of the inputs is provided in the methods section of the main manuscript. Details of the sub-models and of the integrated model (Supplementary Notes 10-12) are also described to aid the reader in understanding the program.

This document is designed to be used as a reference text rather than read cover-to-cover, and thus involves a certain amount of repetition of information, in order to be user-friendly. Figure 6 of the main text shows how the different sections of the program interact, along with the sections of this document that discuss the relevant input parameters.

The SSC combines an immobilisation model (1), comprised of residual trapping (1a) and chemical trapping (1b) sub-models; and a leakage model (2), comprised of active well (2a), abandoned well (2b) and natural pathways (2c) leakage sub-models.

Three storage environment scenarios are explored by the SSC:

1) Scenario A: “Offshore Well-Regulated” - Storage in an offshore environment that takes advantage of a mature, well-regulated hydrocarbon industry in the region.

2) Scenario B: “Onshore Well-Regulated” - Storage in an onshore environment that takes advantage of a mature, well-regulated hydrocarbon industry in the region.

3) Scenario C: “Onshore Poorly-Regulated” - Storage in an onshore environment that has experienced an extensive, but historically poorly-regulated hydrocarbon industry in the region.

The required information to characterise these scenarios was obtained from certain geographical regions that could be described as examples of these scenarios. These include the North Sea (Scenario A), Texas, USA (Scenario B), and Pennsylvania, USA (Scenario C). Scenarios A and B are the most likely targets for CO_2 storage sites, providing a balance of the benefits of existing knowledge with the increased potential risk of leakage pathways due to legacy wellbores; but we include Scenario C as a worst-case scenario to investigate CO_2 storage security in the event of sub-optimal regulation.

In Section 2 we discuss how values were derived for each model input parameter. Each parameter has a maximum and minimum value and a base case value, which we consider to be a reasonable and likely, albeit often conservative estimate. These parameters are used to calculate the base case scenarios. For a sensitivity analysis we employ a Monte Carlo method to select random values for each parameter, from within a defined range of values or distribution, and run the model 10,000 times. These ranges and distributions are based on real data, where possible, and are described on a case-by-case basis in Section 2.

For parameters that describe average values (e.g. the amount of CO_2 leaked per well), the Monte Carlo analysis picks a random number from a distribution defined by the mean and the standard error (=σ/sqrt(n), where "n" is defined either as the number of observations in the data set that the distribution is based on, or as the minimum number of virtual samples that will be returned by the model). This ensures that the random values are statistically representative and do not return an extreme value with very low probability. For example, continuous leakage in active wells is expected to result in loss of between 102 and 215 t of CO_2 per year from each leaking well. Continuous leakage is calculated from the frequency (i.e. % of wells that leak) and the average amount of CO_2 leaked per leaking well. Random sampling of a normal distribution that fits the minimum and maximum values for amount leaked per leaking well, defined by a mean and standard deviation, would produce leakage values equivalent to a single well, rather than an average of multiple wells, and would result in over-representation of extreme values. Random

selection of the high value (215 t CO_2 year^{-1}) would imply that every single leaking well leaks the maximum amount of CO_2, which is very unlikely. To avoid this, where parameters are average values, we define the distribution based on the mean and the standard error, instead of the mean and standard deviation.

Supplementary Note 2: General Parameter Definitions

General parameters comprise our basic assumptions, including injection targets, injection rates, and the areal extent of the subsurface CO_2 plume. These are parameters that influence all three models.

2.1 Injection Targets

In 2013, the IEA published estimates of the amount of CO_2 required to be stored by 2050, to meet the IEA's 2 °C (2DS) scenario, in which there is an 80% chance of limiting average global temperature increase to 2 °C [1]. Regional 2050 storage targets range from 3.5 Gt for non-OECD Latin America, up to 42.2 Gt for China, with a total of over 120 Gt CO stored globally by 2050.

Here, we apply the SSC to regional scenarios and we select a 2050 storage target of 12 Gt CO_2, in keeping with the storage target of OECD Europe. However, we note that the nature of the calculations employed by the SSC scales the leakage impact according to the amount of CO_2 injected, and so changing the storage target makes no difference to the proportion of CO_2 leaked. To achieve this target, we make a simplistic assumption that full CCS chain will be in place and operational by 2020 and that injection will take place over 30 years, from 2020 to 2050. Injection is assumed to take place gradually; programmatically this involves addition of 1/30 of the CO_2 target per year (i.e., 400 Mt year^{-1}) to the reservoir.

2.2 Injection rate and number of injection wells

Assuming that adequate storage capacity is available and that the injection targets will be met, the number of injection wells needed will depend on the injectivity of each well.

There are three main sources of data regarding CO_2 injectivity: enhanced oil recovery with CO_2 (CO_2-EOR), pilot CO_2 storage projects and commercial CO_2 storage projects. Pilot storage projects often inject small volumes, verifying injectivity before scaling up to larger projects. As such, their associated injection rates are lower than those likely to be applied at commercial storage sites; therefore, we do not consider pilot CO_2 storage site injection rates to be representative of a CCS industry implemented at a full, global-scale. At commercial CO_2-EOR injection sites, large tonnages of CO_2 may be injected, but these are often spread over a large number of wells for operational reasons and so do not necessarily represent optimised single-well injection rates. For instance, initial injection rates at Weyburn were up to 5000 t of CO_2 per day, equivalent to 1.8 Mtpa [2], but this was spread over nine injection wells[2] to optimise CO_2 contact with the residual oil. Hence, these existing injections are likely to be lower than those used in commercial storage sites.

Our injection rate parameter is based on measured CO_2 injection rates from commercial-scale storage projects. CO_2 injection rates are available for six commercial scale CO_2 storage sites (Supplementary Table 2) and range from 0.2 to 1.1 Mtpa. We assume that CO_2 storage sites will be designed to optimise injectivity, and we adopt a range of injection rates from 0.5 to 1 Mtpa, with a base case value of 0.75 Mtpa.

The number of injection wells needed to achieve the 12 Gt injection target is calculated based on the storage target, the injection period, and the injectivity. Our injection target is a constant 12 Gt CO_2 over 30 years. This equates to 400 Mt CO_2 per year, which gives a base case scenario requirement of 533.3 injection wells.

For the sensitivity analysis, we assume that well injectivity can be described by a normal distribution with a mean of 0.75 Mtpa (the base case value) and where the maximum (1 Mtpa) and minimum (0.5 Mtpa) values represent 3 standard deviations from the mean. This gives a standard deviation of 0.083.

The injection rate parameter is used to calculate an average injectivity value for hundreds of wells, and so we use the mean and standard error to create a distribution for the Monte Carlo analysis. To ensure we are not under-estimating the errors, we assume the smallest value of n that will be sampled by our program. In this case, n represents by the number of injection wells, which is defined by the injectivity. The highest injectivity (1 Mtpa) will result in the smallest number of wells, giving n=400. The standard error is thus 5.21×10^{-7}.

2.3 Injection Plume Area

Within the SSC program, the areal extent of the injection plume has a significant impact on leakage from abandoned wells, and via natural pathways, because the plume area will determine how many potentially leaking structures (e.g. abandoned wells or open faults) will be contacted by the injected CO_2. Plume area will depend on the geometry of structural traps and the mass of CO_2 injected. There are not yet enough large-scale CO_2 storage projects to assess the likely area impacted by a CO_2 plume during CO_2 storage, and so we use natural gas fields as an analogy. We use the data listed in Appendix 1 of Gluyas and Hitchens[3] and concentrate on fields for which area, recoverable gas volume, and gas expansion factor data are available. We use the recoverable gas and the gas expansion factor data to calculate the volume of gas in each reservoir. We then assume an in-reservoir CO_2 density of 700 kg m^{-3} to calculate the equivalent mass of CO_2 for each field. We then divide the field area by the mass of CO_2 to obtain an area-to-mass ratio. Data for individual fields are displayed in Supplementary Table 3. A histogram of the area-to-mass ratio values is displayed in Supplementary Figure 1. The data do exhibit a normal distribution, but can be interpreted as an incomplete lognormal distribution where the natural logs of the data have a mean of -0.7595 ± 0.8815 (one standard deviation), based on 25 data points. This gives a standard error on the mean of the logged data of 0.1763. We thus use this mean (i.e. $e^{-0.7595}$ km^2 / Mt) as the base case, and use the standard error (i.e. $e^{0.1763}$) for sensitivity analysis.

Supplementary Table 1:

Measured and modelled CO_2 injection rates per well.

Ref.	Site	Purpose	Quoted rate	Normalised rate
4	Zama Field	Acid Gas (CO_2 and H_2S) EOR and storage pilot.	63700 t CO2 injected Oct 2006 - Jan 2011	0.0147 Mt $year^{-1}$
5	Citronelle Oil Field	Demonstration-scale storage	Class V well approved a max injection rate of 182,500 metric tonnes CO_2 per year.	0.1825 Mt $year^{-1}$
6	Cranfield	CO_2-EOR pilot	1.2 Mtpa via 23 wells	0.05 Mt $year^{-1}$ $well^{-1}$
5	Illinois Basin - Decatur Project	Pilot-scale storage	1,000 metric tonne per day target	0.365 Mt $year^{-1}$
5	Michigan Basin Project	Pilot-scale storage	Max 1000 tpd	0.365 Mtpa
2	Weyburn	CO_2-EOR	5000 t per day over 9 wells	0.2 Mt $year^{-1}$ $well^{-1}$
7	Sleipner	Commercial-scale storage	12 Mt over 14 years	0.857 Mt $year^{-1}$
7	Snøhvit.	Commercial-scale storage	0.8 Mt injected from April 2008 – September 2010	0.331 Mtpa
8	Aquistore	Commercial-scale storage	Sustained injection rates of 400-600 t per day	0.2 Mt $year^{-1}$
9	Quest	Commercial-scale storage	1 Mt injected in the first year using 2 of the 3 available wells	0.5 Mt $year^{-1}$ $well^{-1}$
10,11	Illinois Industrial CCS	Commercial-scale storage	Project aims to inject 1.1 Mtpa. 46,300 t injected from April 7th 2017-end April 2017. This is equivalent to 0.73Mtpa	1.1 Mt $year^{-1}$
12	In Salah	Commercial-scale storage	Injection commenced in 2004 and since then over 3.8 million tonnes of CO_2 have been stored; decision to suspend CO_2 injection in June 2011	0.543 Mt $year^{-1}$

Supplementary Figure 1: Area-to-mass ratios of natural gas fields

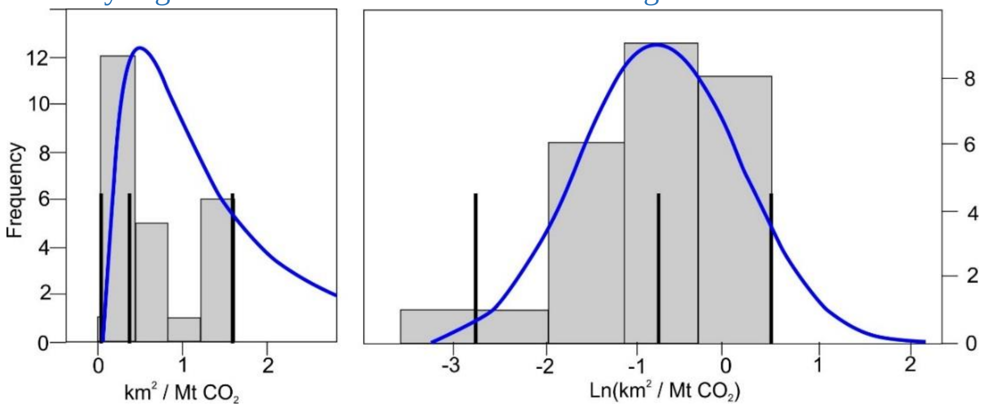

Histograms of area-to-mass ratios of natural gas fields (grey boxes) and fitted lognormal distributions (blue lines). Black vertical lines represent the minimum, mean, and maximum values.

Supplementary Note 3: Leakage through Active (Injection) Wells

3.1 Background on well leakage

Wells, both active and abandoned, present pathways for the leakage of CO_2 from geological storage. Wells developed for production have a steel casing inserted which is sealed in place with cement [13] (Supplementary Figure 2), while wells abandoned at the exploration stage consist of a simple well bore through rock and may or may not be sealed by a series of cement plugs. Well blowouts are rare but significant events that transfer volumes of fluids from geological depth to the surface, and can occur through both active (injection and / or production) and abandoned wells. Less dramatic, low-seepage rate leaks associated with wells may also occur. Numerous potential leakage pathways exist for wells[14,15] (Supplementary Figure 2), although modern well design incorporates numerous blowout prevention mechanisms to mitigate and control any unplanned fluid flow[16]. In the case of CO_2 storage, considerable attention has been paid to long-term well integrity as CO_2-associated corrosion of steel and cement casing has been cited as a cause of blowouts and well failure[17,18].

However, laboratory studies of well casing materials and samples taken from decades-old CO_2 injection wells suggest that corrosion may precipitate as well as erode material, and does not always lead to enhanced permeability[19–30]. Steel corrosion can be minimised in newly commissioned wells by the use of corrosion resistant carbon steel and by good cementing practice[23], but corrosion may present a significant leakage risk that increases over time in pre-existing wells. When present, cement corrosion most often exploits pre-existing cracks and defects, thus modifying pre-existing pathways such as a poor seal between the wellbore wall-rock and the casing cement[20] (potential leakage pathway #3 in Supplementary Figure 2). The risk of poor cement seals can be minimised by good, modern industrial practice.

We assume that any wells drilled for the application of CCS will employ best-practice and operate in a well-regulated industry and hence above average failure rates are not expected. We also assume that best practice will involve installation of blowout prevention and control equipment and training for well operation personnel[18]. Pre-existing legacy (presumed abandoned, for simplicity) wells within a CCS site may have been drilled, completed, and abandoned without adequate regulation and using materials less suitable for CO_2-bearing fluids. Hence, these wells are expected to have a greater than average risk of leakage, that will vary by geographical region (the different levels of leakage risk associated with regions with differing industrial histories are assessed by comparing our Scenarios 2 and 3). However, we also assume that CO_2 storage site operators will have a duty to monitor and remediate any leakage on pre-existing wells and that any significant leakage will be mitigated on timescales typical of dealing with an active well blowout.

We have reviewed the scientific and agency literature and databases for frequency of leakage and for leakage rates or total amount of material leaked to estimate expected leakage from active and abandoned wells. Leakage frequency is reported in the literature as a range of units and scales, including incident rates within an entire industry, incidents or leakage from a single site or field, as incidents per well or as wells per site. Industry-wide and site-scale data are reviewed for context, but such data are only included in our calculations where it is possible to convert the data to incidents per well per year for discrete events, or proportion of leaking wells for continuous leakage. When estimating the amount of CO_2 expected to leak, data for gas leakages (instead of leakage of other fluids) are considered the most appropriate. The amount of material leaked is collected as a volume value where available, or converted to a volume assuming standard

temperature and pressure ("STP" – 0°C, 1 atm), if appropriate. We report volumes in the units quoted in the source publication and convert these to cubic meters (m^3). These volumes are then converted to an equivalent mass of CO_2 (tonnes, t, or megatonnes, Mt, as appropriate) assuming a pure, ideal gas at standard temperature and pressure. This approach (calculating equivalent mass of CO_2) differs from other similar studies such as that of Loizzo et al[31] who convert reported leakage volumes to masses of the gas leaked in the case study (e.g. CH_4).

The data used to derive leakage estimates for these wells was extracted from a range of sources. The data from the underground gas storage (UGS) industry are the most pertinent as they describe scenarios of injecting gas into geological reservoirs for storage. Unconventional hydrocarbon production (hydraulic fracturing/EOR) often involves injection of fluids (including CO_2) into the reservoir and is also relevant, but the available well-failure data of hydrocarbon production often does not distinguish between conventional and unconventional operation wells. Furthermore, data relating to blowout or leakage frequency do not always distinguish between gas and oil production.

In terms of leakage from wells, two modes of leakage can be distinguished: 1) continuous, low level leakage of gas that is not considered to pose an acute hazard to operational staff or the environment; such leaks are monitored but, in traditional hydrocarbon industry practice, may not be remediated until the well is abandoned; 2) acute events involving unplanned and uncontrolled release of fluid (gas / oil / water) from the well (blowouts) that require rapid assessment and remediation as soon as possible. These two leakage modes will be reviewed separately.

Supplementary Figure 2: Leakage pathways along wellbores

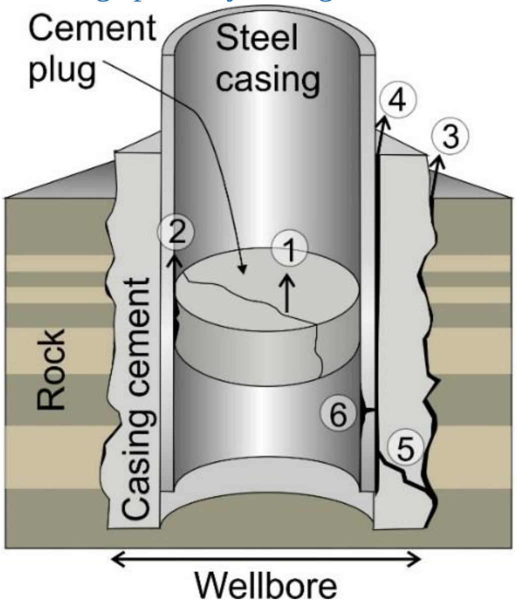

Leakage pathways along plugged and abandoned wellbores. 1: Corrosion or fracture of the in-well cement plug. 2: Poor contact between the stainless steel well casing and the cement plug. 3: Poor contact between the wall rock of the well bore and the casing cement. 4: Poor contact between the casing cement and the stainless steel casing. 4: Fracture or corrosion of casing cement. 6: Corrosion of the stainless steel well casing. Leakage pathways for active wells are similar and include pathways 3-6 with additional possibility of unintended gas flow back up the wellbore (blowout) during operation. Modified from [32].

3.2 Continuous Leakage from active (injection) wells

Minor continuous leakage events are considered to occur at wells that develop sustained casing pressure (SCP), sustained casing vent flow (SCVF) or small leaks, where the flow rate is low enough to not require remediation. Bachu and Watson[33] suggest that gas leaking during SCP is derived from shallower horizons than the target injection reservoirs, and so such leakage rates may not be relevant for our calculations. However, deep sources of gas have been identified as the source of SCP in some cases[34]. A reasonable amount of information regarding slow leakage of hydrocarbon production wells is available, but less so for injection wells. Bachu and Watson[33] noted the occurrence of SCVF on CO_2 injectors in Alberta, but these were documented as discrete events, rather than continuous leakage, and were quickly remediated. Rather than assume that an absence of evidence is evidence of absence, we review the frequency and level of continuous leakage in hydrocarbon production wells and incorporate this data into our models. This allows us to assess the impact of slow leaks from injection wells on overall CO_2 storage security. Supplementary Table 4 lists data on hydrocarbon wells that have reported leaks, casing failures, or SCP, in nineteen different studies, for onshore and offshore fields, along with the minimum, maximum and average proportion of wells that are leaking. The data suggest that offshore wells have a higher leakage risk (see histogram in Supplementary Figure 13). This is unsurprising given the added difficulties of cementing wells in offshore environments compared to onshore. We thus select different leakage frequencies for onshore and offshore environments.

Offshore well leakage frequencies range from 0.02 (2%) to 0.6 (60%), with a mean of 0.145 (14.5 %). However, these data do not form a normal distribution (Supplementary Figure 3), and are better described by a lognormal distribution with a mean of the natural logs of -2.17 ± 0.6 (one standard deviation). This distribution produces a reasonable fit for both the logged and original values (Supplementary Figure 3). We use this lognormal mean (i.e. $e^{-2.17}$) as the base case value.

Onshore well leakage frequencies range from 0.013 (1.3%) to 0.22 (22%), with a mean of 0.075 (7.5%). As for the offshore wells, we describe the data as a lognormal distribution with a mean of the natural logs of -2.89 ± 0.7 (one standard deviation). This distribution produces a reasonable fit for both the logged and original values (Supplementary Figure 4), and we use the mean (i.e. $e^{-2.89}$) as the base case value.

A comprehensive survey on gas leakage through well cement documented a range of leak rates from < 5000 cf (142 m^3) to > 250,000 cf (7080 m^3) per day[35], while other documented leak rates associated with non-hazardous SCP are on the order of 142 m^3 (5000 cf)[19] to 200 m^3 [36] per day (see Supplementary Table 5). The lower estimate seems to be ~ 5000 cf per day, which equates to 102 t CO_2 $year^{-1}$. The upper limit for allowable leakage without the need for well reparation in Alberta is 300 m^3 per day, which equates to 215 t CO_2 $year^{-1}$. The mid-point of these two values is 158.5 t CO_2 $year^{-1}$, which we take as the base case.

For the sensitivity analysis, we assume a normal distribution with a mean of 158.5 t CO_2 $year^{-1}$ (the base case value) and where the maximum (102 t CO_2 $year^{-1}$) and minimum (215 t CO_2 $year^{-1}$) values represent three standard deviations from the mean. This gives a standard deviation of 18.83. To calculate the standard error, we calculate the lowest number of wells likely to be leaking by multiplying the lowest leak frequency (1.3% - onshore leakage) by the smallest number of injection wells (400 wells at the maximum 1 Mt $year^{-1}$ injection rate) to give a standard error of 5.2.

Supplementary Figure 3: Leakage frequency for offshore injection wells.

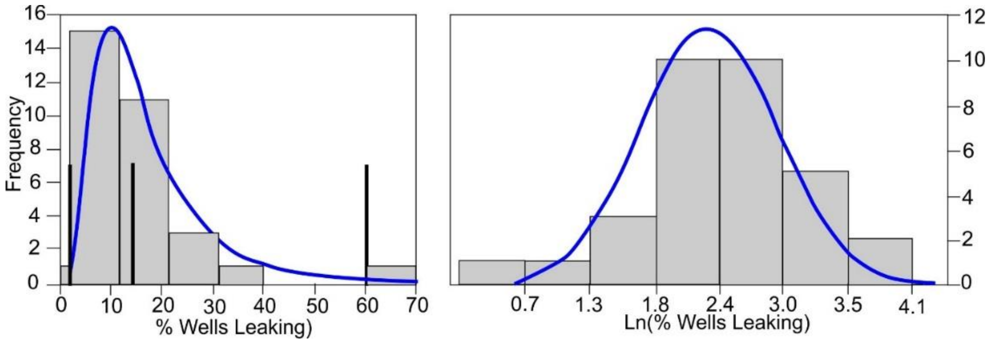

Offshore injection well leakage frequency. Histograms (grey boxes) and fitted lognormal distributions (blue lines) for the percentage of injection wells that exhibit continuous leakage. Data from Supplementary Table 4).

Supplementary Figure 4: Leakage frequency for onshore injection wells

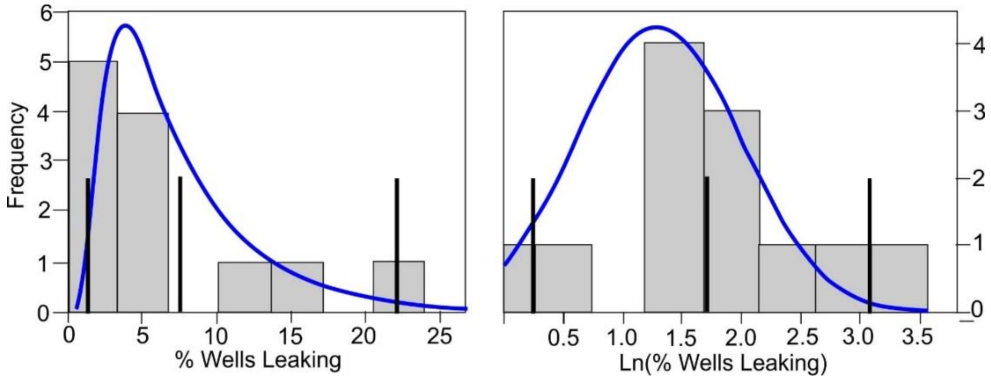

Onshore injection well leakage frequency. Histograms (grey boxes) and fitted lognormal distributions (blue lines) for the percentage of injection wells that exhibit continuous leakage. Data from Supplementary Table 4.

3.3 Leakage via discrete events from active wells (blowouts)

3.3.1 Sources of data

Uncontrolled release of fluids (blowouts) from a well are rare but ubiquitous events in the hydrocarbon industry. Many blowout incidents are associated with exploratory drilling and well completion[16]; the frequency of blowouts associated with exploratory and developmental drilling is up to two orders of magnitude greater than for production wells[37] due to the risk of drilling into an unexpectedly over-pressured reservoir. In the case of CO_2 storage, drilling related blowouts will occur prior to the CO_2 injection and thus will not result in leakage of the stored CO_2 back to the surface, hence we focus on production and injection blowout rates, where available.

Several studies have collated data on well failure and pollution incidents associated with hydrocarbon wells, but few distinguish between well operation phase and not all sources distinguish between pollution incidents associated with well failure and those associated with surface processes such as improper waste disposal. Hence, many quoted data (Supplementary Table 6) are likely to be over-estimates of the expected incident frequency. Closer inspection of the information sources cited by many well failure review papers[16,38] shows that, in some cases,

overly-simplified data has been used to calculate failure rates. For example, frequency of documented pollution incidents is often used as a proxy for well failure, but leakage of fluids from depth only accounts for a minority of incidents in such cases. A further complication is the imprecision of terminology used to describe subsurface fluid releases. Terms such as "blowout", "well failure", "well / fluid release", "loss of well control" and "leak" may all be used to describe an uncontrolled release of fluids from the subsurface, but many of these terms can also describe "near misses"[39,40]. For example, some studies consider a "blowout" and a "well release" to be different types of events, the latter being an unintended flow of fluid from the well that was stopped by the well barrier system while "blowout" is reserved for complete failure of the well barrier system[39,41]. The term "leak" may refer to a discrete incident, or to continuous leakage, as described in the previous section. Another problem is the imprecise definition of incident frequency in the literature. For instance, when quoting frequencies as percentages, it is rarely clear whether percentage of wells experiencing failure is being discussed, or it refers to the percentage of well-years that a failure occurs in. Collating and comparing discrete leakage event (here referred to as "blowouts", even if minor, and including both surface and subsurface blowouts) frequency data is thus not straightforward. Published and calculated blowout frequencies are listed in Supplementary Table 6 and relevant examples are discussed below. Supplementary Table 7 lists examples of blowouts along with amount of material leaked and blowout duration, where available. Case studies where high well failure rates are due to production from poorly consolidated reservoirs (e.g. Malacca Straight, Indonesia[42]) are not included in our analysis.

Where available, plotting blowout frequencies against the number of wells in each case study (Supplementary Figure 5) shows that blowout frequency tends to decrease with increasing study size, suggesting that smaller studies are unlikely to be representative of the whole industry.

The highest documented blowout frequency (0.0693 releases per gas well year^{-1}) comes from analysis of the UK Health and Safety Executive (HSE) North Sea population[43] and hydrocarbon release databases[44]. Wellhead Process release incidents were selected to exclude non-blowout incidents (such as surface spills or storage container leaks), but it is possible that this high blowout rate is an over-estimation due to not fully excluding other release sources and mechanisms. The durations of the selected incidents ranged from one minute to 70 days, with an average of ~3.5 hours. Total volumes of gas released range from 9.80 x 10^{-5} m^3 (1.9 x 10^{-7} t CO_2 equivalent) to 1.98 x 10^{6} m^3 (3889 t CO_2 equivalent), with an average of 1.93 x 10^{3} m^3 (3.8 t CO_2 equivalent). Only two of the 1,597 gas release incidents were of a volume greater than 2.55 x 10^{5} m^3 (500 t CO_2 equivalent). The vast majority of these incidents are thus volumetrically small and it is possible that the difference in blowout frequency between this database and other North Sea databases[39] reflects a difference in reporting standards and/or how a blowout or well release is defined. However, other offshore production blowout frequencies suggest that there is a greater risk of blowout for offshore wells than for onshore wells (Supplementary Figure 5).

Few data exist for CO_2 injection well failure frequencies, and those available are on small data sets. One study, summarising events in acid (H_2S) and CO_2 injection wells in Alberta, Canada[33], discusses the difference in blowout rates between injection and production wells. Here, well failure events were found to be due to general well operation issues and were not caused by injection[33]. They found a greater risk of well failure in wells converted from production to injection wells, rather than wells initially completed for injection, and in wells drilled before improved regulations came into force in 1994. On the other hand, well integrity failures were found to be twice as common in injection wells as in production wells on the Norwegian Continental Shelf (17% of 526 production wells vs 29% of 185 injection wells experienced well integrity issues in 2007)[45], although these did not necessarily result in leakage.

The next most relevant case studies are those of UGS experiences, where the gas is stored in saline aquifers or depleted hydrocarbon fields. Blowout frequencies are quoted from a number of studies

addressing the worldwide UGS industry. Well numbers are generally not available, but these values are expected to be representative of the industry. These values fall well within the range of blowout frequencies for hydrocarbon production (Supplementary Figure 5).

The volume of material lost during a blowout is reported for a number of case studies, but no studies currently exist that consider the relative frequencies of different blowout magnitudes. One study[31] estimates frequency of blowouts of varying severity but bases severity on the environmental and health and safety impact of the incident, which is not necessarily proportional to mass of material (e.g. CO_2) lost. Supplementary Table 7 summarises leakage details from a selection of blowouts. Mass lost during a blowout has a positively skewed distribution (Supplementary Figures 7 and 8) with most of the blowouts releasing less than 10 t CO_2 equivalent. Supplementary Figure 6 combines the blowouts from Supplementary Table 7 with the HSE blowout data[44] and shows that the vast majority of blowouts release less than 500 t CO_2 equivalent.

Supplementary Figure 5: Well blowout frequencies

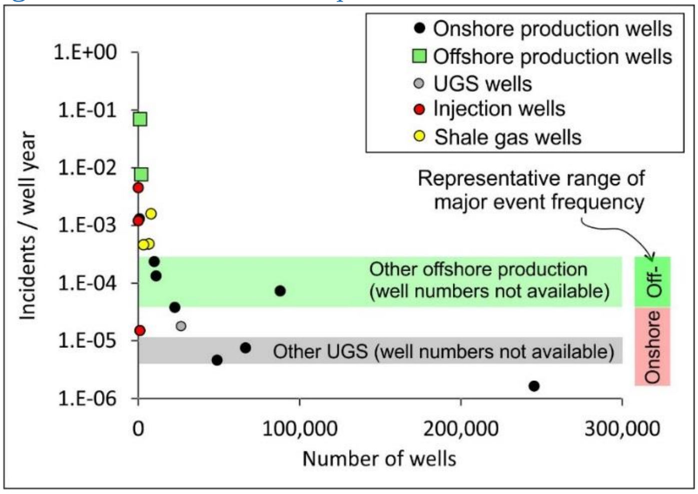

Figure 5: Blowout frequencies plotted against the number of wells involved in each case study. Where absolute well numbers are not available, they are estimated by dividing well years by the number of years of study. Some case studies for offshore and UGS blowouts do not provide number of wells or number of years – the blowout frequencies for these are represented as horizontal bands on the chart. Data from Supplementary Table 6.

Supplementary Figure 6: Blowout magnitude (mass lost)

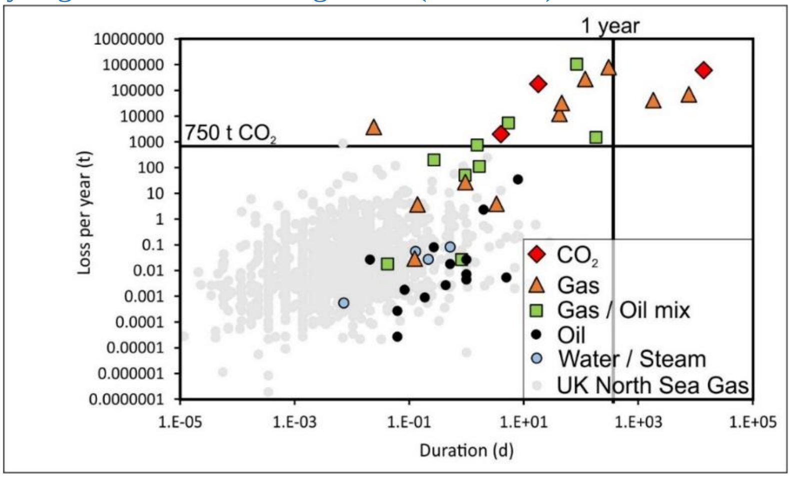

The magnitude of blowout release (as volume equivalent mass of CO_2 leaked in a single year – for blowouts lasting less than a year this is the total leaked) plotted against blowout duration.

3.3.2 Active well blowouts – Parameter definitions

Given the above discussion, we consider blowout frequencies in terms of volumetrically minor and major incidents. Minor blowouts are based on the UK North Sea HSE database with a blowout frequency of 0.0693 events per well per year and are considered to leak between 1 and 500 t CO_2 equivalent per event. Blowout data are mostly from conventional hydrocarbon wells. Remediation of a CO_2 blowout may take longer and/or emit proportionally more gas due to complications resulting from CO_2 flow (rapid flow rate due to gas expansion coupled with dry ice formation[18]). To ensure that we are not under-estimating likely leakage due to the extra difficulties in remediating blowouts on CO_2 wells, the values of mass lost during a blowout have been increased by 50% to account for the expected more rapid flow and longer remediation timescales. We acknowledge that this is an arbitrary increase but, given the lack of data comparing CO_2 to conventional blowouts, we believe this provides a conservative likely estimate. This increases our boundary between minor and major blowouts from 500 t CO_2 equivalent to 750 t CO_2 equivalent.

For sensitivity analysis, the data are described in terms of a lognormal distribution where the natural log of the values is taken twice (i.e. Ln(Ln(x)), where x represents the data in Supplementary Table 7 and from the HSE database[44] that release between 1 and 500 t CO_2 equivalent, multiplied by 1.5 to account for the 50% increase). For this lognormal distribution, the mean of the logs is 1.27 ± 1.21 (one standard deviation), which provides a representative fit for the original data, but over-estimates the logged data (Supplementary Figure 7). We use this mean (i.e. $e^{e^{1.27}}$) for the base case value.

Major blowout rates are treated separately for onshore and offshore cases due to the higher frequency of offshore blowouts. Representative ranges are taken as 1.63×10^{-6} to 1.33×10^{-4} blowouts per well per year for onshore and 4.74×10^{-5} to 2.48×10^{-4} blowouts per well per year for offshore environments (Supplementary Figure 5). These are based on the studies with the largest data sets; for production wells, studies involving fewer than 10,000 wells have been excluded. In order to model a realistic worst case scenario we then multiply the frequencies by two, to allow for the potential doubling of risk associated with injection rather than production[45]. For the sensitivity analysis, we assume a normal distribution where the maximum and minimum values represent three standard deviations from the mean. This results in well blowout frequencies of $1.35 \times 10^{-4} \pm 4.4 \times 10^{-5}$ blowouts per onshore well per year, and $1.48 \times 10^{-4} \pm 3.3 \times 10^{-5}$ blowouts per offshore well per year. We use these mean values as the base case values.

The blowout data in Supplementary Table 7 inform our estimate of the maximum and minimum amount of leakage during a major blowout. Documented blowout (or other significant leakage) durations range from 1 second to decades, with total fluid volume leaked ranging from 9.8×10^{-5} to 350 million m^3. Supplementary Figure 6 plots the magnitude of blowout releases against the blowout duration. Most blowout events last less than a year, many lasting less than day. This is corroborated by a study into Gulf of Mexico offshore blowouts between 1971-1978, which found 16 blowouts, unrelated to drilling, of durations between 1 hour and 55 days, with an average of 8 hours and a median of 2.5 days[46]. Three of the fluid release events compiled in Supplementary Table 7 lasted much longer than a year: the Bečej field CO_2 leak (39 years), the Kalle UGS facility (5 years), and the 22/4b North Sea blowout (21 years). In the case of the Bečej field, the blowout itself lasted 209 days, but leakage continued until the well was remediated 39 years later; as far as we can tell, no attempt was made to remediate the well before this time. The Kalle UGS facility in Germany was shown to have leaked for at least 5 years, but again, it seems that no attempt was made to remediate this. In these two cases, the lack of well remediation was an operational issue that would not occur in the case of a CO_2 storage site. The 22/4b North Sea blowout continued

for 21 years before successfully being remediated. This was a drilling-related gas blowout that was particularly difficult to plug because of the shallow depth of the reservoir. This scenario is therefore not analogous to a CO_2 storage scenario, where CO_2 storage and associated blowouts would occur from greater depths. We thus consider the yearly leak volumes shown in Supplementary Figure 6 to be representative and assume that blowouts taking place on injection wells will not continue for more than one year.

For a minimum / major blowout mass-loss, we adopt our minor / major blowout boundary of 500 t per event for the hydrocarbon industry data. The greatest volume of material released during a blowout of less than one-year duration that we have identified is an estimated 5.98 x 10^8 m^3 of oil and gas (~1.17 Mt CO_2 equivalent) released over 84 days during the Macondo / Deepwater Horizon blowout; we adopt this as a representative maximum mass-loss from documented blowouts. As discussed above, we then increase these values by 50% to account for greater losses from CO_2 wells, resulting in minimum and maximum values of 750 t and 1,749,687 t, respectively. We note that recent work[47] has concluded that CO_2 blowouts are likely to result in smaller gas losses than natural gas blowouts, due to the greater density of CO_2 in the reservoir and resulting lower discharge rate. However, we have chosen not to factor this possible reduction into our data compilation until this phenomenon has been independently verified and can be quantified for a wider range of conditions. For sensitivity analysis, the data are described in terms of a lognormal distribution where the natural log of the values is taken twice (i.e. Ln(Ln(x)), where x represents the data in Supplementary Table 7 and from the HSE database [44] that release between more than 500 t CO_2 equivalent, multiplied by 1.5 to account for the 50% increase). For this lognormal distribution, the mean of the logs is 2.57 ± 0.045 (one standard deviation), which provides a fit that envelopes, but over-estimates the original data (Supplementary Figure 8). To calculate the standard error, we take the number of data points (17) as n, giving a standard error of 0.011. We use this mean (i.e. $e^{e^{2.57}}$) as the base case value.

Supplementary Figure 7: Minor blowout losses

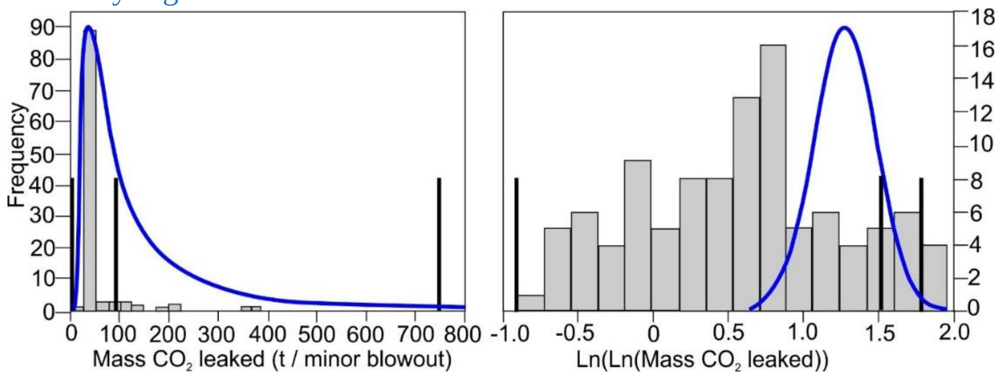

Active well minor blowout leakage. Histograms (grey boxes) and fitted lognormal distributions (blue lines) for the mass leaked during a minor blowout. Data from Supplementary Table 7. Black lines show the minimum, base case, and maximum values.

Supplementary Figure 8: Major blowout losses

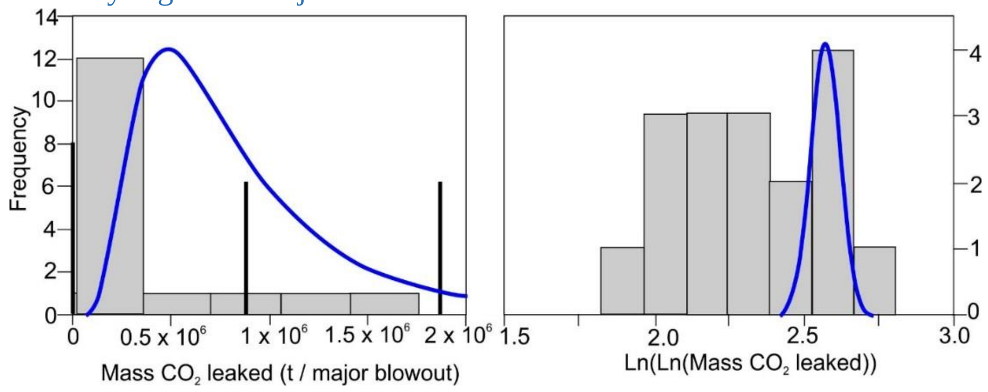

Active well major blowout leakage. Histograms (grey boxes) and fitted lognormal distributions (blue lines) for the mass leaked during a major blowout. Data from Supplementary Table 7. Black lines show the minimum, base case, and maximum values.

Supplementary Note 4: Estimating Leakage in Abandoned / Legacy Wells

4.1 Background and context

Abandoned wells pose different risks to those associated with active wells. While abandoned wells do not experience the operational stresses of active wells, it may not be possible to identify and monitor all abandoned wells within a storage site, and so leakage may go unnoticed, preventing its remediation. As frequency of pre-existing wells decreases with depth, the risk of leakage along an abandoned well can be reduced by selecting injection formations deeper than historically producing formations[48].

The identification and monitoring of all abandoned wells in a storage site is particularly relevant for regions with a long history of hydrocarbon exploitation. In such regions, many wells were drilled before comprehensive record keeping and regulation standards began, meaning that no records exist of the wells and their abandonment status (plugged / unplugged / plug integrity) is unknown. A recent survey of abandoned wells in Pennsylvania estimated that the majority of abandoned wells are not documented[49]. The two main field-techniques used for identifying undocumented abandoned wells are magnetic surveys and measurement of methane concentrations[50,51]. However, magnetic surveys will not identify wells where the steel casing has been removed or was never fitted. Methane will only leak from wells that penetrate formations containing buoyant fluids. Most wells are abandoned because they no longer produce or have never produced hydrocarbons and so methane surveys are unlikely to identify all abandoned wells. Furthermore, measurement of fluid flow up an abandoned well is the main way of testing abandoned well integrity, without requiring costly re-entry of the well. However, this method will only work if the well is penetrating pressurised reservoirs. If the reservoir is depleted, fluid flow up the well will be reduced[52], leakage potential will be under-estimated, and well integrity will be over-estimated. For many wells, it may be impossible to assess the integrity of well plugs and cement until the well bottom is pressurised by injection or migration of fluids into the reservoir(s) penetrated by the well. While this is unlikely to be an issue for regions with a highly regulated CCS industry, where we anticipate that regulators will require poorly documented wells to be re-entered, tested, and repaired, it may be an issue if CCS is developed in a region with a poorly enforced regulatory system.

4.2 Sources of abandoned well data

The amount of CO_2 that may leak from an abandoned well depends on a number of factors, including: the areal density and depth of pre-existing wells, proximity to the injection well, injection pressure, the CO_2 plume geometry, the permeability of the wells and the reservoir, the ability of the CO_2 to flow up the well (hydraulic head), and whether the well is open to overlying aquifers that may act as a sink for the leaking CO_2. Precise modelling of potential leakage from abandoned wells at a given storage site requires detailed constraints on all of these parameters, and also on the permeabilities of the geological formations, temperature and pressure (thus phase density and viscosity), injection volume and pressure, and appropriate model-grid spacing and equations of state[13,53,54]. For a more generalised model, we estimate the amount of CO_2 that may leak from abandoned wells via two sources: 1) Data of natural gas leakage or blowouts from abandoned well bores. 2) Mathematical models of CO_2 leakage along abandoned wells.

Gas leakage data from abandoned wells are available for some hydrocarbon fields, but these are unlikely to be representative of leakage from CO_2 storage reservoirs because wells tend to only be abandoned once they are no longer producing (i.e. reservoir pressure is low). As a result, such data would vastly under-estimate the amount of CO_2 likely to leak from an abandoned well. For example, a study on abandoned wells in Pennsylvania, USA, found that leakage rates were higher for abandoned gas wells than for abandoned oil wells[52], indicating that leakage strongly depends

on the presence of buoyant material in the reservoir. However, a more recent study on abandoned wells, also in Pennsylvania[49], found no correlation for high-emitters between abandoned well leak rate and proximity to underground gas storage, suggesting that the highest emitting wells may actually be representative of wells leaking from a gas-rich reservoir. For comparison, the highest emitter was an unplugged gas well from a non-coal area with a methane flow rate of 3.5×10^5 mg h^{-1} [49]; assuming standard temperature and pressure conditions, this is the equivalent of 8.45 t CO_2 $year^{-1}$. The average methane emissions from plugged gas wells in non-coal areas was 5.4×10^2 mg / h/ well[49], equivalent to 0.01 t CO_2 $well^{-1}$ $year^{-1}$. A mean leakage rate of 0.27 kg of methane $well^{-1}$ $year^{-1}$ (volumetric equivalent of 271 kg CO_2 $well^{-1}$ $year^{-1}$) was measured for abandoned wells during another study in Pennsylvania[55].

Data for blowouts occurring on abandoned wells in fields undergoing injection (CO_2, steam or water) may be more representative, but data are sparse. A study investigating the timing and mechanism of abandoned well leakage due to nearby steam injection in California found that most well failures leaked shortly after being abandoned and / or impacted by steam. This indicates that leakage is most commonly due to initial well defects[56]; while the timing of some blowouts indicates that they were caused by well degradation over time, these were a minority[56]. This study estimated that inactive wells contacted by steam floods would blowout at a rate of one per several thousand wells for initial defects during or shortly after injection[56]. For aging-related defects, the risk of blowing out over the longer term decreased over time, due to improvements in cementing practices, from 1/10,000 well years in the 1990s to 1/100,000 well years in the year 2000[56].

Various studies have modelled the amount of CO_2 expected to leak along abandoned wellbores and consider a range of storage and migration conditions. Many of these studies, which are summarised in Supplementary Table 7, model leakage of CO_2 out of the storage formation, and not necessarily leakage to the surface.

To assess how representative the models are of field conditions, we compare the model parameters to values experimentally derived for effective permeabilities for leaking wellbores (Supplementary Figure 9). One study used gas flow rates to determine effective well permeability for plugged (0.4 mD) and unplugged (17 mD) wells[52], but also noted significant difference in flow rate between gas and oil wells, and calculated higher effective permeabilities for gas wells than for oil wells. In their study, Kang et al noted that the effective permeability model assumes an infinite supply of gas and that, for wells with low gas contents the effective permeabilities will be an under-estimate[52]. For this reason, we take the upper ranges of their calculated effective permeabilities, rather than the quoted means, and assume that 10^0 and 10^2 mD are more appropriate values for plugged and unplugged wells, respectively.

Supplementary Figure 9: The relationship between permeability and leakage

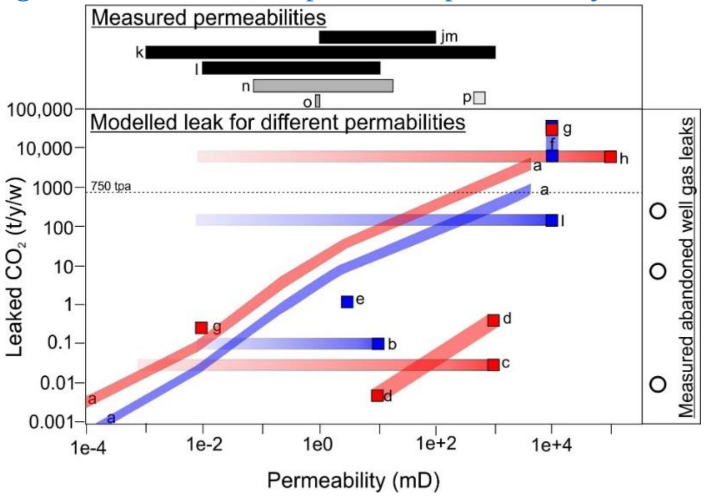

Modelled CO_2 leakage along abandoned wells, plotted against well effective permeability used in the models, compared to measured well permeabilities. Red symbols represent models of a pressurised reservoir. Blue symbols represent models where buoyancy is the sole driver of leakage (no overpressure). Black symbols are effective well permeabilities measured along entire wellbores. Grey symbols are local permeabilities measured in samples of well cement, either sampled from CO_2 wells or corroded in the laboratory. Letters refer to letters in Supplementary Tables 8 and 9. Measured leaks (circles) refer to the values described in the above text.

4.3. General abandoned well parameters - well density and condition

For simplicity, we make the unrealistic but conservative assumption that all abandoned wells penetrate the storage formation. The areal well density and proportion of degraded and leaking wells is likely to vary depending on past and contemporary drilling and abandonment history and regulations. Assuming that regions with an established hydrocarbon industry are targeted, we expect the areal density of abandoned wells to be different between offshore and onshore environments. The IPCC report on CCS (Figure 5.27)[57] estimated a hydrocarbon well density for the North Sea of up to 4,400 wells per 10,000 sq km (0.44 wells km^{-2}). This figure is assumed to be reliable as hydrocarbon exploration in the North Sea has been regulated (and thus documented) since the industry began in the 1960's. We take this value as an estimate of abandoned well density for offshore CO_2 storage regions. A 2009 study [58] estimated that Texas contains more than 125,000 wells over 50,000 km^2, equating to a well density of 2.5 wells / km^2. We use this value as an estimate of abandoned well density in onshore CO_2 storage regions.

We note that, in regions with an exceptionally long-lived hydrocarbon industry, there may be instances of wells not being recorded and being improperly abandoned. A study of Pennsylvanian hydrocarbon industry wells recently revised estimates of well numbers from 350,000 wells to up to 750,000 wells, based on studying historical records and historical aerial photographs[49]. In this case, an under-estimation factor of ~2.1 describes the difference between recorded and existing wells. We have built an under-estimation factor into our model, that allows us to quantify the impact of unidentified abandoned wells. In reality, the under-estimation factors will be lower than that found for Pennsylvania, because CO_2 storage is unlikely to be promoted in regions with such high uncertainty and potential risk of leakage. For our Offshore, and Onshore Well-Regulated scenarios, the under-estimation factor is 1. For our Onshore Poorly-Regulated scenario, we adopt a base case under-estimation factor of 1.55, with a minimum and maximum of 1.1 and 2.0, respectively, and assume a uniform distribution between these values.

To estimate well integrity, we consider the wells in terms of unplugged, degraded, and intact wells. We assume that all known wells are plugged (or at least are remediated with high quality plugging procedures), and so unplugged wells are only an issue where the well under-estimation factor is greater than 1. For our Onshore Poorly-Regulated scenario, we estimate the proportion of unknown abandoned wells that are unplugged to be 30%, based on estimates of abandoned well status in Pennsylvania[52]. We use the frequency of continuously-leaking active wells as a proxy for degraded wells, giving base case scenarios of 14.5% and 7.5% for offshore and onshore wells, respectively. The remaining wells are assumed to be intact. For sensitivity analysis, we assume the same lognormal distribution as applied to continuously leaking active wells, discussed in Section 2.2.2.

Because leakage from abandoned wells depends on the number of abandoned wells and well integrity issues, both of which vary over time, we have calculated two different abandoned well leakage rates to be applied to the injection (AB1) and post-injection (AB2) phases of the program. We assume that: (1) the storage site is monitored during the injection period; (2) any blowouts are remediated with high quality plugging; and (3) all known abandoned wells are monitored, allowing rapid identification and remediation of degraded wells. Once injection ceases, the injection wells are converted to abandoned wells. During AB2, all known wells are assumed to be plugged and intact, with any degraded wells having been repaired during AB1.

4.4 Abandoned well continuous leakage parameters

We expect smaller leaks to occur, similar to continuous leakage in active wells, with the amount of CO_2 leaked dependant on well integrity. Estimating the amount of CO_2 that may be leaked from wells with different levels of integrity is difficult as this also depends on local reservoir conditions. Maximum modelled leak rates that would not be classed as blowouts (as defined above) are ~300 t CO_2 year^{-1}. This is comparable to the higher documented gas flow rates from abandoned wells[52] and from active wells (Supplementary Table 5). We use this value (300 t CO_2 year^{-1}) as a proxy for leakage from degraded wells and assume that this leak rate is low enough that it might not be detected without frequent monitoring and is thus constant during AB1. To estimate leakage from relatively intact abandoned wells, we refer back to the continuous leakage rates for active wells, presented by Marlow[35], who found that up to 5.4% of wells leaked up to 230 t CO_2 equivalent per year (Supplementary Table 5). To avoid under-estimating leakage from abandoned wells, we thus assume that 5.4% of intact wells will leak 230 t CO_2 year^{-1} and the remainder will leak 0.004 t CO_2 well^{-1} year^{-1} (the minimum modelled leak rate for wells experiencing overpressure in Supplementary Figure 9 and Supplementary Table 8).

4.5 Abandoned well discrete event parameters

For short term blowouts (one per several thousand wells[56] over the 30 year injection period), we consider minimum, maximum, and mid-point probabilities of one per 9,000 wells (1/9000/30 = 3.7×10^{-6}), one per 2,000 wells (1/2000/30 = 1.7×10^{-5}), and one per 5,500 wells (1/5500/30 = 6.1×10^{-6}), respectively. For the sensitivity analysis, we assume a lognormal distribution based on these minimum, maximum, and base case values that is described by the mean of the natural logs = -8.6125 ± 0.23 (one standard deviation). We use this mean (i.e. $e^{-8.6125}$) as the base case. The model has been constructed to include the possibility of unplugged wells being present (for scenarios with unidentified wells – i.e. well under-estimation factor >1), and we assume that any unplugged wells will also blowout during the injection period.

For the long term blowout rate (AB2), we consider minimum, maximum, and mid-point probabilities of one per 100,000[56] well years (1/100,000 = 1×10^{-5}), one per 10,000 well years[56] (1/10,000 = 1×10^{-4}), and one per 50,000 well years (1/50,000 = 2×10^{-5}), respectively. For the

sensitivity analysis, we assume a uniform distribution between these minimum and maximum values.

We also assume that abandoned well blowout events are similar to those of active wells, but that there may be delays in identifying the location and thus the remediation of smaller blowouts (c.f. up to 750 t vented in total for active wells). Discounting the three longest lasting blowouts described in Supplementary Table 7, the median blowout duration is 22 days and we use this to calculate a likely CO_2 venting rate of 34 t CO_2 day^{-1} during a small blowout, and scale this to a scenario where identifying and remediating the blowout takes 1.5 years. This gives a conservative minimum CO_2 loss of 18,615 t CO_2 per abandoned well blowout. For a mid-point, we use the venting rate of the Bečej Field CO_2 leak (678,500 t CO_2 year^{-1}) and assume that such a large blowout would be identified and remediated within a year. For a maximum blowout loss, we adopt the maximum value for active well blowouts (1,749,687 t CO_2 in the Macondo / Deepwater Horizon event).

For sensitivity analysis assessing the impact of the mass of CO_2 lost during an abandoned well blowout, we describe the minimum, maximum, and mid-point values in terms of a lognormal distribution where the mean of the logged values is 13.4 ± 0.35 (one standard deviation). In this case, we work with the standard deviation, rather than the standard error, because the number of blowouts per year is expected to be so small that the standard deviation and standard error will be comparable.

Supplementary Table 2: General abandoned well input parameters

**General Parameters**	**Value**
Injection phase blowout rate: (plugged wells)	1 / 5,500 wells over the 30 year injection period (Max: 1 / 2,000 wells; Min: 1 / 9,000 wells) = 6.06×10^{-6} blowouts per well per year (Max: 1.67×10^{-5} b well^{-1} year^{-1}; Min: 3.70×10^{-6} b well^{-1} year^{-1})
Post-injection blowout rate:	1/10,000 well^{-1} year^{-1} = 1.0×10^{-4} blowouts well^{-1} year^{-1}
Mass of CO_2 lost per blowout:	
Minimum	18,615 t CO_2
Base Case	678,500 t CO_2
Maximum	1,749,687 t CO_2
Well leak rates:	
Degraded wells	300 t CO_2 year^{-1}
Intact wells with high leak rate	230 t CO_2 year^{-1}
Intact wells with low leak rate	0.004 t CO_2 year^{-1}

General parameters used to estimate abandoned well leakage in all scenarios.

Supplementary Note 5: Natural Leakage

CO_2 may potentially leak out of a geological storage site via natural pathways (i.e. diffusion through the cap rock, or advection along faults and fractures). However, in a review of global natural and man-made CO_2 geological subsurface accumulations, Miocic et al[59] noted that only 10 out of 76 known CO_2 fields show conclusive or inconclusive evidence of leakage.

Work by numerous authors[60–63] has concluded that diffusive losses through the caprock matrix will be negligible under CO_2 storage timescales. Busch et al[61] and Deming[64] have shown that capillary pressure CO_2 breakthrough of the caprock occurs after hundreds to thousands of years for medium to low permeability caprocks with a realistic thickness of 100m. Hence, diffusion of CO_2 through the cap rock is not expected to result in significant loss from the storage reservoir.

Fractures are the most likely route for CO_2 leakage from the reservoir through the caprock. Pre-existing fractures may be present, or overpressure as a result of CO_2 injection may cause hydraulic fracturing or re-activation of fractures within the seal rock[65,66]. During monitoring of the world's first commercial scale on-shore CO_2 storage project, In Salah, evidence for CO_2 migration into the lower, shaly seal rock layer was observed. This was interpreted to have occurred as a result of tensile opening of a fracture zone in response to pressurisation during CO_2 injection[67]. As with leakage along abandoned wells, at the individual site-scale, leakage through fractures will be influenced by a host of parameters, including the permeabilities of the conduits, the reservoir rocks, and the pressure in the reservoir and in all of the formations contacted by the conduit; flow through fractures is influenced by thermo-hydraulic-mechanical and chemical (THMC) processes. These processes are intrinsically linked such that one process affects the initiation and progress of others[68].

Predicting leakage along natural flow pathways is difficult due to uncertainties in whether a fault or fracture will act as a migration conduit or as a seal, and this may also vary over time[69–71]. Using this information to quantify a natural leakage parameter is challenging even when CO_2 fluxes from point sources are quantified - e.g. gas seeps, along fractures, of methane $\pm$ CO_2 $\pm$ heavier hydrocarbon gases from hydrocarbon fields, volcanoes, mud volcanoes, and along faults[72–81]. This is due to difficulties in extrapolating from point source data to predict areal fluxes, due to the uncertainties in fracture density, and fracture permeability. Even in a highly faulted area, only a very small proportion of the surface will be acting as a gas flow conduit at the square-kilometer scale[74,82]. Estimates of gas fluxes from entire fields or regions are thus more appropriate to inform our natural leakage parameter, but are less common. Compiled data on gas flux are shown Supplementary Figure 10 and their applicability to a CO_2 storage model is discussed below.

Italy is a tectonically active country noted for a high rate of geological CO_2 emissions. It is estimated to have a flux rate of geological CO_2 of 20-60 Mt CO_2 $year^{-1}$ [83]. Assuming an area of 294,140 km^2 [84], this gives geological CO_2 fluxes of between 68 and 204 t km^{-2} $year^{-1}$. The tectonically active Iceland also has a high degassing rate, and is estimated to emit 0.16 to 2 Mt CO_2 per year [85]. Assuming an area of 100,250 km^2 [84], an average geological CO_2 flux of between 1.6 and 20 t km^{-2} $year^{-1}$ is estimated for Iceland. Both Iceland and Italy's high degassing rates are a symptom of tectonics creating flow pathways (fractures and faults) in the crust. Such regions are useful for determination of possible CO_2 fluxes in tectonically active areas, but as these will likely not be the target of CO_2 storage sites due to the prevalence of active faults and fractures, their degassing rates are not relevant here.

At a global scale, degassing of CO_2 from the deep Earth via volcanism is a large-scale proxy of degassing via faults in a sedimentary basin. Estimates of global degassing of CO_2 via volcanoes range from 65 Mt per year, based on SO_2 fluxes[86], to 6×10^{12} moles of C (264 Mt CO_2), based on CO_2 and He degassing from ridges, island arcs and plumes. Assuming a global area of 148.3 million km^2 [87], this gives an average global CO_2 flux of between 0.4 and 1.8 t CO_2 km^{-2} $year^{-1}$.

Similarly, total global seepage of methane from petroleum systems has been estimated at 14-28 Mt methane per year emitting from an area of 8×10^6 km^2 [88]. Converting this mass of methane to a volume at standard temperature and pressure and calculating the equivalent mass of CO_2, gives a total of 38.5 to 77 Mt, equivalent to between 5 and 10 t CO_2 km^{-2} $year^{-1}$. As most petroleum systems are associated with sedimentary basins, we consider this generalised gas flux to represent the high end of the likely range of CO_2 leakage rates along natural pathways.

As noted by Miocic et al[59], the majority of naturally occurring CO_2 accumulations do not show evidence of CO_2 leakage to the surface. For example, at the Farnham CO_2 field, measured CO_2 fluxes were 0.5 – 3.7 g m^{-2} d^{-1} [89], equivalent to 183-1,351 t CO_2 km^{-2} $year^{-1}$; these fluxes were concluded to be shallow and biogenic in origin, based on their similarity in flux rate to biogenic sources in arid regions [89]. Background fluxes of surface CO_2, due to biological action and respiration, are on the order of 60-1260 g of carbon per m^2 per year (equivalent to 220 to 277,200 t CO_2 km^{-2} $year^{-1}$) [90]. Measurements of CO_2 fluxes must, therefore, rule out and / or correct for biological CO_2 when quoting flux rates for deep-sourced gases. If this is undertaken, the resulting flux rates are often very small or imperceptible. Thus, we do not consider the CO_2 fluxes measured at Farnham to represent natural leakage of deep CO_2.

Natural gas seeps in the Upper Ojai Valley in Southern California leak an estimated 55 m^3 of gas per day [75]. The gas is a mixture of CO_2, methane, and heavier hydrocarbons and emanates from seeps and vents within the valley associated with numerous faults. We estimate an area of 30 km^2 for the Upper Ojai Valley, measured via Google Earth, and using the topographic trace of the San Cayetano Fault as the northern boundary, and the crest of the Sulphur Mountain ridge as the southern boundary. This gives an emission rate of 2 m^3 of gas per km^2 per day, equivalent to 1.31 t CO_2 per km^2 year, assuming standard temperature and pressure.

Twenty three Mt CO_2 have been injected for enhanced oil recovery at the Rangely Field, Colorado, and surface CO_2 fluxes were measured to quantify micro-seepage of the injected CO_2[91]. Fluxes of 8,600 t CO_2 per year were measured over the 73 km^2 of the field, but carbon isotope data revealed that the vast majority of this gas was shallow and biogenic in origin, with geological seepage contributing an estimated 170 t CO_2 $year^{-1}$ for the site [91]. This equates to 2.2 t CO_2 km^{-2} $year^{-1}$.

Our approach to incorporate leakage rates into the SSC draws on gas flux data from multiple scales - from the regional (Rangely Field and Ojai Valley) and global (methane seepage from total petroleum systems, and global emissions of deep-sourced CO_2) – and adapts this for a CO_2 storage setting. We adopt a most likely natural leakage rate of 2 t km^{-2} $year^{-1}$, based on the areal fluxes from the Ojai Valley natural seeps and the Rangely EOR field. As a lower limit, we take the lowest estimate of global fluxes of geological CO_2 (0.44 t km^{-2} $year^{-1}$), and as an upper limit, we take the highest estimate of average areal flux from total petroleum systems (10 t km^{-2} $year^{-1}$).

For the sensitivity analysis, we describe the minimum, maximum, and most likely values in terms of a lognormal distribution where the mean of the logs = 0.693 ± 0.37 (one standard deviation), and take this mean (i.e. $e^{0.693}$) as the base vase value.

Supplementary Figure 10: Natural leakage fluxes

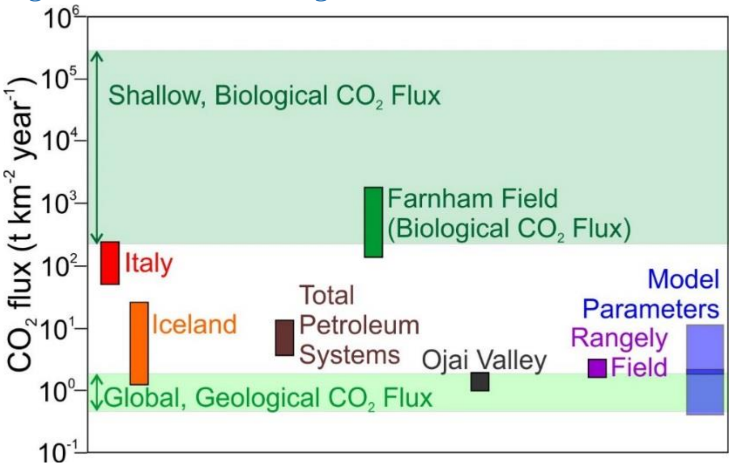

Ranges of documented natural leak rates for CO_2, or for hydrocarbon gasses converted to equivalent mass of CO_2.

Supplementary Note 6: Reduction of leakage rate with time

The leakage model is based on measured and modelled surface fluxes and is not directly linked to subsurface conditions. However, leakage is not expected to be constant over time but expected to decrease as the buoyancy of the CO_2 decreases by reduction of the mobile CO_2 remaining in the plume (through leakage, immobilisation and pressure dissipation). To assess the rate of leakage decay, we compared gas flux rates over time from CO_2 leakage models, natural gas production, and from a large natural gas blowout.

Zahasky and Benson[92] carried out TOUGH2 simulations to model the evolution of CO_2 leakage out of a storage reservoir with various different mitigation techniques. Their model outputs (Figures 11 and 12 of their paper) plot the leakage rate in Kg s^{-1} over 500 years. The "passive mitigation" result (i.e. injection stops once leakage is identified, but no other mitigation measures are carried out) shows a roughly exponential decrease in leakage rate over time. Results are available for two scenarios: (1) where a leak is discovered and the injection stopped after 5 years of injection; and (2) after 10 years of injection. This decrease in modelled leak rate is plotted on Supplementary Figure 11 as a proportion of maximum leakage rate.

Jordan et al[93] used a Monte-Carlo approach with a multiphase reservoir simulator to investigate CO_2 flux to the surface along leaking wellbores over 200 years. Their Figure 16a plots the leakage rate in tonnes per year over time and all simulations show a decrease in leakage rate. Almost half of the simulations approximate an exponential decrease in leakage rate, while others show plateaus and an overall step wide decrease in leakage rate over time. The decrease in leakage rate for Jordan et al's 50% of realisations is plotted as a proportion of maximum leakage over time on Supplementary Figure 11.

Réveillère et al[94] investigated using pressure control to mitigate CO_2 leakage into an overlying aquifer, using TOUGH2/ECO2N multiphase flow transport simulations. While this simulation considers leakage to another aquifer, rather than to the surface, we consider the passive leakage decay rate to be a valid approximation for leakage to the surface. We have plotted the decrease in leakage rate over time, based on Réveillère's Figure 11, in Supplementary Figure 11.

The Aliso Canyon natural gas blowout, California, began in October 2015 and continued for over three months. Methane emissions from the blowout were measured along plume transects by research aircraft on thirteen flights over the course of the blowout[95]. These emissions data suggest an initial period of maximum leakage rate, followed by an exponential decrease in leakage rate, beginning after almost 1.5 months. The leakage rate appeared to plateau at less than 50% of the initial leakage rate, ~1 month before the well was finally brought under control and the blowout stopped[95].

Eight years' worth of gas production data from Alberta, Canada were compiled by Samson[96], to investigate whether production trends were sufficient to meet future demand for natural gas. These data are from multiple gas fields and multiple wells, and incorporate commissioning and closure of wells each year. It is possible that the decline in gas production reflects outside market forces, such as the price of gas, or regulation issues that decrease the number of active wells, but given the context of an increasing demand for natural gas, this seems unlikely and we assume that the decrease in productivity is mostly controlled by depletion of the gas reservoirs. The rate of decrease in production (based on Samson's Figure 12 – calculated daily production rate) is plotted on Supplementary Figure 11. The decline in production rate is similar to the decline in leakage rate modelled by Réveillère et al, and by the decline in leakage rate during the Aliso Canyon blowout.

Plotting these leakage decay rates against time (Supplementary Figure 11) shows that all modelled and measured leakage rates show an approximately exponential decrease over time, albeit with different exponential rates.

To incorporate leakage decay into our model, we created two exponential decay curves that envelope the data. The longest data set is the Zahasky and Benson[92] model that runs for 500 years, while other models and measurements are for much shorter timescales. To avoid inaccuracies in extrapolating the leakage reduction curves forward in time beyond the range of data, our curves assume that leakage decreases to a point, and then remains constant over time. These curves are of the form:

$$\%\ maximum\ leak\ rate\ at\ time\ "t" = A + (100 - A) * e^{-Bt} \qquad \text{Eq. [1]}$$

Parameters A and B were iteratively determined to produce curves that envelope the data and parameter A represents the minimum long-term leakage rate as a percentage of the maximum. For the high leakage reduction rate envelope (i.e. leakage reduces quickly), A= 3 and B = 0.5. For the low reduction rate envelope (i.e. leakage reduces more slowly and plateaus at a higher level), the curve was fitted to the Jordan et al data, and resulted in A = 53 and B = 0.03. We also fitted a curve to the Zahasky and Benson[92] data, which gave A = 3 and B = 0.0143.

For sensitivity testing of these leakage reduction curves in our model, we take maximum and minimum values of A and B based on the three curves (A = 3–53; B = 0.0143–0.5). For parameter A, we notice that the majority of the data suggest that leakage will plateau at less than 20% of the original leak rate, and we consider that a skewed distribution is the most appropriate to apply to this data range. We thus apply a triangular distribution to Parameter A with minimum, maximum, and most likely values of 3, 53, and 12, respectively. The most likely value (12) is taken as the base case value. For Parameter B we apply a uniform distribution from 0.0143 to 0.5, and take the midpoint (0.257) as the base case.

When carrying out a Monte Carlo analysis, we use 10,000 realisations. To show the range of leakage reduction curves that will be produced during the Monte Carlo analysis, a sample of 10,000 curves produced by selecting random numbers (within the defined ranges) for Parameters A and B are shown in Supplementary Figure 11 as red lines.

Supplementary Figure 11: Leakage reduction

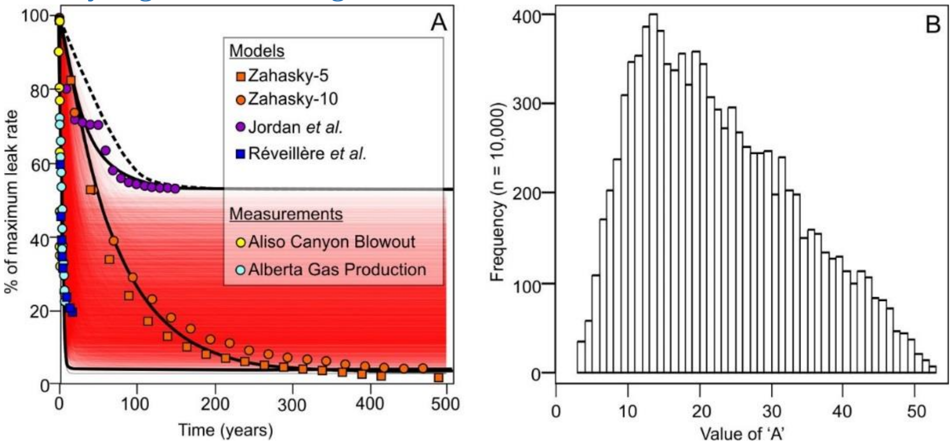

A) Modelled and measured leakage reduction rates (symbols), enveloping and base case scenario curves (black lines) and 10,000 realisations of leakage reduction curves based on a triangle distribution for Parameter A and a continuous distribution for Parameter B (red lines). Black dotted line shows the minimum reduction curve based on the Maximum Parameter A (envelope) and minimum Parameter B (base case). B) Histogram of 10,000 randomly selected values of Parameter A assuming a triangular distribution with minimum and maximum values of 3 and 53, respectively, and a most-likely value of 12.

Supplementary Note 7: Residual Saturation Trapping

To model residual trapping we use the data compiled by Burnside and Naylor [97] for published residual saturation values (Supplementary Table 10). These data form a normal distribution (Supplementary Figure 12), described by a mean of 0.5800 (58.00%), which we take as the base case value, and a standard deviation of 0.1897 (18.97%). The data compilation comprises 44 data points, giving a standard error of 0.0286. Please see the main text for a discussion on the relevance of laboratory and simulated residual trapping values.

Supplementary Figure 12: Residual Trapping

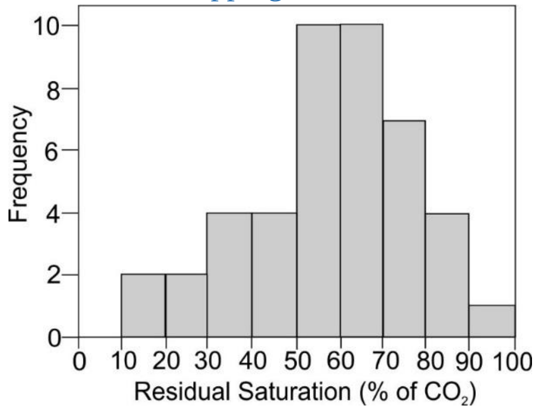

Histogram of the residual trapping data, approximating a normal distribution.

Supplementary Note 8: Chemical Trapping

Chemical trapping refers to both solubility and mineral trapping, where free-phase (gaseous or supercritical) CO_2 dissolves into the groundwater and / or precipitates as carbonate minerals.

Estimates on the proportion of gaseous CO_2 that can dissolve into reservoir brines vary from 6.5% [98] to 90%[99] on geological timescales. Estimates for mineral trapping range from 2%[100] to 43%[98]. These two processes are likely to interact and occur in equilibrium with each other. While various studies have investigated rates of solubility or mineral trapping individually, studies investigating the rates of both processes occurring together, and over geological timescales, are rare. The most comprehensive model we have found is that from Xu et al [101], which models the variation in proportions of gaseous, dissolved, and mineralised CO_2 over 10,000 years. This model uses the mean rock composition of the Frio formation (Gulf Coast, USA) as target unit. The hydrogeological parameters (e.g., pressure, temperature, brine composition, porosity, permeability, etc.) were selected to be representative of the storage conditions at 1km depth.

The evolution of the total CO_2 injected into the reservoir, and how the CO_2 is sequestered into different phases, according to the Xu et al model (Figure 5 their paper[101]) shows the evolution of the total CO_2 injected into the reservoir and how the CO_2 is sequestered into different phases (gaseous, dissolved, mineralized). Mineral trapping begins at a significant rate at 500 years and increases with time. After 10,000 years, the proportion of CO_2 sequestered by mineral trapping is comparable to CO_2 dissolution in pore waters. The Xu et al[101] model results are expressed in absolute CO_2 values. We converted these into relative values by dividing them by the total injected CO_2, and determined equations that describe the CO_2 partitioning as a function of time.

Xu's modelling results predict that dissolution of the CO_2 will increase during the first decades of the model and stabilise its effect at ~100 years, expressed with the following approximation:

$$\%\,Solubility\,Trapping\,(t) = 0.204 \cdot t^{0.0342} \quad \text{Eq. [2]}$$

where t is the time in years. The mineral trapping increases steadily with time until reaching a trapping level similar to the solubility trapping (2-5 kg of CO_2 per m^3 of reservoir), following the expression:

$$\%\,Mineral\,Trapping\,(t) = (1.67 \cdot 10^{-13} \cdot t^3) + (2.90 * 10^{-9} \cdot t^2) + (1.40 \cdot 10^{-5} \cdot t) \quad \text{Eq. [3]}$$

As the Xu et al[101] simulation is the most comprehensive chemical model available, we use it to simulate chemical trapping in the SSC. Details on how these rates are built into our chemical trapping model are provided in Section 4.

Supplementary Note 9. The Leakage Model (2)

The leakage model - or Leakage model (2) - calculates the amount of CO_2 expected to leak from storage, based on measured and expected surface fluxes. It combines leakage calculated for active wells, abandoned wells, and natural pathways, and reduces over time once injection ceases (Section 2.5). This section describes how the active well, abandoned well, and natural pathway leakage rates are calculated.

9.1. Active Well Leakage (2a)

Below is a step-by-step description of and list of the parameters used to calculate active well leakage. Labels in [*square brackets and italics*] refer to the label used in the R code, which are also listed in the methods section of the main text.

Scenario specific inputs:

- Slow leak frequency (% wells leaking) [*ActiveWellFreq*]
- Major blowout frequency (events well^{-1} year^{-}) [*MajorBlowFreq*]

General inputs:

- Injection target
- Injection rate per well
- Injection period Slow leak rate (t CO_2 well^{-1} year^{-1}) [*SlowLeakInjector*]
- Minor blowout frequency (events well^{-1} year^{-1}) [*MinorBlowFreq*]
- Minor blowout mass (t CO_2 event^{-1}) [*MinorBlowout*]
- Major blowout mass (t CO_2 event^{-1}) [*MajorBlowMass*]

Calculate the number of injection wells:

- Injection target / injection period / injection rate = Number of injection wells

Calculate loss via slow leakage:

- Slow leak frequency * slow leak rate = average t CO_2 well^{-1} year^{-1}

Calculate loss via blowouts:

- Minor blowout frequency * minor blowout mass = average t CO_2 well^{-1} year^{-1}
- Major blowout frequency * major blowout mass = average t CO_2 well^{-1} year^{-1}

Calculate Active Well Leakage

- Slow leak + minor blowout + major blowout

9.2 Abandoned Well Leakage (2b)

Below is a step-by-step description of and list of the parameters used to calculate abandoned well leakage. Labels in [*square brackets and italics*] refer to the label used in the R code, which are also listed in the methods section of the main text.

Scenario specific inputs for initial model set-up:

- Number of injection wells (see 3.1)
- The areal extent of the CO_2 plume [*MeanPlumeArea*]
- Measured abandoned well density (wells km^{-2}) [*KnownWellDensity*]
- Well under-estimation factor [*wellUnderEst*]
- Proportion of wells that are plugged / unplugged [*UnPlugWells%*]

- Proportion of plugged wells that are intact / degraded [*DegradWells%*]
- Proportion of intact wells with a high continuous leak rate

Calculations for initial model set-up:

- Calculate the density of known and unknown plugged and unplugged wells.
- Calculate the density of known and unknown degraded and intact wells.
- Convert known plugged wells to known intact wells.

At end of initial model set up we should have:

- Unknown, unplugged well density
- Unknown, degraded well density
- Unknown, intact well density
- Total unknown well density
- Known intact well density
- Total known well density

AB1 &AB2 General Inputs:

- Short term blowout rate for plugged wells (events well^{-1} year^{-1}) [*PlugBlowoutYear*]
- Long term blowout rate for plugged wells (events well^{-1} year^{-1}) [*BlowoutWellYear*]
- CO2 loss per blowout (t CO_2 event^{-1}) [*CO2largeBlowout*]
- Proportion of intact wells with a high leak rate [*IntactHighRate%*]

AB1 &AB2 Scenario Specific Inputs:

- Injection period (years)
- Degraded well leak rate (t CO_2 well^{-1} year^{-1}) [*CO2degraded*]
- Intact well, high leak rate [*CO2intactHigh*]
- Intact well, low leak rate [*CO2intactLow*]

AB1 blowouts:

- Calculate number of blowouts
  - 100% unplugged wells blow out during injection period
    Unplugged wells / Injection years = unplugged blowouts (events km^{-2} year^{-1})
  - Blowout rate * unknown plugged wells = blowouts from unknown plugged wells (events km^{-2} year^{-1})
  - Blowout rate * known wells = blowouts from known wells (events km^{-2} year^{-1})
  - Sum the above
- Calculate the mass of CO_2 lost during blowouts: (Loss event^{-1}) * (events km^{-2} year^{-1})

AB1 continuous leakage:

- From degraded wells:
  - Calculate the total degraded well density (known + unknown)
  - Multiply by degraded well leak rate = loss from degraded wells (t CO_2 km^{-2} year^{-1})
- From intact wells:
  - Calculate the total intact well density (known + unknown)
  - Calculate the proportion of high and low leak wells (multiply by proportion of high leak rate intact wells) = high and low leak rate intact wells km^{-2}
  - Calculate the loss from high leak rate intact wells (*intact well high leak rate)

    - Calculate the loss from low leak rate intact wells (*intact well high leak rate)
- Sum the continuous leak rates

AB1 Leakage:

- Sum the continuous and blowout leakage to calculate the total leakage.
- This represents the maximum leakage rate, which assumes that the injected CO_2 plume has reached its maximum extent and highest leakage risk. This condition is not appropriate for the early stages of injection; therefore, we calculate AB1 leakage to increase linearly from 0 to the maximum over the injection period.

AB1-AB2 transition – well conversions

- All active injection wells are converted to known, intact, abandoned wells.
- All unplugged wells convert to known, intact wells.
- Unknown plugged wells that blew out convert to known intact wells. These are assumed to be from unknown degraded wells, the density of which is reduced accordingly.
- All known wells undergo monitoring and remediation = intact wells.
- Unknown intact wells remain constant.

AB2 Blowouts

- All wells are now plugged, so blowouts / year = total well density * long term blowout rate.
- Multiply by the loss per blowout = t CO_2 km^{-2} $year^{-1}$.

AB2 Continuous leakage

- From degraded wells:
    - Multiply degraded well density by yearly loss.
- From intact wells:
    - All known wells are monitored (and remediated if necessary) and have the low leak rate.
    - Calculate the density of unknown intact wells with the low leak rate.
    - Sum the above for the total well density for intact, low leak rate wells.
    - Multiply by the low leak rate for loss in t CO_2 km^{-2} $year^{-1}$.
    - Calculate the density of unknown intact wells with the high leak rate and multiply this by the high leak rate, for loss in t CO_2 km^{-2} $year^{-1}$.
- Sum the above for total continuous loss.

9.3 Natural Leakage (2c)

Natural leakage for each scenario is as determined in Section 2.4.

9.4 Combined Leakage Model (2)

The combined leakage model is split into injection and post-injection phases.

Injection Phase:

- The number of injection wells are calculated by dividing the injection target by the injectivity per well.
- The total yearly leakage from active wells is calculated by multiplying the active well leak rate by the number of injection wells.

- The area impacted by the CO_2 plume is calculated by multiplying the injection target by the area:mass ratio of the plume.
- Total leakage from abandoned wells and natural pathways are calculated by multiplying the leak rates (in t km^{-2} $year^{-1}$) by the area impacted by the plume.
- Total yearly leakage for the injection period is calculated by:
    - Total active well leakage + Total abandoned well leakage [AB1] + Total natural pathways leakage.
- To account for lower leakage levels while the reservoirs are being filled, the leakage rate increases from zero to maximum levels over the 30-year injection period, via a linear progression.

Post injection phase:

- Total yearly leakage for the post-injection period is calculated by:
    - Total abandoned well leakage [AB2] + Total natural pathways leakage.
- This yearly leakage rate constitutes the maximum leak rate.
- The true leak rate for a given year is calculated by multiplying the maximum leak rate by the proportional leak, as defined by Equation 1 in Section 2.5.
- The leakage amount of CO_2 leaked per year is calculate for years 1 to 10,000, along with the cumulative leakage for each year.

Supplementary Note 10: The immobilisation models (1)

10.1 Residual Trapping (1a)

We assume that residual trapping takes place as the plume of injected CO_2 migrates through the reservoir, and it reaches its maximum at the end of injection period (i.e., when the CO_2 plume has likely reached its maximum extent):

$$Residual\ Trapping\ (t) = \begin{cases} (I * RES\%) \cdot \left(\frac{t}{30}\right) & if\ \ t < 30 \\ I \cdot RES\% & if\ \ t \geq 30 \end{cases} \qquad \text{Eq. [5]}$$

where *t* is the time in years, *I* is the CO_2 injection targets (1.2 x 10^{10} t CO_2) and *RES%* is the percentage of residual trapping calculated from the data in Supplementary Table 10.

During the post injection stage, the proportion of CO_2 that is residually trapped will decrease over time, as chemical trapping consumes both mobile and residually trapped free-phase CO_2. To simulate this, residual trapping is applied to the injection target minus the calculated chemically trapped CO_2.

10.2 Chemical Trapping (1b)

The equations used to calculate the chemically trapped CO_2 are described in Section 2.7 and are applied to the amount of CO_2 remaining in the reservoir after leakage has been subtracted. The chemically trapped CO_2 consumes both residually trapped and mobile free-phase CO_2 (see Section 4.1).

Supplementary Note 11: The integrated model (1)

The Storage Security Calculator combines the Leakage and Immobilisation models by calculating the amount of CO_2 leaked and immobilised for each year and summing these values. This value is subtracted from the total amount injected (i.e. the injection target, once injection has ceased) to give the amount of mobile (i.e. leakable) CO_2 remaining in the reservoir. The model is projected forwards until 10,000 years, or until no mobile CO_2 remains, whichever occurs earlier.

Supplementary Note 12: The R-code for the Storage Security Calculator

Example code for the SSC is provided for the Offshore Scenario, and included in the Supplementary Information as a separate file (Offshore-SSC.R).

Supplementary Table 3

Area: Mass ratios (CO_2 equivalent) of gas fields in the UK North Sea. Data from Gluyas and Hitchens[3].

**Field**	**Area (km^2)**	**Gas vol. in reservoir (M^3)**	**CO_2 equiv. (Mt)**	**Area (km^2): Mass (Mt)**
Albury	1.2	1,103,252	0.77	1.57
Brown	1.5	3,174,529	2.22	0.67
Beaufort	1.7	3,974,288	2.78	0.61
Windemere	8.0	9,030,161	6.32	1.27
Malory	0.4	9,075,897	6.35	0.06
Mercury	10.4	10,202,011	7.14	1.46
Saltfleetby	11.6	10,319,900	7.22	1.60
Waveny	7.7	10,478,463	7.33	1.04
Bessemer	4.2	12,419,649	8.69	0.49
Boulton B	15.0	13,630,460	9.54	1.57
Sean E	4.1	16,346,516	11.44	0.36
Davy	6.0	24,055,534	16.84	0.36
Gawain	11.1	24,449,748	17.11	0.65
Corvette	3.2	25,753,641	18.03	0.18
Sean N	5.0	30,395,097	21.28	0.23
Neptune	6.5	32,010,296	22.41	0.29
Camelot	8.9	37,018,317	25.91	0.34
Schooner	55.0	60,382,863	42.27	1.30
Sean S	9.8	61,415,993	42.99	0.23
Brae N	19.0	81,405,203	56.98	0.33
Barque	36.4	169,652,407	118.76	0.31
Morecambe North	24.0	207,920,559	145.54	0.16
Leman	253.0	483,359,317	338.35	0.75
Indefatigable	155.4	583,723,509	408.61	0.38
Morecamb S	83.8	989,148,493	692.40	0.12

Supplementary Table 4

Frequency of continuously leaking hydrocarbon wells in the literature (Table extends over 2 pages).

Location	% wells leaking	Notes & References
Onshore:		
Bahrain	13.1	[102] in [38]
Canada	22	[36] in [38]
Canada: Alberta	4.6	[29]
Canada: Pembina Field	1.3	[103]
Canada: Zama field	6	[103]
China Daqing	16.3	[104] (cited as “Zhongxiao” in [38])
USA Marcellus Shale	6.26	[38]
USA Marcellus Shale	4.8	[105] in [38]
USA UGS	1.9	[57] (as “IPCC” in [38])
USA: Pennsylvania	3.4	[106] in [38]
USA: Marcellus Shale	4.49	[107](Table 4 in reference)
USA UGS	6.1	[35]
Onshore summary:	***Min: 1.3%; Max: 22%; Average: 7.5 ± 6.3% (1 s.d.)***	

Offshore:		
GoM: Brazos	14	[19] (Fig 2 in reference)
GoM: Cameron	8	[19] (Fig 2 in reference)
GoM: East	7	[19] (Fig 2 in reference)
GoM: East	13	[19] (Fig 2 in reference)
GoM: Eugene	14	[19] (Fig 2 in reference)
GoM: Ewing	23	[19] (Fig 2 in reference)
GoM: Galveston	2	[19] (Fig 2 in reference)
GoM: Garen	6	[19] (Fig 2 in reference)
GoM: Green	15	[19] (Fig 2 in reference)
GoM: Haredn	7	[19] (Fig 2 in reference)
GoM: High	13	[19] (Fig 2 in reference)
GoM: Main	9	[19] (Fig 2 in reference)
GoM: Marsh	12	[19] (Fig 2 in reference)
GoM: Marsh	10	[19] (Fig 2 in reference)
GoM: Misiss	30	[19] (Fig 2 in reference)
GoM: Mobile	3	[19] (Fig 2 in reference)
GoM: Mustang	11	[19] (Fig 2 in reference)
GoM: Padre	6	[19] (Fig 2 in reference)
GoM: Port	9	[19] (Fig 2 in reference)
GoM: Sabine	9	[19] (Fig 2 in reference)
GoM: Shi	10	[19] (Fig 2 in reference)
GoM: Sout	20	[19] (Fig 2 in reference)
GoM: South	13	[19] (Fig 2 in reference)
GoM: Vermi	7	[19] (Fig 2 in reference)
GoM: Viosca	5	[19] (Fig 2 in reference)
GoM: West	13	[19] (Fig 2 in reference)

Norway	20	[108] in [38]
Norway	25	[109] in [38]
Norway	18	[110] in [38]
Norway	38	[45] in [38]
UK CS	10	"Burton (2005)" in [38]
USA: GoM	60	[111]
Offshore Summary:	***Min: 2%; Max: 60%; Average: 14.5 ± 11.5% (1 s.d.)***	

Supplementary Table 4 – continued from previous page

Supplementary Table 5

Mass flow rates of continuous leakage reported on hydrocarbon wells in the literature.

**Context**	**Quoted leak rate**	**Normalised leak rate (t CO_2, STP)**
Industry (American Gas Association) survey of gas storage wells[35]	6.1 % of wells leaked. Of those that report leak rates:<br>60.74% leaked < 5,000 cf / d<br>28.59% leaked 5-25,000 cf/d<br>2.96% leaked 25-100,000 cf/d<br>0.74% leaked 100-250,000 cf/d<br>6.67% leaked >250,000 cf/d	3.7% leaked < 102 t/ year<br>1.7% leaked 102- 230 t/year<br>0.2% leaked 230-508 t/year<br>0.05% leaked 230 -5076 t/year<br>0.4% leaked >5076 t/year
SCP on outer casing strings of an offshore production well[19]	5 mcf / day	102 t / year
Methane leakage in surface casing and soil around wells in the Lloydminster area, Canada[36]	Leakage from surface casing vent: 0.01-200 m^3/d. Varies over time.<br><br>Soil leakage: most less than 0.1 m^3/d. Max 60 m^3/d. Soil leakage reduced due to bacterial oxidation of methane.	Up to 186 t / y (combined soil and casing leak)
Leak rate requiring immediate remediation in Alberta[29]	> 300 m^3/d	215 t / year

.

Supplementary Table 6

Blowout frequency data reported in the literature (Table extends over 2 pages)

Activity / Context	Area	Quoted data	Normalised data
UGS[112]	Worldwide	51 leakage events in 566 UGS sites in depleted hydrocarbon fields or aquifers, between the 1950s and 2005.	0.09 events per facility, and 0.0018 events per facility per year assuming 50 years of operation.
UGS[113]	Worldwide	All UGS: 10^{-5} events / well / year	All UGS: 10^{-5} events / well / year
		UGS in depleted hydrocarbon fields: 5.8-8.3 $\times 10^{-6}$ events / well / year	UGS in depleted hydrocarbon fields: 5.8-8.3 $\times 10^{-6}$ events / well / year
UGS[114]	Worldwide	20,271 UGS years and 791,547 well operation years since the 1970s. Major incident frequency of 8.39 $\times 10^{-4}$ events per site per year and 2.02 $\times$ 10^{-5} events per well per year.	8.39 $\times 10^{-4}$ events per site per year and 2.02 $\times$ 10^{-5} events per well per year.
Shale Gas[38]	Marcellus Field, Pennsylvania, USA	Over 2005-2013, 6.26% of 8030 wells documented well integrity failure. 1.27% resulted in a leak to the surface.	0.001588 leaks to surface / well / year
Shale Gas[107]	Marcellus Field, Pennsylvania, USA	6 major gas loss events, over 3533 wells, between Jan 2008 and August 2011,	4.63 $\times$ 10^{-4} major events / well / year
Shale Gas[106]	Marcellus Field, Pennsylvania, USA	2008 – March 2013, 16 out of 6466 wells were issued with a notice of violation for leakage.	0.000471 events / well / year
Onshore Hydrocarbon Production[115]	Ohio, USA	1983-2007:<br>1,595,978 production well years with 12 incidents.<br>20,374 wells plugged and abandoned with 5 incidents during abandonment.	7.52 x 10^{-6} incidents / well year<br>0.025% of abandonment procedures result in leakage incidents.
Onshore Hydrocarbon Production[115]	Texas, USA	1993-2008:<br>3,682,636 production well years with 6 incidents.<br>140,818 wells plugged and abandoned with 1 incident during abandonment.	1.63 x 10^{-6} incidents / well year<br>0.0007% of abandonment procedures result in leakage incidents.
Onshore Hydrocarbon Production[40]	Texas, USA	From 1998-2011:<br>District 3: 0.173% of 10,968 production wells blowout<br>District 8: 0.006% of 48, 897 production wells blowout.<br>District 8a: 0.049% of 22,622 production wells blowout.	District 3: 0.000133077 blowouts / well / year<br>District 8: 4.62 x 10^{-6} blowouts / well / year<br>District 8a: 3.769 x 10^{-5} blowouts / well / year
Onshore Hydrocarbon Production[103]	Pembina Field, Alberta, Canada	9860 cased wells drilled since the 1950s. 1.3% exhibit casing failure.	Assuming 55 years of activity, 0.000236 incidents f casing failure per well per year.

Activity / Context	Area	Quoted data	Normalised data
Onshore Hydrocarbon Production[103]	Zama Field, Alberta, Canada	607 cased wells drilled since the mid-1960s. 6% exhibit casing failure.	Assuming 40 years of activity, 0.0013 incidents of casing failure per well per year.
Onshore Hydrocarbon Production[39]	Alberta, Canada	1975-1990, 87944 wells drilled, 96 blowouts = 1.1 x 10^{-3} events per well	7.28 x 10^{-5} blowouts per well per year.
Offshore Hydrocarbon Production[37]	Gulf of Mexico, USA	0.00017 workover blowouts / w / y<br>0.00005 production blowouts / w / y<br>0.000028 wireline blowouts / w / y	0.000248 non-drilling blowouts / w / y
Offshore Hydrocarbon Production[37]	North Sea	0.00006 workover blowouts / w / y<br>0.00006 production blowouts / w / y	0.00012 non-drilling blowouts / w / y
Offshore Hydrocarbon Production[45]	Norwegian Continental Shelf (NCS), North Sea,	2008: 24% of 1677 wells had integrity issues, 1% possibly leaked.<br>2009: 24% of 1712 wells had integrity issues, 1% possibly leaked.<br>2010: 26% of 1741 wells had integrity issues, 0.3% possibly leaked.	39 possible leaks across 5130 well years<br>0.0076 possible leaks per well year
Offshore Hydrocarbon Production[43,44]	UK sector, North Sea	Total well years active between 1992 and 2014: 44755 (23036 gas wells).<br>Hydrocarbon release incidents associated with well operations 1992-2014: 2572 incidents (1597 on gas wells).	All well types: 0.0575 releases per well year<br>Gas wells: 0.0693 releases per well year.<br>Note: Most releases << 2000 m^3 ≡ 3.9 t CO_2.
Offshore Hydrocarbon Production[39]	GoM & North Sea (SINTEF database)	Production: 10 incidents / 211,142 well years.	4.736 x 10^{-5} production incidents per well year.
Offshore Hydrocarbon Production[39]	Scandpower blowout analysis (North Sea Standard)	Production: 14 incidents / 177,474 well years.	7.888 x 10^{-5} incidents per well year
CO_2 injection wells[33]	Alberta, Canada	14 wells drilled between 1981 and 1994 experienced 1.6 failures / well.<br>17 wells drilled between 1994 and 2008 experienced 0.06 failures per well.<br>Of all CO_2 well failures, only 1 resulted in leakage of fluid to the surface	For all wells: 0.04 failures / well / y<br>0.0012 leak events / well / y<br>For newer wells: 0.06 failures / well / y
Acid gas injection[116]	Western Canada	222 well years between 1989 and 2002. 1 blowout.	0.0045 incidents per well year.

Activity / Context	Area	Quoted data	Normalised data
Onshore injection for EOR[40]	Texas, USA	From 1998-2011:<br>District 3: 0% of 35 injection wells blowout.<br>District 8: 0.02% of 978 injection wells blowout.<br>District 8a: 0% of 1016 injection wells blowout.	District 8: 1.5 x 10^{-5} blowouts / well / year

Supplementary Figure 13: Blowout frequencies

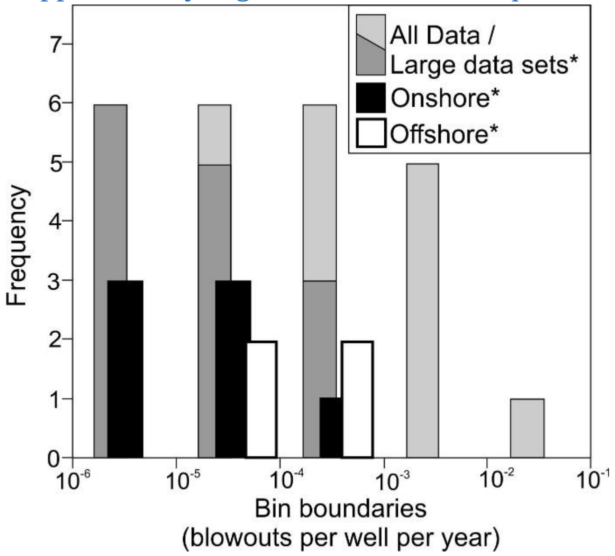

Histogram of blowout frequencies; data from Supplementary Table 6. * = large data sets only; for studies where well numbers are specified, this is >10,000 wells per study for production wells. The Onshore large data set also includes the largest injection well study, which involved 978 injection wells.

Supplementary Table 7

Leakage details of various documented blowouts with volume-equivalent tonnage of CO_2 leaked. Where blowouts continue for more than a year, the yearly leak rate is also presented. (Table extends over 3 pages)

Context	Quoted leak data	Normalised leak data
CO_2 production well blowout and subsequent leak in Bečej field, Serbia[117]	A blowout in 1968 lasted 209 days before well collapse killed the blowout. Continued leakage over the next 39 years was evident from a drop in reservoir pressure of ~ 1 bar / year, when the field was not being produced. Estimated volume of CO_2 leaked is ten times that of production volumes (35 e6 m^3 /y)	350,000,000 m^3 $year^{-1}$ **= 26,812,500 t CO_2 leaked (678,500 t CO_2 $year^{-1}$)**
CO_2 production well blowout during drilling, Sheep Mountain, Wyoming[118]	Blowout from March 17 – April 3 1982, with estimated flow rates of up to 5.6 million m^3 CO_2 / day	1,800,000 m^3 leaked **= 198,000 t CO_2**
EOR CO_2 well blowout[119]	40 mmcf of CO_2 over 4 days	1,132,674 m^3 **=2,225 t CO_2**
UGS in the Leidy depleted field, Pennsylvania[112]	Gas migrated along 5 wells at 850 m^3 / d, and later another 13 wells, causing overpressure in overlying sandstones and eventual blowout. The 6 week blowout vented an estimated 4 bcf (113.3 × 10^6 m^3).	For simplicity, gas loss is divided between the 18 implicated wells over 6 weeks: = 6,300,000 m^3 $well^{-1}$ ≡ **12,375 t CO_2**
General UGS[113]	~1×10^{-5} events per well per year for well failures resulting in a natural gas discharge rate from depleted oil and gas fields of 240 kg/s.	Assuming a pure methane composition = 2.9×10^7 m^3 day^{-1} **≡ 57,024 t CO_2 day^{-1}** Durations unknown
Kalle, Germany, leakage out of underground storage quantified using timelapse seismic[120]	60,000 m^3/d between 1995 and 2000. Leakage pathway unconfirmed but suspected to be related to two injection / production wells.	Total of ~ 109,500,000 m^3 21,900,000 m^3 $year^{-1}$ **≡ 215,089 t CO_2 (43,018 t CO_2 $year^{-1}$)**
Aliso Canyon, California, UGS (converted depleted oil field) blowout, 2015 [95]	95% methane and 4% ethane natural gas mixture leaked via 1 well. Estimated total of 97,100 t methane leaked between 23rd October 2015 and 18th February 2016.	Total 143,000,000 m^3 (5.7% of storage capacity) leaked over 119 days. **≡ 280,893 t CO_2**
Gas production blowout,Ontario, Canada[112]	1.950 mcm (69 mcf) of gas vented over 80 hours	1950 m^3 ≡ **4 t CO_2**
Gas production blowout, California, 2003[121]	500 mcf gas over 23 hours	14,158 m^3, **≡ 27.8 t CO_2**
Gas production blowout, California, 2003[121]	< 1 mmcf / d of gas and water over 22 hrs 40 mins	28317 m^3, **≡ 56 t CO_2**
Gas exploration blowout, North Sea, 22/4b Nov 1990[122]	100 million L gas per day at STP[114] in September 2011. Shallow blowout, couldn't be remediated for 21 years.	36,500,000 m^3 **≡ 1,505,700 t CO_2 (71,700 t CO_2 $year^{-1}$)**
Gas production blowout, Gulf of Mexico[19]	600 MMSCF of gas and 3200 Bbl of condensate leaked during 46 day blowout following 2 years of observed SCP.	17 million m^3 of material leaked during the blowout, ≡ **33,374 t CO_2**
Gas production blowout, California, 1996[121]	500 cf gas over 3 hours	14 m^3, **≡ 0.03 t CO_2**

Context	Quoted leak data	Normalised leak data
Gas drilling blowout, California, 1998[121]	30 mmcf gas and 25,000 bbl water / day over 4383 hours	Total 853,505 m^3, **≡ 1,677 t CO_2**
Gas production blowout, Taiwan, 1996[123]	4.11 e8 scm of gas lost over 305 days	**≡ 807,321 t CO_2**
UK Sector North Sea, release incidents 1992-2014[44]	Max vol leaked:<br>1,414,026 kg gas over 35 minutes<br>Min Vol leaked:<br>0.00007 kg gas over 0.5 minutes	1.68 e6 m^3 ≡ **3,889 t CO_2**<br><br>9.8 x 10^{-5} m^3 ≡ **1.9 x 10^{-7} t CO_2**
Hydrocarbon production blowout, California, 1992, workover[121]	2.2mmcf gas, 90 bbl oil and 1650 bbl water over 40 hours	Total of 62,575 m^3 **≡ 123 t CO_2**
Hydrocarbon production blowout, California, 1993 workover prep[121]	100 bbl oil, gas and water over 19.75 hours	Total of 16 m^3, **≡ 0.03 t CO_2**
Hydrocarbon production blowout, California, 1994[121]	Up to 20 mmcf gas and 400 bbl oil per day over 129 hours	Total of 566,401 m^3 / d<br>Total vol. leaked = 3,044,405 m^3<br>**≡ 5980 t CO_2**
Hydrocarbon production blowout, California, 1997[121]	15 mmcf gas, 236 bbl water and oil over 37 hours	Total of 24,790 m^3, **≡ 834 t CO_2**
Hydrocarbon production blowout, California, 1995[121]	50 bbl oil, 50 bbl water and 4 mmcf gas over 6.5 hours	Total of 113,283 m^3, **≡ 223 t CO_2**
Hydrocarbon production blowout, California, 1992[121]	>50 bbl oil and gas over 1 hour	8 m^3, **≡ 0.02 t CO_2**
Hydrocarbon production, BP GoM Deepwater Horizon / Macondo blowout[124]	Up to 84,000 barrels of oil per day, over 84 days. Each barrel of oil exsolves up to 84 m^3 of gas	5.94 e8 m^3 oil and gas total **≡ 1,166,458 t CO_2**
Oil production, Bravo blowout, North Sea, 1977[37]	20,000 m^3 of oil over 8 days	≡ **39 t CO_2**
Oil production blowout, California, 1991, steam injection[121]	8200 bbl of oil and water over 48 hours	1312 m^3, **≡ 2.58 t CO_2**
Oil production blowout, California, 1996 steam injection[121]	<1 bbl oil over 1.5 hours	0.16 m^3 **≡ 0.0003 t CO_2**
Oil production blowout, California, 1997 Steam injection[121]	100 bbl steam, oil and water over 1 day	16 m^3, **≡ 0.03 t CO_2**
Oil production blowout, California, 1992, perforation breakdown[121]	25 bbl oil and steam over 24 hours	4 m^3, **≡ 0.008 t CO_2**
Oil production blowout, California, 1993[121]	20 bbl oil / steam over 120 hours	3.2 m^3, **≡ 0.006 t CO_2**
Oil production blowout, California, 1991[121]	7 bbl over 2 hours	1.1 m^3, **≡ 0.002 t CO_2**
Oil production blowout, California, 1996 reworking[121]	100 bbl water and oil over 0.5 hours	16 m^3, **≡ 0.03 t CO_2**
Oil production blowout, California, 1996 shut in[121]	62 bbl water and oil over 12.5 hours	10 m^3, **≡ 0.02 t CO_2**

Context	Quoted leak data	Normalised leak data
Oil production blowout, California, 1997 drilling[121]	300 bbl water and oil over 6.5 hours	48 m^3, ≡ **0.09 t CO_2**
Oil production blowout, California, 1997 production[121]	0.1 bbl oil over 1.5 hours	0.016 m^3, ≡ **0.00003 t CO_2**
Oil production blowout, California, 1998 production[121]	15 bbl oil over 1 day	2.4 m^3, ≡ **0.005 t CO_2**
Oil production blowout, California, 2000 workover[121]	3 bbl oil / water / steam over 4.5 hours	0.48 m^3, ≡ **0.001 t CO_2**
Oil production blowout, California, 2000[121]	10 bbl water and oil over 10 hours 25 mins	1.6 m^3, ≡ **0.003 t CO_2**
Steam / water injection blowout, California, 1994 post steam injection[121]	2 bbl steam and water over 10 mins	0.3 m^3, ≡ **0.0006 t CO_2**
Steam / water injection blowout, California, 1993 maintenance[121]	200 bbs steam over 3 hours	32 m^3, ≡ **0.06 t CO_2**
Steam / water injection blowout, California, 1994 recently abandoned well[121]	100 bbs steam and sand over 5 hours	16 m^3, ≡ **0.03 t CO_2**
Steam / water injection blowout, California, 1992, cyclical injection[121]	300 bbl water over 12 hours	48 m^3, ≡ **0.09 t CO_2**
Steam / water injection blowout, California, 1992, steam cycling[121]	<1 bbl mud	0.16 m^3 ≡ **0.0003 t CO_2**
Steam / water injection blowout, California, 1993. pump change[121]	2 bbl water over 5 min	0.3 m^3, ≡ **0.0006 t CO_2**

Supplementary Table 7 continued from previous pages

Supplementary Table 8

Modelled CO_2 leakage rates along abandoned wellbores of varying permeability. [Letters] indicate data labels in Supplementary Figure 9.

Ref.	Relevant Parameters	Quoted leakage details	Normalised details
125	Effective permeabilities of the leaky well bore range from ~1.5 x 10^{-4} to 2 x 10^{3} mD. [a]	Leak rates between ~1 x 10^{-2} to 1 x 10^{4} mg/m^{2}/s for a reservoir at hydrostatic pressure, assuming leakage is buoyancy driven. Values ~4 times higher if overpressure is involved.	Assuming a 3 m leakage radius and underpressure: 0.001 to 946 t CO_2 year^{-1} well^{-1}. Overpressure: 0.004 to 1,892 t CO_2 year^{-1} well^{-1}
126	Well permeabilities of 10 μD to 10 mD. Buoyancy driven leakage. [b]	Max 0.1 t / y / w	0.1 t CO_2 year^{-1} well^{-1}
127	Well permeabilities of 0.001 to 1000 mD Wellbore length of 2100 m 400 bar (2 x hydrostatic pressure) at base of well. [c]	<10 out of 1296 scenarios leaked more than 30 t per 1000 years	0.03 t CO_2 year^{-1} well^{-1}
128	Bad cement permeability = 10 mD Fractured cement permeability = 1000 mD 100 Monte Carlo simulations varying reservoir and well conditions. [d]	Max leak rate for bad cement: ~0.014 kg / d Max leak rate for fractured cement: ~1.15 kg / d	Bad cement: 0.005 t CO_2 year^{-1} well^{-1} Fractured cement: 0.4 t CO_2 year^{-1} well^{-1}
129	Fractured cement permeabilities of 3 (±1) x 10^{-15} m^{2} (=3 ± 1 mD). 50 years injection at 50 kg/s into a reservoir 3 km deep at 30 MPa. 1000 simulations. [e]	8 e10 kg injected Mean leak rate = 6e4 kg / 50 years (<<0.01% / year)	1.2 t CO_2 year^{-1}
130	1525 m deep well. Model injected 12.7 kg CO2 / s (0.4 Mt / y) for 5 years. Well modelled with permeability of 10^{-11} (10 D). [f]	Leak rate varied from 0.2 kg/s to 1.1 kg/s at an abandoned well 100 m away from the injection well. Leakage becomes negligible if the wells are > 500 m apart.	6310 to 34,700 t CO_2 year^{-1}
131	1 injection well (1Mt / year for 100 years); 22 abandoned wells. Good cement permeability: 1 x 10^{-17} m^{2} (0.01 mD). Injection well model location varied. Bad cement permeability: 1 x 10^{-11} m^{2} (10,000 mD) Inject 1 Mt per year for 100 years [g]	Up to ~0.25% of injected CO2 (100 Mt) leaked over 100 years. Leak rate significantly decreased (to 0.13%) by moving the injection well away from the abandoned wells.	114 t CO_2 year^{-1} well^{-1}
132	Well permeabilities of 0.01 to 10,000 mD Simulates 50 years of injection. Minimum of 6 abandoned wells in contact with CO_2 plume. [h]	Log_{10}Fraction CO2 leaked = -2 to -7 (≡1% to 1 x 10^{-5}%) over 50 years	3.3 x 10^{-8}% to 0.003% of injected CO_2 well^{-1} year^{-1}

Ref.	Relevant Parameters	Quoted leakage details	Normalised details
133	Intact cement permeability = 10 μD.<br>Degraded cement permeability = 1mD to 100 D<br>Models scenarios of 1146 wells with variably degraded cement.<br>12.3 Mt CO2 injected per year for 50 years; Total 615 Mt CO2 injected. [i]	All simulations give leak rates <1% / y for the entire field.	5366 t CO_2 $year^{-1}$ $well^{-1}$

Supplementary Table 9

Experimentally derived well permeabilities. [Letters] indicate data labels in Supplementary Figure 9.

**Study details:**	**Min Permeability (mD)**	**Max permeability (mD)**
Calculated via gas flow on abandoned wells, assumes infinite supply of gas. Largest values used. [52] [j]	1	100
Calculated via gas flow on SCP on ~300 active wells[126,134]. [k]	0.01	1000
Calculated via gas flow on SCVF on active wells[126,134] [l]	0.0001	10
Vertical interference testing along ~15m of active wellbores[135] [m]	1	100
Measurements on cement cores from a 30 year old CO2-EOR well[20] [n]	0.08	18.8
Measurements on corroded cement from a 30 year old CO2 production well[25] [o]	0.5	1
Experimentally corroded steel-cement interface[21] [p]	520	670

Supplementary Table 10

Published experimental values for residual saturation trapping (R, in % of the CO_2 trapped). Modified from [97]. From these 43 residual trapping values we calculate a mean and standard deviation of 58.45 ± 18.96 %. (1 page)

Lithology	Reference	Location	Formation name	R (%)
Sandstone	[136]	Alberta, Canada	Viking Fm #2	51.5
			Cardium Fm #1	12.8
			Cardium Fm #2	43.6
	[137]	Alberta, Canada	Viking Fm #3	55.9
			Clearwater Fm	22.1
			Ellerslie Fm #2	68.1
			Rock Creek Fm	91.6
			Halfway Fm	86
			Belloy	81.6
			Graminia Fm	68.6
			Gilwood Fm	82.7
			Basal Cambrian Fm #2	54.3
			Basal Cambrian Fm #3	79
			Basal Cambrian Fm #4	77.1
			Basal Cambrian Fm #5	71.6
			Deadwood Fm #1	74.9
			Deadwood Fm #2	71.3
			Deadwood Fm #3	68.8
			Granite Wash	53.6
	[138]	Japan	Tako Sandstone	65.1
	[139]	OH, USA	Berea	67.1
	[140]	OH, USA	Berea	41.2
	[141]	OH, USA	Berea	56.4
		Australia	Paaratte	55.9
		IL, USA	Mt. Simon	38.9
		MS, USA	Tuscaloosa	67.4
	[142]	Elgin, Scotland	Clashach	61.3
		Lincolnshire, England	Sherwood	63.9
	[143]	Goldeneye Field, North Sea	Captain #1	56.7
			Captain #2	41.4
Simulation	[144]	OH, USA	Berea	58
	[145]	TX, USA	Frio Fm	20
	[146]	Browse Basin, Australia	Carbine	37.5
Shale	[136]	Alberta, Canada	Calmar Fm	70.7
			Colorado Gp	88.4
Carbonate	[136]	Alberta, Canada	Nisku Fm #2	42.9
	[147]	Alberta, Canada	Wabamun #3	30.4
			Nisku Fm #3	34.3
			Grosmont	74.2
			Morinville Leduc	27.9
			Redwater Leduc	62.1
			Cooking Lake #2	66.4
			Slave Point	56.4
			Winnipegosis	52.6

Supplementary References

1. IEA. *Technology Roadmap: Carbon Capture and Storage*. 63 (IEA, 2013).
2. Riding, J. B. & Rochelle, C. A. Subsurface characterization and geological monitoring of the CO2 injection operation at Weyburn, Saskatchewan, Canada. *Geological Society, London, Special Publications* **313,** 227–256 (2009).
3. Gluyas, J. G. & Hichens, H. M. Appendix 1. in *United Kingdom Oil and Gas Fields Commemorative Millenium Volume* **20,** 949–977 (2003).
4. PCOR. *PCOR Atlas, 4th Edition, Revised*. (Energy and Environment Reseach Centre, 2013).
5. NETL. *Carbon Storage Atlas: 5th Edition*. (US Department of Energy, 2015).
6. Hovorka, S. D. *et al.* Monitoring a large volume CO2 injection: Year two results from SECARB project at Denbury's Cranfield, Mississippi, USA. *Energy Procedia* **4,** 3478–3485 (2011).
7. Eiken, O. *et al.* Lessons learned from 14 years of CCS operations: Sleipner, In Salah and Snøhvit. *Energy Procedia* **4,** 5541–5548 (2011).
8. White, D. *et al.* Monitoring Results after 36 Ktonnes of Deep CO 2 Injection at the Aquistore CO 2 Storage Site, Saskatchewan, Canada. *Energy Procedia* **114,** 4056–4061 (2017).
9. Tucker, O., Gray, L., Maas, W. & O'Brien, S. Quest Commercial Scale CCS – The First Year. in (International Petroleum Technology Conference, 2016). doi:10.2523/IPTC-18666-MS
10. Office of Fossil Energy. Archer Daniels Midland Company. (2017). Available at: https://energy.gov/fe/archer-daniels-midland-company. (Accessed: 18th October 2017)
11. U.S. E.P.A. U.S. EPA Approves Carbon Sequestration Permit in Decatur, Illinois. (2014). Available at: https://yosemite.epa.gov/opa/admpress.nsf/a5792a626c8dac098525735900400c2d/afbc8abba5c91e3685257d5f0050ac84!OpenDocument. (Accessed: 18th October 2017)

12. Ringrose, P. S. *et al.* The In Salah CO2 Storage Project: Lessons Learned and Knowledge Transfer. *Energy Procedia* **37,** 6226–6236 (2013).

13. Celia, M., Bachu, S., Nordbotten, J., Gasda, S. & Dahle, H. Quantitative estimation of CO2 leakage from geological storageAnalytical models, numerical models, and data needs. in *Greenhouse Gas Control Technologies 7* **I,** 663–671 (Elsevier, 2005).

14. Bachu, S. & Celia, M. A. Assessing the potential for CO_2 leakage, particularly through wells, from geological storage sites. in *Geophysical Monograph Series* (eds. McPherson, B. J. & Sundquist, E. T.) **183,** 203–216 (American Geophysical Union, 2009).

15. Bai, M., Zhang, Z. & Fu, X. A review on well integrity issues for CO_2 geological storage and enhanced gas recovery. *Renewable and Sustainable Energy Reviews* **59,** 920–926 (2016).

16. King, G. E. & King, D. E. Environmental Risk Arising From Well-Construction Failure--Differences Between Barrier and Well Failure, and Estimates of Failure Frequency Across Common Well Types, Locations, and Well Age. *SPE Production & Operations* **28,** 323–344 (2013).

17. Kermani, M. B. & Morshed, A. Carbon Dioxide Corrosion in Oil and Gas Production—A Compendium. *Corrosion* **59,** 659–683 (2003).

18. Skinner, L. CO2 well blowouts: an emerging problem. *World Oil* **224,** (2003).

19. Bourgoyne, A. T., Scott, S. L. & Regg, J. B. Sustained Casing Pressure in Offshore Producing Wells. in (Offshore Technology Conference, 1999). doi:10.4043/11029-MS

20. Carey, J. W. *et al.* Analysis and performance of oil well cement with 30 years of CO_2 exposure from the SACROC Unit, West Texas, USA. *International Journal of Greenhouse Gas Control* **1,** 75–85 (2007).

21. Carey, W. J., Robert, S., Reid, G., Zhang, J. & Crow, W. Experimental investigation of wellbore integrity and CO2–brine flow along the casing–cement microannulus. *International Journal of Greenhouse Gas Control* **4,** 272–282 (2010).

22. Carroll, S. *et al.* Review: Role of chemistry, mechanics, and transport on well integrity in CO_2 storage environments. *International Journal of Greenhouse Gas Control* **49,** 149–160 (2016).

23. Choi, Y.-S., Young, D., Nešić, S. & Gray, L. G. S. Wellbore integrity and corrosion of carbon steel in CO_2 geologic storage environments: A literature review. *International Journal of Greenhouse Gas Control* **16,** S70–S77 (2013).

24. Crow, W., Carey, J. W., Gasda, S., Brian Williams, D. & Celia, M. Wellbore integrity analysis of a natural CO2 producer. *International Journal of Greenhouse Gas Control* **4,** 186–197 (2010).

25. Crow, W., Brian Williams, D., William Carey, J., Celia, M. & Gasda, S. Wellbore integrity analysis of a natural CO2 producer. *Energy Procedia* **1,** 3561–3569 (2009).

26. Nešić, S. Key issues related to modelling of internal corrosion of oil and gas pipelines – A review. *Corrosion Science* **49,** 4308–4338 (2007).

27. Regnault, O., Lagneau, V. & Schneider, H. Experimental measurement of portlandite carbonation kinetics with supercritical CO2. *Chemical Geology* **265,** 113–121 (2009).

28. Rimmelé, G., Barlet-Gouédard, V., Porcherie, O., Goffé, B. & Brunet, F. Heterogeneous porosity distribution in Portland cement exposed to CO2-rich fluids. *Cement and Concrete Research* **38,** 1038–1048 (2008).

29. Watson, T. L. & Bachu, S. Evaluation of the Potential for Gas and CO2 Leakage Along Wellbores. *SPE Drilling & Completion* **24,** 115–126 (2009).

30. Wigand, M., Kaszuba, J. P., Carey, J. W. & Hollis, W. K. Geochemical effects of CO2 sequestration on fractured wellbore cement at the cement/caprock interface. *Chemical Geology* **265,** 122–133 (2009).

31. Loizzo, M. *et al.* Quantifying the Risk of CO2 Leakage Through Wellbores. *SPE Drilling & Completion* **26,** 324–331 (2011).

32. Gasda, S. E., Bachu, S. & Celia, M. A. Spatial characterization of the location of potentially leaky wells penetrating a deep saline aquifer in a mature sedimentary basin. *Environmental Geology* **46,** 707–720 (2004).

33. Bachu, S. & Watson, T. L. Review of failures for wells used for CO2 and acid gas injection in Alberta, Canada. *Energy Procedia* **1,** 3531–3537 (2009).
34. Aly, M. *et al.* Geochemical Applications for Identifying the Source of Hydrocarbons in Well Annuli. in (International Petroleum Technology Conference, 2015). doi:10.2523/IPTC-18309-MS
35. Marlow, R. S. Cement Bonding Characteristics in Gas Wells. *Journal of Petroleum Technology* **41,** 1146–1153 (1989).
36. Erno, B. & Schmitz, R. Measurements of Soil Gas Migration Around Oil And Gas Wells In the Lloydminster Area. *Journal of Canadian Petroleum Technology* **35,** (1996).
37. Holand, P. *Offshore Blowouts: Causes and Control*. (Elsevier, 1997).
38. Davies, R. J. *et al.* Oil and gas wells and their integrity: Implications for shale and unconventional resource exploitation. *Marine and Petroleum Geology* **56,** 239–254 (2014).
39. OGP. *Blowout Frequencies*. (International Association of Oil and Gas Producers, 2010).
40. Porse, S. L., Wade, S. & Hovorka, S. D. Can We Treat CO2 Well Blowouts like Routine Plumbing Problems? A Study of the Incidence, Impact, and Perception of Loss of Well Control. *Energy Procedia* **63,** 7149–7161 (2014).
41. SINTEF. SINTEF Offshore Blowout Database webpage. (2013).
42. Calosa, W. J., Sadarta, B. & Ronaldi, R. Well Integrity Issues in Malacca Strait Contract Area. in (Society of Petroleum Engineers, 2010). doi:10.2118/129083-MS
43. HSE. *Offshore Hydrocarbon Population Data 1992 - 2015*. (HSE, 2015).
44. HSE. *Offshore Hydrocarbon Releases 1992-2015*. (2015).
45. Vignes, B. Contribution to Well Integrity and Increased Focus on Well Barriers from a Life Cycle Aspect. (University of Stavanger, 2011).
46. Danenberger, E. P. *Outer Continental Shelf Oil and Gas Blowouts*. (1980).
47. Lindeberg, E., Bergmo, P., Torsæter, M. & Grimstad, A.-A. Aliso Canyon Leakage as an Analogue for Worst Case CO 2 Leakage and Quantification of Acceptable Storage Loss. *Energy Procedia* **114,** 4279–4286 (2017).

48. Celia, M. A., Nordbotten, J. M., Bachu, S., Dobossy, M. & Court, B. Risk of Leakage versus Depth of Injection in Geological Storage. *Energy Procedia* **1,** 2573–2580 (2009).

49. Kang, M. *et al.* Identification and characterization of high methane-emitting abandoned oil and gas wells. *Proceedings of the National Academy of Sciences* 201605913 (2016). doi:10.1073/pnas.1605913113

50. Hammack, R., Veloski, G. & Sams, J. Rapid Methods for Locating Existing Well Penetrations in Unconventional Well Development Areas of Pennsylvania. in (Society of Petroleum Engineers, 2015). doi:10.2118/178558-MS

51. Hammack, R. W., Veloski, G., Hodges, D. G. & White, C. M. *Methods for Finding Legacy Wells in Large Areas*. 28 (U.S. Department of Energy, National Energy Technology Laboratory, 2016).

52. Kang, M., Baik, E., Miller, A. R., Bandilla, K. W. & Celia, M. A. Effective Permeabilities of Abandoned Oil and Gas Wells: Analysis of Data from Pennsylvania. *Environmental Science & Technology* **49,** 4757–4764 (2015).

53. Nordbotten, J. M., Celia, M. A. & Bachu, S. Injection and Storage of CO2 in Deep Saline Aquifers: Analytical Solution for CO2 Plume Evolution During Injection. *Transport in Porous Media* **58,** 339–360 (2005).

54. Nordbotten, J. M., Celia, M. A. & Bachu, S. Analytical solutions for leakage rates through abandoned wells: ANALYTICAL SOLUTIONS FOR LEAKAGE RATES. *Water Resources Research* **40,** n/a-n/a (2004).

55. Kang, M. *et al.* Direct measurements of methane emissions from abandoned oil and gas wells in Pennsylvania. *Proceedings of the National Academy of Sciences* **111,** 18173–18177 (2014).

56. Jordan, P. & Carey, J. W. Steam blowouts in California Oil and Gas District 4: Comparison of the roles of initial defects versus well aging and implications for well blowouts in geologic carbon storage projects. *International Journal of Greenhouse Gas Control* **51,** 36–47 (2016).

57. Metz, B., Davidson, O., de Coninck, H., Loos, M. & Meyer, L. *IPCC Special Report on Carbon Capture and Storage*. (Cambridge University Press, 2005).

58. Nicot, J.-P. A survey of oil and gas wells in the Texas Gulf Coast, USA, and implications for geological sequestration of CO2. *Environmental Geology* **57,** 1625–1638 (2009).

59. Miocic, J. M. *et al.* Controls on CO_2 storage security in natural reservoirs and implications for CO_2 storage site selection. *International Journal of Greenhouse Gas Control* **51,** 118–125 (2016).

60. Angeli, M., Soldal, M., Skurtveit, E. & Aker, E. Experimental percolation of supercritical CO2 through a caprock. *Energy Procedia* **1,** 3351–3358 (2009).

61. Busch, A., Amann-Hildenbrand, A., Bertier, P., Waschbuesch, M. & Krooss, B. M. The Significance of Caprock Sealing Integrity for CO2 Storage. in (Society of Petroleum Engineers, 2010). doi:10.2118/139588-MS

62. Busch, A. *et al.* Carbon dioxide storage potential of shales. *International Journal of Greenhouse Gas Control* **2,** 297–308 (2008).

63. Wollenweber, J. *et al.* Experimental investigation of the CO2 sealing efficiency of caprocks. *International Journal of Greenhouse Gas Control* **4,** 231–241 (2010).

64. Deming, D. Factors Necessary to Define a Pressure Seal. *AAPG Bulletin* **78,** (1994).

65. Liu, F. *et al.* CO2–brine–caprock interaction: Reactivity experiments on Eau Claire shale and a review of relevant literature. *International Journal of Greenhouse Gas Control* **7,** 153–167 (2012).

66. Zoback, M. D. & Gorelick, S. M. Earthquake triggering and large-scale geologic storage of carbon dioxide. *Proceedings of the National Academy of Sciences* **109,** 10164–10168 (2012).

67. Shi, J.-Q., Sinayuc, C., Durucan, S. & Korre, A. Assessment of carbon dioxide plume behaviour within the storage reservoir and the lower caprock around the KB-502 injection well at In Salah. *International Journal of Greenhouse Gas Control* **7,** 115–126 (2012).

68. Tsang, C.-F., Stephansson, O., Kautsky, F. & Jing, L. Coupled THM Processes in Geological Systems and the Decovalex Project. in *Elsevier Geo-Engineering Book Series* **2,** 3–16 (Elsevier, 2004).

69. Bjørlykke, K. Fluid flow in sedimentary basins. *Sedimentary Geology* **86,** 137–158 (1993).

70. Chadwick, A. *et al. Best Practice for the Storage of CO_2 in Saline Aquifers,*. 267pp (British Geological Survey, 2008).

71. Song, J. & Zhang, D. Comprehensive Review of Caprock-Sealing Mechanisms for Geologic Carbon Sequestration. *Environmental Science & Technology* **47,** 9–22 (2013).

72. Aydın, H., Hilton, D. R., Güleç, N. & Mutlu, H. Post-earthquake anomalies in He–CO_2 isotope and relative abundance systematics of thermal waters: The case of the 2011 Van earthquake, eastern Anatolia, Turkey. *Chemical Geology* **411,** 1–11 (2015).

73. Barry, P. H. *et al.* Helium and carbon isotope systematics of cold 'mazuku' CO2 vents and hydrothermal gases and fluids from Rungwe Volcanic Province, southern Tanzania. *Chemical Geology* **339,** 141–156 (2013).

74. Bond, C. E. *et al.* The physical characteristics of a CO_2 seeping fault: The implications of fracture permeability for carbon capture and storage integrity. *International Journal of Greenhouse Gas Control* **61,** 49–60 (2017).

75. Duffy, M., Kinnaman, F. S., Valentine, D. L., Keller, E. A. & Clark, J. F. Gaseous emission rates from natural petroleum seeps in the Upper Ojai Valley, California. *Environmental Geosciences* **14,** 197–207 (2007).

76. Karolytė, R., Serno, S., Johnson, G. & Gilfillan, S. M. V. The influence of oxygen isotope exchange between CO_2 and H_2O in natural CO_2-rich spring waters: Implications for geothermometry. *Applied Geochemistry* **84,** 173–186 (2017).

77. Keating, E., Newell, D., Dempsey, D. & Pawar, R. Insights into interconnections between the shallow and deep systems from a natural CO2 reservoir near Springerville, Arizona. *International Journal of Greenhouse Gas Control* **25,** 162–172 (2014).

78. Keating, E. H., Fessenden, J., Kanjorski, N., Koning, D. J. & Pawar, R. The impact of CO2 on shallow groundwater chemistry: observations at a natural analog site and implications for carbon sequestration. *Environmental Earth Sciences* **60,** 521–536 (2010).

79. Mutlu, H., Güleç, N., Hilton, D. R., Aydın, H. & Halldórsson, S. A. Spatial variations in gas and stable isotope compositions of thermal fluids around Lake Van: Implications for crust–mantle dynamics in eastern Turkey. *Chemical Geology* **300–301,** 165–176 (2012).

80. Watson, Z. T., Han, W. S., Keating, E. H., Jung, N.-H. & Lu, M. Eruption dynamics of CO2-driven cold-water geysers: Crystal, Tenmile geysers in Utah and Chimayó geyser in New Mexico. *Earth and Planetary Science Letters* **408,** 272–284 (2014).

81. Weber, D. *et al.* Macroseepage of methane and light alkanes at the La Brea tar pits in Los Angeles. *Journal of Atmospheric Chemistry* **74,** 339–356 (2017).

82. Lewicki, J. L. & Brantley, S. L. CO_2 degassing along the San Andreas Fault, Parkfield, California. *Geophysical Research Letters* **27,** 5–8 (2000).

83. Mörner, N.-A. & Etiope, G. Carbon degassing from the lithosphere. *Global and Planetary Change* **33,** 185–203 (2002).

84. The World Bank. Land area (sq. km). (2017). Available at: https://data.worldbank.org/indicator/AG.LND.TOTL.K2?name_desc=false. (Accessed: 17th October 2017)

85. Ármannsson, H., Fridriksson, T. & Kristjánsson, B. R. CO2 emissions from geothermal power plants and natural geothermal activity in Iceland. *Geothermics* **34,** 286–296 (2005).

86. Williams, S. N., Schaefer, S. J., Marta Lucia Calvache, V. & Lopez, D. Global carbon dioxide emission to the atmosphere by volcanoes. *Geochimica et Cosmochimica Acta* **56,** 1765–1770 (1992).

87. Coble, C. R., Murray, E. G. & Rice, D. R. *Earth Science*. (Prentice-Hall, 1987).

88. Etiope, G. Mud volcanoes and microseepage: the forgotten geophysical components of atmospheric methane budget. *Annals of Geophysics* (2005). doi:10.4401/ag-3175

89. White, S. P. *et al.* Evaluating the seal integrity of natural CO2 reservoirs of the Colorado Plateau. in *Proceedings of the 3rd National Conference on Carbon Sequestration* 32 (2004).

90. Raich, J. W. & Schlesinger, W. H. The global carbon dioxide flux in soil respiration and its relationship to vegetation and climate. *Tellus B* **44,** 81–99 (1992).

91. Klusman, R. W. Rate measurements and detection of gas microseepage to the atmosphere from an enhanced oil recovery/sequestration project, Rangely, Colorado, USA. *Applied Geochemistry* **18,** 1825–1838 (2003).

92. Zahasky, C. & Benson, S. M. Evaluation of hydraulic controls for leakage intervention in carbon storage reservoirs. *International Journal of Greenhouse Gas Control* **47,** 86–100 (2016).

93. Jordan, A. B., Stauffer, P. H., Harp, D., Carey, J. W. & Pawar, R. J. A response surface model to predict CO 2 and brine leakage along cemented wellbores. *International Journal of Greenhouse Gas Control* **33,** 27–39 (2015).

94. Réveillère, A., Rohmer, J. & Manceau, J.-C. Hydraulic barrier design and applicability for managing the risk of CO2 leakage from deep saline aquifers. *International Journal of Greenhouse Gas Control* **9,** 62–71 (2012).

95. Conley, S. *et al.* Methane emissions from the 2015 Aliso Canyon blowout in Los Angeles, CA. *Science* **351,** 1317–1320 (2016).

96. Samson, L. A. Gas Production And Decline Rates In the Province of Alberta. in (Petroleum Society of Canada, 1999). doi:10.2118/99-70

97. Burnside, N. M. & Naylor, M. Review and implications of relative permeability of CO2/brine systems and residual trapping of CO2. *International Journal of Greenhouse Gas Control* **23,** 1–11 (2014).

98. Kumar, A., Noh, M. H., Pope, G. A., Sepehrnoori, K. & Bryant, S. L. Simulating CO2 storage in deep saline aquifers. in *Carbon Dioxide Capture for Storage in Deep Geologic Formations* (ed. Benson, S. M.) **2,** 877–896 (Elsevier, 2005).

99. Gilfillan, S. M. V. *et al.* Solubility trapping in formation water as dominant CO2 sink in natural gas fields. *Nature* **458,** 614–618 (2009).

100. Lu, J., Wilkinson, M., Haszeldine, R. S. & Boyce, A. J. Carbonate cements in Miller field of the UK North Sea: a natural analog for mineral trapping in CO2 geological storage. *Environmental Earth Sciences* **62,** 507–517 (2011).

101. Xu, T., Apps, J. A. & Pruess, K. Reactive geochemical transport simulation to study mineral trapping for CO_2 disposal in deep arenaceous formations. *Journal of Geophysical Research: Solid Earth* **108,** (2003).

102. Sivakumar, V. C. B. & Janahi, I. A. Salvage of Casing Leak Wells on Artificial Lift in a Mature Oil Field. in (Society of Petroleum Engineers, 2004). doi:10.2118/88749-MS

103. Watson, T. L. & Bachu, S. Identification of Wells With High CO2-Leakage Potential in Mature Oil Fields Developed for CO2-Enhanced Oil Recovery. in (Society of Petroleum Engineers, 2008). doi:10.2118/112924-MS

104. Lan, Z., Xia, Y. & Zhang, C. The Repairing Technology of Driving Channel on Small Drifting-diameter's Casing Damage in Daqing Oilfield. in (Society of Petroleum Engineers, 2000). doi:10.2118/65099-MS

105. Ingraffea, A. *Fluid Migration Mechanisms Due to Faulty Well Design and/or Construction: an Overview and Recent Experiences in the Pennsylvania and Marcellus Play.* (2012).

106. Vidic, R. D., Brantley, S. L., Vandenbossche, J. M., Yoxtheimer, D. & Abad, J. D. Impact of Shale Gas Development on Regional Water Quality. *Science* **340,** 1235009–1235009 (2013).

107. Considine, T. J., Watson, R. W., Considine, N. B. & Martin, J. P. Environmental regulation and compliance of Marcellus Shale gas drilling. *Environmental Geosciences* **20,** 1–16 (2013).

108. Nilsen, L. H. Brønnintegritet I Statoil og på norsk sokkel. in *Published at the NPF 20‘ Kristiansand Conference [Online].* (2007).

109. Randhol, P. & Carlsen, I. M. Presentation Assessment of Sustained Well Integrity on the Norwegian Continental Shelf. in *SINTEF Petroleum Research [Online],* (2007).

110. Vignes, B. & Aadnøy, B. S. Well-Integrity Issues Offshore Norway. *SPE Production & Operations* **25,** 145–150 (2010).

111. Brufatto, C. *et al.* From mud to cement - building gas wells. *Oilfield Review* **Autumn 2003,** 62–76 (2003).

112. Evans, D. J. A review of underground fuel storage events and putting risk into perspective with other areas of the energy supply chain. *Geological Society, London, Special Publications* **313,** 173–216 (2009).

113. Keeley, D. *Failure rates for underground gas storage Significance for land use planning assessments*. 25 pp (Health and Safety Executive, 2008).

114. IEA GHG. *Safe Storage of CO2: Experience from the Natural Gas Storage Industry*. (International Energy Agency Greenhouse Gas R&D Programme, 2006).

115. Kell, S. *State Oil and Gas Agency Groundwater Investigations And their Role in Advancing Regulatory Reforms. A Two-State Review: Ohio and Texas*. (2011).

116. IEA GHG. *Acid Gas Injection: A Study of Existing Operations. Phase 1: Final Report*. 71 pp (IEA GHG, 2003).

117. Karas, D. *et al.* First Field Example of Remediation of Unwanted Migration from a Natural CO2 Reservoir: The Bečej Field, Serbia. *Energy Procedia* **86,** 69–78 (2016).

118. Lynch, R. D., McBride, E. J., Perkins, T. K. & Wiley, M. E. Dynamic Kill of an Uncontrolled CO2 Well. *Journal of Petroleum Technology* **37,** 1267–1275 (1985).

119. Duncan, I. J., Nicot, J.-P. & Choi, J.-W. Risk Assessment for future CO2 Sequestration Projects Based CO2 Enhanced Oil Recovery in the U.S. *Energy Procedia* **1,** 2037–2042 (2009).

120. Schuck, A., Rost, F., Czolbe, P. & M. Klafki. Time lapse seismic for the development of an underground gas storage. in (2002).

121. Jordan, P. D. & Benson, S. M. Well blowout rates and consequences in California Oil and Gas District 4 from 1991 to 2005: implications for geological storage of carbon dioxide. *Environmental Geology* **57,** 1103–1123 (2009).

122. Leifer, I. & Judd, A. The UK22/4b blowout 20 years on: Investigations of continuing methane emissions from sub-seabed to the atmosphere in a North Sea context. *Marine and Petroleum Geology* **68,** 706–717 (2015).

123. Hsieh, B.-Z., Chen, T.-L., Tseng, C.-C., Chilingar, G. V. & Lin, Z.-S. Case study of estimating gas loss from a producing well blowout. *Journal of Petroleum Science and Engineering* **70,** 327–333 (2010).

124. Joye, S. B., MacDonald, I. R., Leifer, I. & Asper, V. Magnitude and oxidation potential of hydrocarbon gases released from the BP oil well blowout. *Nature Geoscience* **4,** 160–164 (2011).

125. Tao, Q., Checkai, D., Huerta, N. & Bryant, S. L. An improved model to forecast CO2 leakage rates along a wellbore. *Energy Procedia* **4,** 5385–5391 (2011).

126. Tao, Q. & Bryant, S. L. Well permeability estimation and CO2 leakage rates. *International Journal of Greenhouse Gas Control* **22,** 77–87 (2014).

127. Le Guen, Y., Meyer, V., Poupard, O., Houdu, E. & Chammas, R. A Risk-Based Approach for Well Integrity Management Over Long Term in a CO2 Geological Storage Project. in (Society of Petroleum Engineers, 2009). doi:10.2118/122510-MS

128. Bai, M. Risk Assessment for CO_2 Leakage Along Abandoned Wells Using a Monte Carlo Simulation in a CO_2 Sequestration Site. *Petroleum Science and Technology* **32,** 1191–1200 (2014).

129. Viswanathan, H. S. *et al.* Development of a Hybrid Process and System Model for the Assessment of Wellbore Leakage at a Geologic CO_2 Sequestration Site. *Environmental Science & Technology* **42,** 7280–7286 (2008).

130. Humez, P., Audigane, P., Lions, J., Chiaberge, C. & Bellenfant, G. Modeling of CO2 Leakage up Through an Abandoned Well from Deep Saline Aquifer to Shallow Fresh Groundwaters. *Transport in Porous Media* **90,** 153–181 (2011).

131. Pawar, R. J., Watson, T. L. & Gable, C. W. Numerical Simulation of CO2 Leakage through Abandoned Wells: Model for an Abandoned Site with Observed Gas Migration in Alberta, Canada. *Energy Procedia* **1,** 3625–3632 (2009).

132. Celia, M. A., Nordbotten, J. M., Court, B., Dobossy, M. & Bachu, S. Field-scale application of a semi-analytical model for estimation of CO2 and brine leakage along old wells. *International Journal of Greenhouse Gas Control* **5,** 257–269 (2011).

133. Nogues, J. P., Court, B., Dobossy, M., Nordbotten, J. M. & Celia, M. A. A methodology to estimate maximum probable leakage along old wells in a geological sequestration operation. *International Journal of Greenhouse Gas Control* **7,** 39–47 (2012).

134. Checkai, D., Bryant, S. & Tao, Q. Towards a Frequency Distribution of Effective Permeabilities of Leaky Wellbores. *Energy Procedia* **37,** 5653–5660 (2013).

135. Gasda, S. E., Celia, M. A., Wang, J. Z. & Duguid, A. Wellbore Permeability Estimates from Vertical Interference Testing of Existing Wells. *Energy Procedia* **37,** 5673–5680 (2013).

136. Bennion, B. & Bachu, S. Drainage and Imbibition Relative Permeability Relationships for Supercritical CO2/Brine and H2S/Brine Systems in Intergranular Sandstone, Carbonate, Shale, and Anhydrite Rocks. *SPE Reservoir Evaluation & Engineering* **11,** 487–496 (2008).

137. Bachu, S. Drainage and Imbibition CO2/Brine Relative Permeability Curves at in Situ Conditions for Sandstone Formations in Western Canada. *Energy Procedia* **37,** 4428–4436 (2013).

138. Shi, J.-Q., Xue, Z. & Durucan, S. Supercritical CO2 core flooding and imbibition in Berea sandstone — CT imaging and numerical simulation. *Energy Procedia* **4,** 5001–5008 (2011).

139. Shi, J.-Q., Xue, Z. & Durucan, S. Supercritical CO2 core flooding and imbibition in Tako sandstone—Influence of sub-core scale heterogeneity. *International Journal of Greenhouse Gas Control* **5,** 75–87 (2011).

140. Pentland, C. H., El-Maghraby, R., Iglauer, S. & Blunt, M. J. Measurements of the capillary trapping of super-critical carbon dioxide in Berea sandstone: MEASUREMENT OF CARBON DIOXIDE ENTRAPMENT. *Geophysical Research Letters* **38,** n/a-n/a (2011).

141. Krevor, S. C. M., Pini, R., Zuo, L. & Benson, S. M. Relative permeability and trapping of CO_2 and water in sandstone rocks at reservoir conditions: MULTIPHASE FLOW OF CO_2 AND WATER IN SANDSTONE ROCKS. *Water Resources Research* **48,** (2012).

142. Mackay, E., Pickup, G. & Olden, P. Rock mechanics, geochemistry and aquifer fluid flow. in *CASSEM Conference, 4th October 2010* (2010).

143. Shell. *UK Carbon Capture and Storage Demonstration Competition.* 59 (ScottishPower CCS Consortium, 2011).

144. Juanes, R., Spiteri, E. J., Orr, F. M. & Blunt, M. J. Impact of relative permeability hysteresis on geological CO_2 storage: IMPACT OF HYSTERESIS ON GEOLOGICAL CO_2 STORAGE. *Water Resources Research* **42,** n/a-n/a (2006).

145. Sifuentes, W. F., Giddins, M. A. & Blunt, M. J. Modeling CO2 Storage in Aquifers : Assessing the key contributors to uncertainty. in (Society of Petroleum Engineers, 2009). doi:10.2118/123582-MS

146. Yang, Q. Dynamic Modelling of CO2 Injection in a Closed Saline Aquifer in the Browse Basin, Western Australia. in (Society of Petroleum Engineers, 2008). doi:10.2118/115236-MS

147. Bennion, D. B. & Bachu, S. Drainage and Imbibition CO2/Brine Relative Permeability Curves at Reservoir Conditions for High-Permeability Carbonate Rocks. in (Society of Petroleum Engineers, 2010). doi:10.2118/134028-MS
