# Supplementary material for: Estimating geological CO2 storage security to deliver on climate mitigation
Source: Nat Commun. 2018 Jun 12;9:2201. doi: 10.1038/s41467-018-04423-1 (PMC5997736; doi:10.1038/s41467-018-04423-1)
Supplement: Supplementary file 2 — Description of Additional Supplementary Files [file 41467_2018_4423_MOESM2_ESM.pdf]

Description of Additional Supplementary Files

File Name: Supplementary Data 1
Description: Storage Security Calculator R-code.
